# Supplementary material for: Claustral neurons projecting to frontal cortex restrict opioid consumption
Source: Curr Biol. 2023 Jul 10;33(13):2761–2773.e8. doi: 10.1016/j.cub.2023.05.065 (PMC10357322; doi:10.1016/j.cub.2023.05.065)
Supplement: Document S1. Figures S1–S6 and Table S1 [file mmc1.pdf]

Current Biology, Volume *33*

Supplemental Information

Claustral neurons projecting

to frontal cortex restrict opioid consumption

Anna Terem, Yonatan Fatal, Noa Peretz-Rivlin, Hagit Turm, Shahar Shohat Koren, Danny Kitsberg, Reut Ashwal-Fluss, Diptendu Mukherjee, Naomi Habib, and Ami Citri

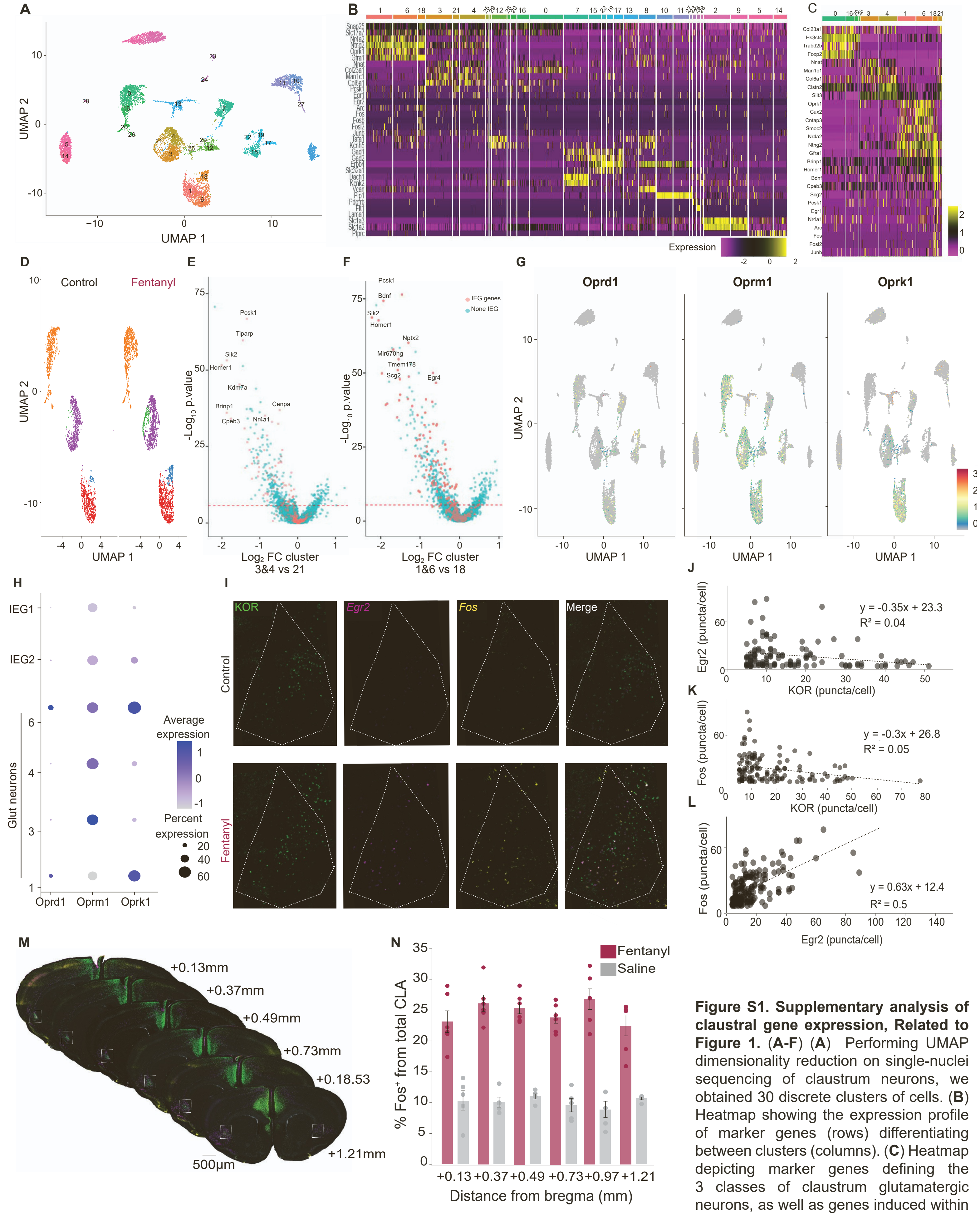

Figure S1. Supplementary analysis of claustral gene expression, Related to Figure 1. (**A-F**) (**A**) Performing UMAP dimensionality reduction on single-nuclei sequencing of claustrum neurons, we obtained 30 discrete clusters of cells. (**B**) Heatmap showing the expression profile of marker genes (rows) differentiating between clusters (columns). (**C**) Heatmap depicting marker genes defining the 3 classes of claustrum glutamatergic neurons, as well as genes induced within Glut IEG clusters (18,21). (**D**) IEG induction following fentanyl was observed in two sub-clusters of claustral glutamatergic neurons (green and blue dots relate to neurons associated with Glut IEG1 and Glut IEG2 clusters). The 3 parent clusters of glutamatergic neurons (labeled in orange, purple and red) are similar between control and fentanyl treatment. (**E**) Volcano plot, depicting genes differentially expressed between cluster 21 and the adjacent clusters 3,4. The Y axis depicts the -log p value (the dotted line corresponds to p=0.05), while the x axis depicts the log2 fold-change (FC) between clusters 3,4 and cluster 21. Differentially expressed genes are mostly upregulated in the IEG cluster. Red dots correspond to genes that are upregulated by activity in cultured neurons (data not shown) as an indication for the relation of the transcriptional differential between clusters 21 and 3,4 and gene inducibility. (**F**) as in (**E**) but for the comparison of cluster 18 to clusters 1,6. 89 of the top 100 differentially expressed genes in (**E**) are also found to be differentially expressed in (**F**), while 72 of the top 100 differentially expressed genes in (**F**) are also found to be differentially expressed in (**E**).

(**D**) Summary of virus expression and optic fiber locations in mice injected with retroAAV-CRE to the OFC, and AAV-DIO-GCaMP6s to the claustrum. The spread of infection was marked on histological sections, and digitally overlaid on an atlas image (n=5 mice). Right side – example image. (**E**) Overlayed average traces of ACCp signal corresponding to bouts of fentanyl consumption of different length (length=1, 1853 events; length=2, 619 events; length=3, 340 events; length=4, 198 events; length=5+, 714 events). As the delay between rewards in a bout could differ between trials, we averaged the signal around each reward in a ±1.5 seconds. Dotted lines extrapolate between rewards. (**F**) Overlayed average traces of ACCp signal corresponding to bouts of quinine consumption of different length (length=1, 1154 events; length=2, 134 events; length=3, 50 events; length=4, 36 events; length=5+, 69 events). (**G**) Average trace of the ACCp signal corresponding to a bout length=3, overlayed on the average trace of quinine bout length=3, illustrating the prolonged reduced activity in the ACCp activity between consecutive rewards in a bout. (**H**) Overlayed average traces of OFCp signal corresponding to bouts of fentanyl consumption of different length (length=1, 1082 events; length=2, 370 events; length=3, 261 events; length=4, 148 events; length=5+, 377 events). (**I**) Overlayed average traces of OFCp signal corresponding to bouts of quinine consumption of different length (length=1, 1206 events; length=2, 301 events; length=3, 140 events; length=4, 105 events; length=5+, 403 events). (**J**) Average trace of the OFCp signal corresponding to a bout length=3, overlayed on the average trace of quinine bout length=3, illustrating the recovery of the signal during both fentanyl and quinine consumption prior to each reward delivery. (**K-O**) The OFCp signal does not distinguish fentanyl from quinine consumption. (**K, M**) Heatmaps of OFCp signal during bouts of (**K**) fentanyl vs (**M**) quinine consumption of single rewards vs bouts (2+ rewards), in a window of ±5 second around the first reward (n=5 mice). (**L, N**) Average signal around single rewards compared to bouts of 2+ rewards. (**L**) Fentanyl; (**N**) Quinine. (**O**) Decrease in OFCp signal (average of 0 to +5 sec minus average of -5 to 0 sec) for single rewards vs bouts of consumption of fentanyl vs quinine. Liquid: p=0.2630, F(1, 4)=1.694; Is Bout: p=0.0253, F(1, 4)=12.137 ; Interaction: p=0.8118, F(1, 4)=0.065; ANOVA on LMM of delta signal ~ liquid * is bout | animal; n=5 mice.

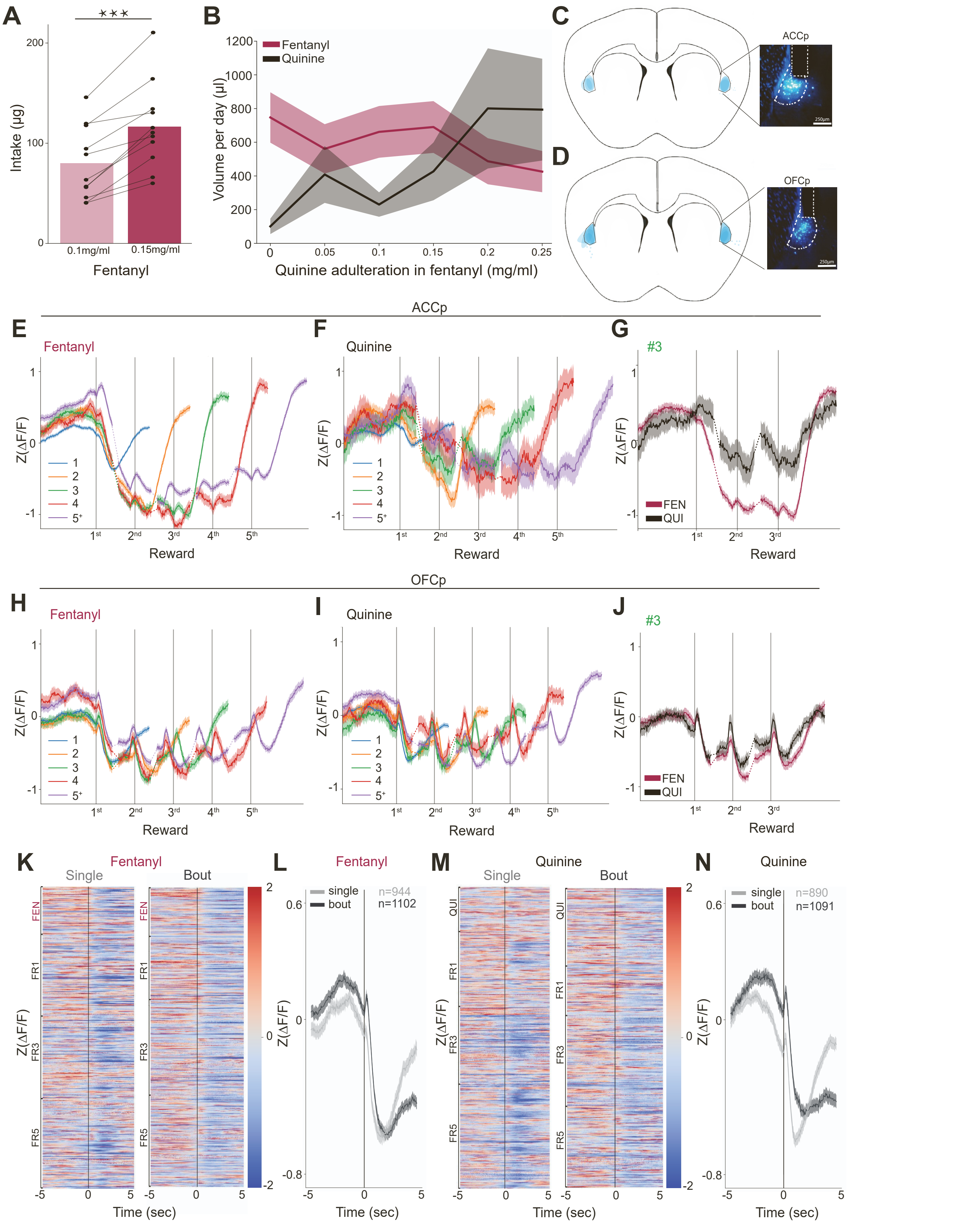

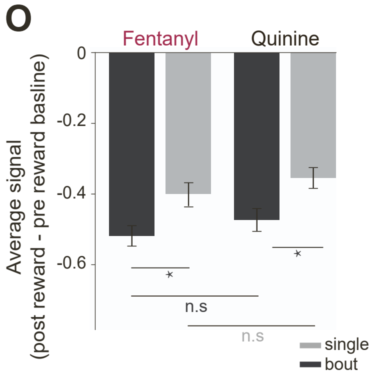

Figure S2. Supplementary analyses relating to fiber photometry recordings, Related to Figure 2. (**A-B**) Mice escalate consumption with increasing doses of fentanyl, and exhibit resilience to quinine-adulteration of fentanyl in the fiber photometry setup. (**A**) Average consumption of fentanyl at 0.1mg/ml = 79±11ug, fentanyl 0.15 mg/ml = 117±13ug: p=4.3e-05, t=6.877; paired t-test; n=11 mice. (**B**) Consumption of fentanyl (0.1 mg/ml) vs quinine (0.1 mg/ml) while escalating quinine adulteration of fentanyl (0, 0.1, 0.15, 0.2, 0.25 mg/ml). Only following addition of >0.2 mg/ml of quinine to fentanyl did mice reverse their fentanyl preference: p=0.0419, F(1, 4)=8.719; ANOVA on LMM of fentanyl volume ~ quinine dose | animal), while vehicle intake increased: p=0.0192, F(1, 4)=14.384; ANOVA on LMM of vehicle volume ~ quinine dose | animal); n=5 mice. ***p<0.001. (**C-J**) Relationship of bout consumption to signal deflection in ACCp & OFCp recordings. (**C**) Summary of virus expression and optic fiber locations in mice injected with retroAAV-CRE to the ACC, and AAV-DIO-GCaMP6s to the claustrum for the experiment shown in figure 2. The spread of infection was marked on histological sections, and digitally overlaid on an atlas image (n=5 mice). Right side – example image.

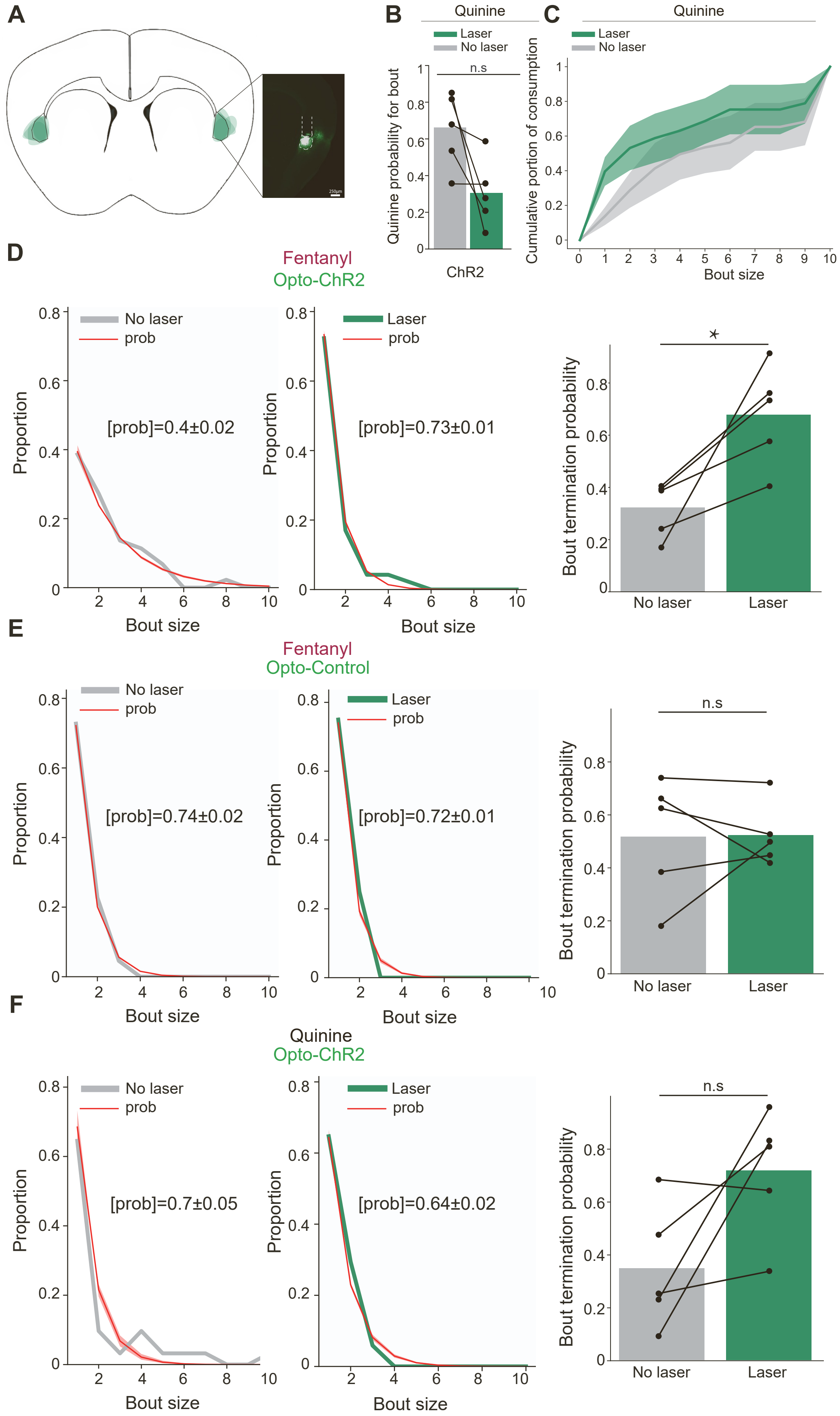

Figure S3. Optogenetic activation of frontal-projecting claustral neurons reduces bouts of quinine consumption, Related to Figure 3. (**A**) Summary of virus expression and optic fiber locations in mice injected with retroAAV-CRE to the ACC&OFC, and AAV-DIO-ChR2 to the claustrum for the experiment shown in **figure 3**. The spread of infection was marked on histological sections, and digitally overlaid on an atlas image (n=5 mice). Right side – example image. (**B**) Optogenetic activation reduces the probability that an individual trial of quinine consumption will result in a bout of consumption (2^+ rewards). p=0.0687, t=2.473; paired t-test; n=5 mice. (**C**) Cumulative distribution plot of quinine consumption (volume) as a function of bout length. Optogenetic stimulation caused mice to achieve more of their consumption in short bouts. (**D-F**) Modeling the distribution of bout lengths, by fitting a geometric function to each mouse in each condition. Each panel includes examples of individual mice, illustrating the proportion of bouts of different lengths (in grey or green) overlaid with the fit of a geometric function with a constant probability to terminate a bout (red). The probabilities are illustrated on the graphs as '[prob]'. On the right is the summary data, illustrating the bout termination probability for each individual mouse and the averages for the cohort. (**D**) Geometric functions fit to the consumption of fentanyl in mice expressing ChR2. p=0.0261, t=3.447; paired t-test; n=5 mice. (**E**) Geometric functions fit to the consumption of fentanyl in control mice. p=0.9659, t=0.045; paired t-test; n=5 mice. (**F**) Geometric functions fit to the consumption of quinine in mice expressing ChR2. p=0.0835, t=2.294; paired t-test; n=5 mice. n.s. non-significant, *p<0.05.

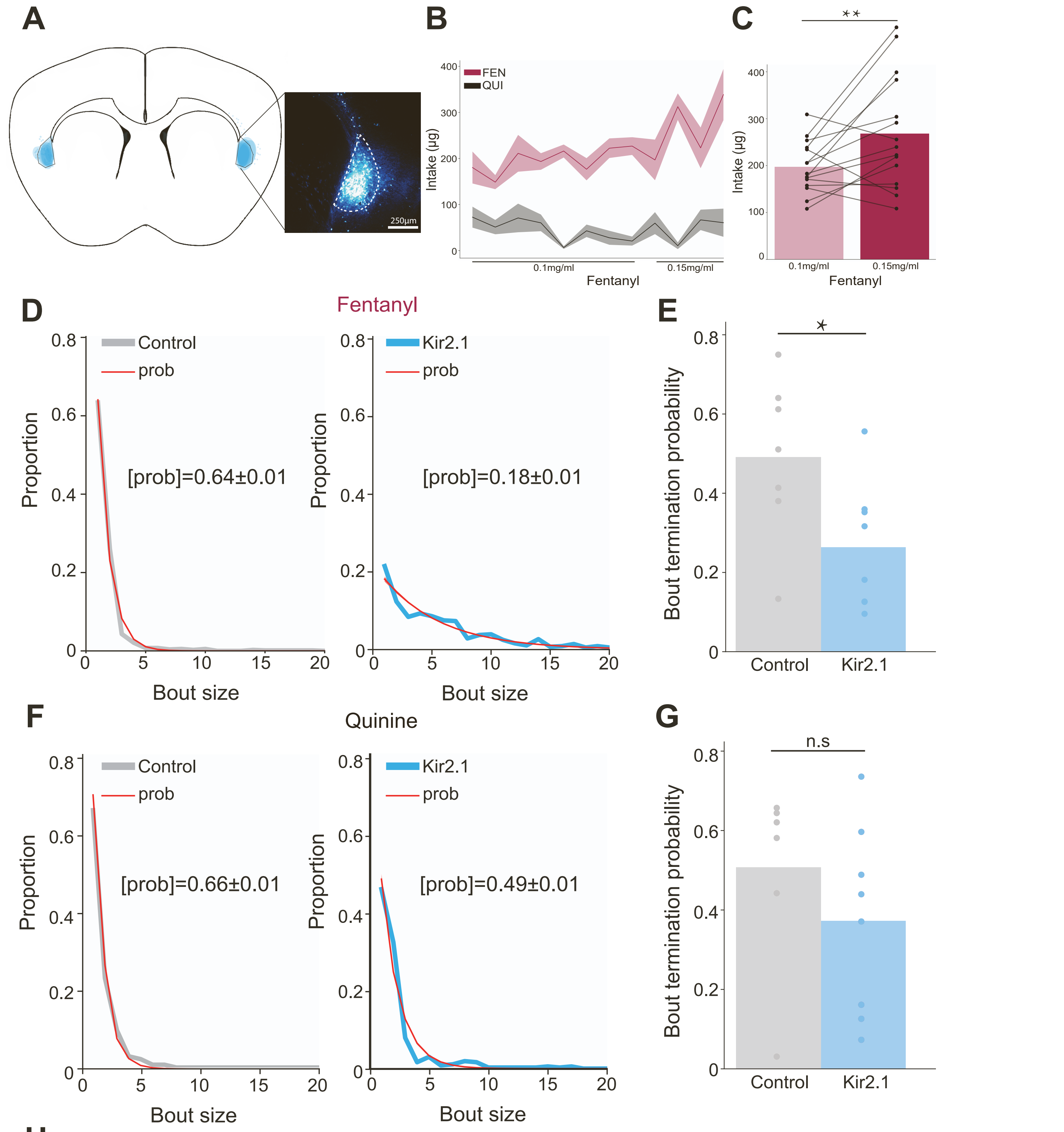

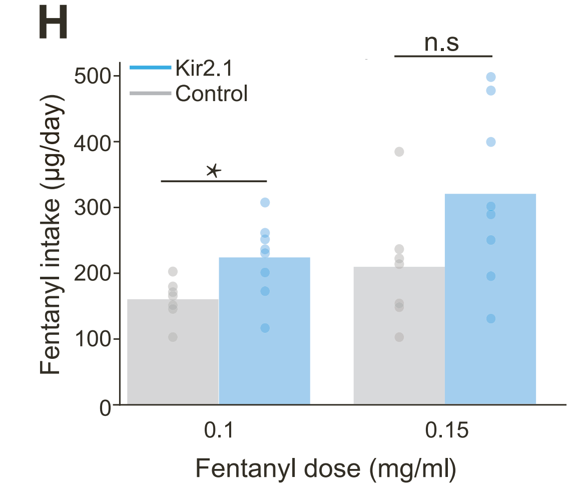

Figure S4. Mice escalate consumption with increasing doses of fentanyl in the automated cages, Related to Figure 4. (**A**) Summary of virus expression in mice injected with retroAAV-CRE to the ACC & OFC, and AAV-DIO-Kir2.1 to the claustrum for the experiment shown in **Figure 4**. The spread of infection was marked on histological sections, and digitally overlaid on an atlas image (n=8 ACC&OFC$^{Kir2.1}$ mice). Right side – example image. (**B-C**) Data illustrates the results for escalating doses of fentanyl (0.1 vs 0.15 mg/ml) consumption vs Quinine (0.1 mg/ml quinine) in the automated cages (n=15 mice). (**B**) Continuous graph of daily consumption. (**C**) Average daily consumption of fentanyl 0.1mg/ml = 197±9ug, fentanyl 0.15 mg/ml = 256± 23ug. p=0.0054, F(1, 14)=10.811; ANOVA on LMM – volume ~ dose | animal; n=15 mice. (**D-G**) Modeling the distribution of bout lengths, by fitting a geometric function to each mouse. (**D,F**) Each panel includes examples of individual mice (Kir2.1 vs control), illustrating the proportion of bouts of different lengths (in grey or blue) overlaid with the fit of a geometric function with a constant probability to terminate a bout (red). The probabilities are illustrated on the graphs as '[prob]'. (**D**) Geometric functions fit to the consumption of fentanyl in mice expressing Kir2.1 vs control. (**E,F**) Summary data, illustrating the geometric fit for each individual mouse and the averages for the cohort. (**E**) Fentanyl p=0.0310, t=2.418, t-test. (**F**) Quinine, p=0.2808, t=1.125; t-test. (**H**) Average daily consumption of fentanyl 0.1mg/ml, fentanyl 0.15 mg/ml (as in panel C), separated by stages for Kir2.1 vs control. 0.1mg/ml: p=0.0250, t=2.533; 0.15mg/ml: p=0.0878, t=1.771; t-test; n=8 Kir2.1 mice, n=7 control mice. n.s. non-significant, *p<0.05, **p<0.01.

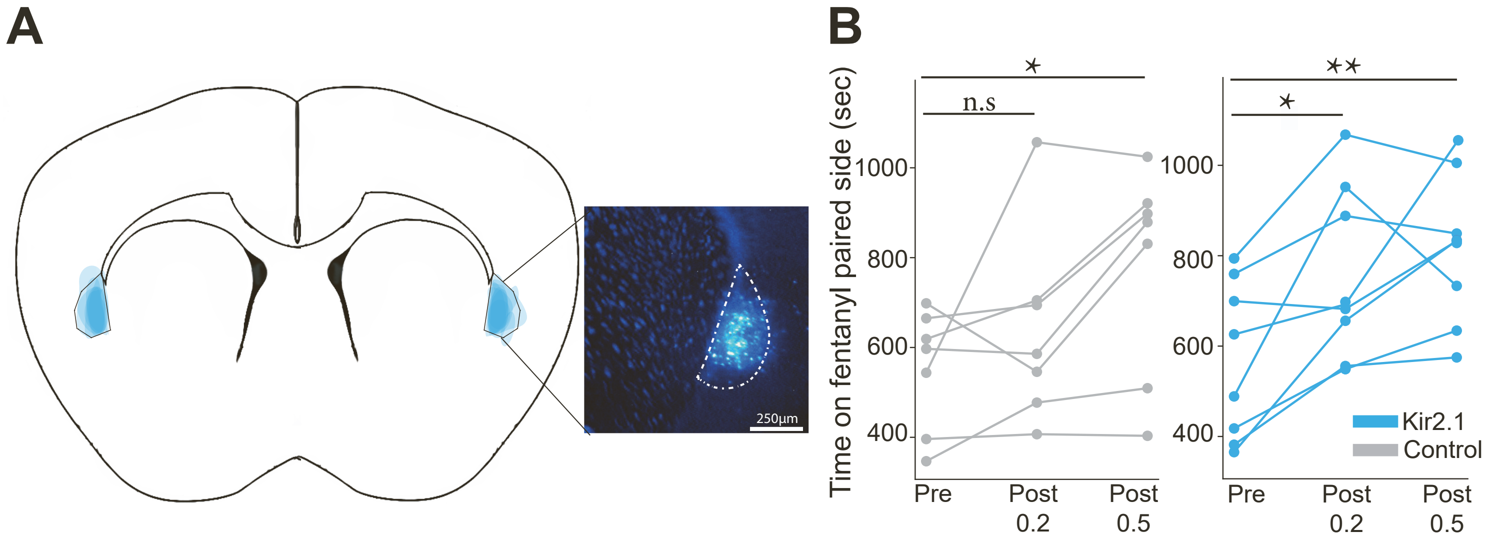

Figure S5. Fentanyl conditioned-place preference - time spent in fentanyl-paired side, Related to Figure 5. (**A**) Summary of virus expression in mice injected with retroAAV-CRE to the ACC & OFC, and AAV-DIO-Kir2.1 to the claustrum for the experiment shown in **Figure 5**. The spread of infection was marked on histological sections, and digitally overlaid on an atlas image (n=8 mice). Right side – example image. (**B**) Individual mice are depicted as connected dots, demonstrating time spent on the fentanyl paired side before exposure to fentanyl (Pre) and following exposure to low (0.2 mg/kg) and high (0.5 mg/kg) fentanyl concentrations. Kir2.1: low-dose, p=0.0188, t=3.547; high-dose, p=0.0022, t=5.289; paired t-test. Control: low dose, p=0.6250, t=1.102; high-dose, p=0.0136, t=4.04; paired t-test. Left: control, n=7 mice, Right: ACCp&OFCp$^{Kir2.1}$, n=8, paired t-test. n.s.=non-significant, *p<0.05, **p<0.01.

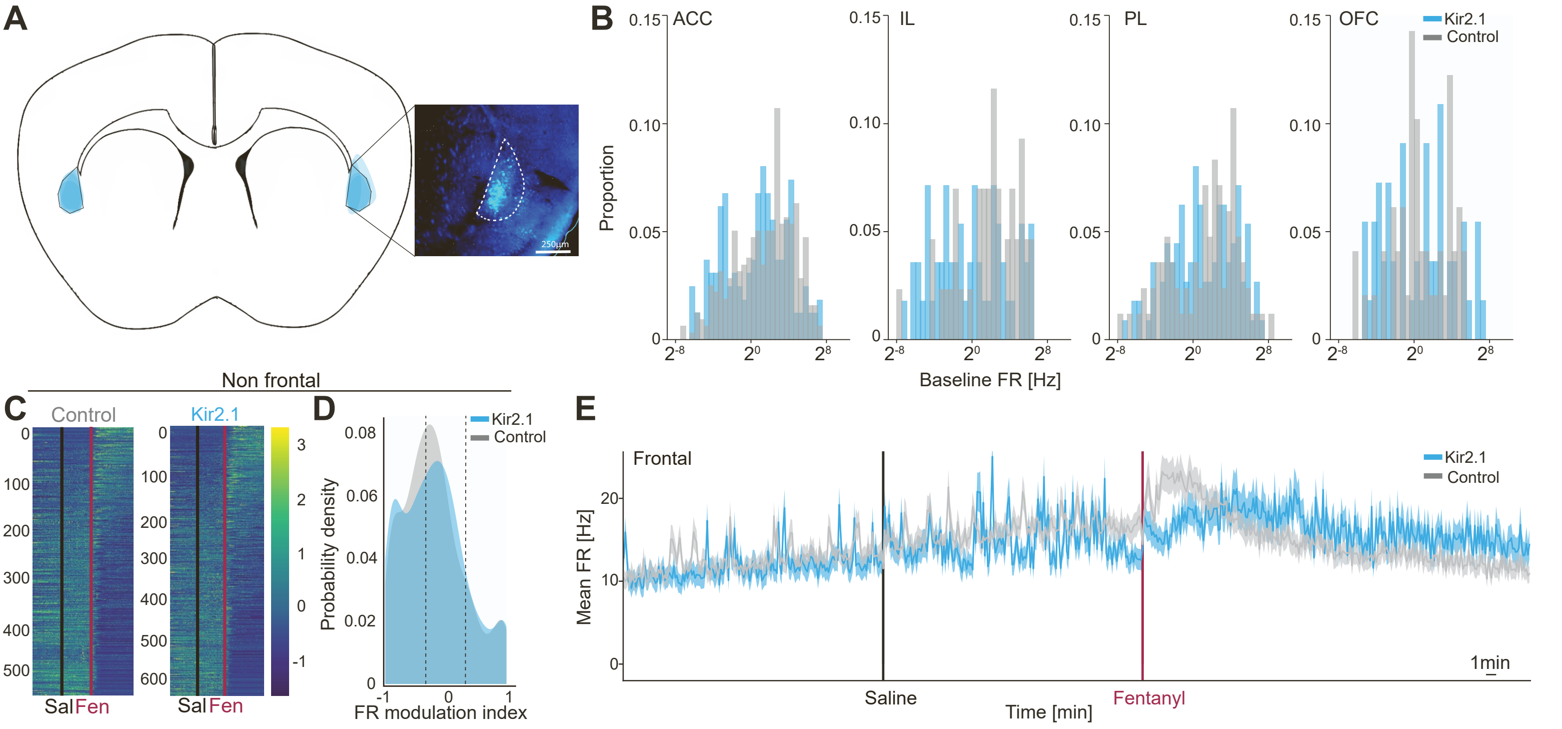

Figure S6. Inhibition of frontal-projecting claustral neurons specifically affects fentanyl modulation of the ACC & IL, Related to Figure 6. (**A**) Summary of virus expression in mice injected with retroAAV-CRE to the ACC&OFC, and AAV-DIO-Kir2.1 to the claustrum for the experiment shown in **Figure 6**. The spread of infection was marked on histological sections, and digitally overlaid on an atlas image (n=5 mice). Right side – example image. (**B**) Histograms of the distribution of firing rate (FR) at baseline, across different frontal structures (ACC = anterior cingulate cortex; IL = infralimbic cortex; PL = prelimbic cortex; OFC = orbitofrontal cortex), comparing ACCp&OFCp$^{Kir2.1}$ mice to control mice. Baseline FR was reduced in the ACC (p=0.0050, k-stat=7.883) and IL (p=0.0084, k-stat=6.952) of Kir2.1 animals, and unaffected in the PL (p=0.7737, k-stat=0.083) and OFC (p=0.6602, k-stat=0.193); Kruskal-Wallis test. (**C**) Heatmaps depicting normalized firing rates of individual units as a function of time. Units shown are located in non-frontal regions. (left) Control mice. (right) Experimental mice, expressing Kir2.1 in frontal-projecting claustral neurons. The black and red vertical lines denote the time of saline and fentanyl s.c. injection. (**D**) Distribution of fentanyl modulation indices, in recordings of non-frontal units from experimental (ACCp&OFCp$^{Kir2.1}$-expressing) mice vs control mice. Control: -0.19±0.02, Kir2.1: -0.18±0.02; p=0.73, K-stat=0.122; Kruskal-Wallis. (**E**) Mean firing rate (FR) of frontal units throughout the recording (20 min baseline, 20 min post-saline, 30 minutes post-fentanyl), illustrating the brief response of frontal units in the ~5 minutes post-fentanyl exposure in control mice, absent in experimental (ACCp&OFCp$^{Kir2.1}$) mice. In addition, the prolonged excitation of frontal units in experimental mice is observed, contrasting with the reduced activity of frontal units in

Figure	Mice, Groups, Group sizes	Statistical analysis
1B, D	Fentanyl; n=11 sections from 2 mice;<br>Saline; n=10 sections from 2 mice	Fos: control: 6±0.5%, fentanyl: 12.5±1.3%, $F(1, 2)=25.132$, *$p=0.0375$, ANOVA on linear mixed-effect model (LMM) of %IEG ~ treatment | animal.<br>Egr2: control: 7.8±0.5%, fentanyl: 12.9±0.7%, $F(1, 2)=42.807$, *$p=0.0226$, ANOVA on LMM of %IEG ~ treatment | animal.
1L	Fentanyl n=6 mice, n=70 sections;<br>Saline n=5 mice, n=56 sections	ANOVA on LMM of percent_positive ~ ap_loc + reference_total | animal<br>(reference_total – percent from DAPI or from FOS)<br>ACC, not OFC: $F(1, 5)=45.212$, **$p=0.0011$<br>ACC & OFC: $F(1, 5)=95.837$, ***$p=0.0002$<br>OFC, not ACC: $F(1, 5)=32.141$, **$p=0.0024$<br>Complement: $F(1, 5)=231.209$, ***$p=2.2e-05$
2C	n=11 mice; ACCp n=5, OFCp n=6	Liquid: $F(1,10)=93.182$, ***$p=2.2e-06$, Stage: FR3 – $F(1, 10)=4.315$, $p=0.0645$;FR5 – $F(1, 10)=3.512$, $p=0.0904$.<br>ANOVA on LMM of Volume ~ Liquid + Stage | Animal
2D	OFCp n=6 mice	Fentanyl intake:<br>$F(1, 5)=16.196$, *$p=0.0101$, ANOVA on LMM of amount ~ dose | animal<br>Water intake:<br>$F(1, 5)=16.541$, **$p=0.0097$, ANOVA on LMM of volume ~ dose | animal
2E	n=11 mice; ACCp n=5, OFCp n=6	ANOVA on LMM of %drinking in single rewards ~ liquid | animal. $F(1, 10)=105.837$, ***$p=1.2e-06$
2M	ACCp n=5 mice	ANOVA on LMM of delta_signal ~ liquid * is_bout | animal.<br>Liquid: $F(1, 4)=18.230$, *$p=0.0130$;<br>Is_Bout: $F(1, 4)=222.709$, ***$p=1.2e-04$; Interaction (liquid * is_bout): $F(1, 4)=15.291$, *$p=0.0174$
3C	ChR2 n=5 mice<br>Control n=5 mice	ChR2: $t=3.378$, $p=0.0278$, paired t-test<br>Control: $t=0.200$, $p=0.8512$, paired t-test
4D	Kir2.1 n=8 mice, Control n=7 mice	$F(1, 13)=6.944$, *$p=0.0206$, ANOVA on LMM of Volume ~ Group | Animal
4E	Kir2.1 n=8 mice, Control n=7 mice	$F(1, 13)=0.0001$, n.s, $p=0.9924$, ANOVA on LMM of Volume ~ Group | Animal
4H	Kir2.1 n=8 mice, Control n=7 mice	Single: $t=2.415$, *$p=0.0312$, Bout: $t=3.992$, **$p=0.0015$, t-test

4I	Kir2.1 n=8 mice,<br>Control n=7 mice	Single: t=1.447, n.s, p=0.1716, Bout: t=0.304 n.s, p=0.7660, t-test
5C	Kir2.1 n=8 mice	Kir2.1: low-dose, t=3.547, *p=0.0188; high-dose, t=5.289, **p=0.0022; paired t-test
5C	Control n=7 mice	Control: low dose, t=1.102, n.s., p=0.6250; high-dose, t=4.04, *p=0.0136; paired t-test
6H	Kir2.1 n=429 units,<br>Control n=532 units	Kruskal-statistic=40.271, ***p=2.2e-10. Kruskal-Wallis test.
6I	Kir2.1 n=5 mice,<br>Control n=4 mice	Frontal units: Upregulated X-stat=27.505, ***p=1.6e-07; Chi-squared test<br>Downregulated X-stat=19.922, ***p=2.2e-10; X^2-test for proportions.
6J	Kir2.1 n=5 mice,<br>Control n=4 mice	Other units: Upregulated X-stat=6.4e-06, p=0.9980; Chi-square test<br>Downregulated X-stat=0, p=1<br>X^2-test for proportions.
6K	Kir2.1 n=5 mice,<br>Control n=4 mice	X^2-test for proportions.<br>**ACC**<br>Down; Control=133/317, Kir2.1=43/162, stat=-3.31, ***p=0.0009.<br>Up, Control=38/317, Kir2.1=48/162, stat=4.76, ***p=2e-06.<br>**IL**<br>Down, Control=13/43, Kir2.1=6/56, stat=-2.444, *p=0.0145.<br>Up, Control=11/43, Kir2.1=26/56, stat=2.125, *p=0.0336.<br>**PL**<br>Down, Control =16/84, Kir2.1=24/112, stat=0.409, p=0.6823.<br>Up, Control =30/84, Kir2.1=29/112, stat=-1.483, p=0.1380.<br>**OFC**<br>Down, Control =10/49, Kir2.1=8/55, stat=-0.789, p=0.4302.<br>Up, Control =11/49, Kir2.1=21/55, stat=1.735, p=0.0823.
Supplemental Figures		
S1N	Fentanyl n=6 mice, 70 sections, Saline n=5 mice, 56 sections	ANOVA on LMM of percent _*Fos*$^+$ ~ ap_loc + treatment | animal Treatment: F(1, 9)=309.635, ***p=2.8e-08; AP position: F(1, 9)=0.166, n.s., p=0.6932;
S2A	n=11 mice; ACCp n=5, OFCp n=6	t=6.877, p=4.3e-05; paired t-test; n=11 mice
S2B	n=5 mice	Quinine adulteration experiment.<br>ANOVA on LMM of fentanyl volume ~ quinine dose | animal. F(1, 4)=8.719, *p=0.0419.<br>ANOVA on LMM of vehicle solution volume ~ quinine dose | animal. F(1, 4)=14.384, *p=0.0192

S2O	OFCp, n=5 mice	LMM of delta_signal ~ is_bout * liquid | animal<br>Liquid F(1, 4)=1.694, p=0.2630<br>Is_Bout F(1, 4)=12.137,*p=0.0253<br>Interaction F(1, 4)=0.065, p=0.8118
S3D	ChR2, n=5 mice	No laser, [prob]=0.320±0.048,<br>Laser [prob]=0.678±0.087,<br>t=3.447, *p=0.0261, paired t-test
S3E	Tdt, n=5 mice	No laser, [prob]=0.518±0.103,<br>Laser [prob]=0.523±0.053,<br>t=0.045, p=0.9659, paired t-test
S3F	ChR2, n=5 mice	No laser, [prob]=0.348±0.104,<br>Laser [prob]=0.717±0.107,<br>t=2.294, p=0.0835, paired t-test
S4C	n=15 mice:<br>(n=8 ACC&OFC$^{Kir2.1}$<br>n=7 ACC&OFC^{eGFP})	ANOVA on LMM of fentanyl amount ~ dose | animal.<br>**p=0.0054, F(1, 14)=10.812, **p=0. F(1, 14)=10.812
S4E	Kir2.1 n=8 mice,<br>Control n=7 mice	Fentanyl, Kir2.1 [prob]=0.264±0.056, GFP<br>[prob]=0.491±0.077, t=2.418, *p=0.0310, t-test
S4G	Kir2.1 n=8 mice,<br>Control n=7 mice	Quinine, Kir2.1 [prob]=0.374±0.084, GFP<br>[prob]=0.508±0.084, t=1.125, p=0.2808, t-test
S4H	Kir2.1 n=8 mice,<br>Control n=7 mice	0.1 mg/ml: t=2.533, *p=0.0250<br>0.15 mg/ml: t=1.771, p=0.0878<br>t-test
S5B	Kir2.1 n=8 mice<br>Control n=7 mice	Kir2.1: low-dose, t=3.547, *p=0.0188; high-dose, t=5.289, **p=0.0022; paired t-test<br>Control: low dose, t=1.102, n.s., p=0.6250; high-dose, t=4.04, *p=0.0136; paired t-test
S6B	Kir2.1 n=5 mice,<br>Control n=4 mice	**ACC**: k-stat=7.883, **p=0.0050<br>**IL**: k-stat=6.952, **p=0.0084<br>**PL**: k-stat=0.083, p=0.7737<br>**OFC**: k-stat=0.193, p=0.6602<br>Kruskal-Wallis test
S6D	Kir2.1 n=5 mice,<br>Control n=4 mice	Average fentanyl modulation index in non-frontal regions.<br>control: -0.19±0.02, Kir2.1: -0.18±0.02;<br>K-stat=0.122, p=0.73.<br>Kruskal-Wallis test.

TABLE S1.

Statistical analyses, Related to Figures 1, 2, 3, 4, 5, 6 and S1-S6.
