# Supplementary material for: Claustral neurons projecting to frontal cortex restrict opioid consumption
Source: Curr Biol. 2023 Jul 10;33(13):2761–2773.e8. doi: 10.1016/j.cub.2023.05.065 (PMC10357322; doi:10.1016/j.cub.2023.05.065)
Supplement: Document S2. Article plus supplemental information [file mmc2.pdf]

Current Biology

Article

Claustral neurons projecting to frontal cortex restrict opioid consumption

Graphical abstract

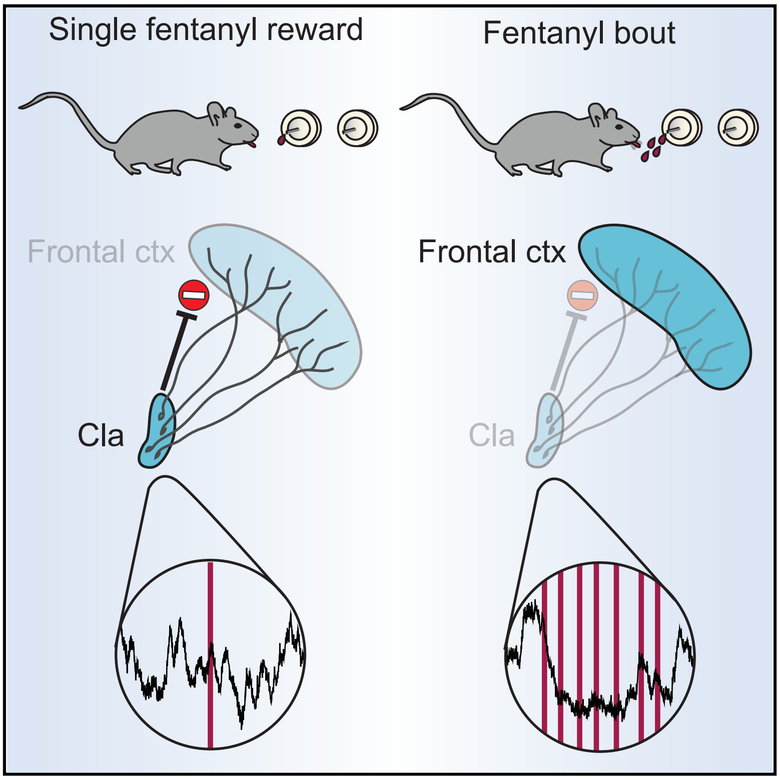

Authors

Anna Terem, Yonatan Fatal, Noa Peretz-Rivlin, ..., Diptendu Mukherjee, Naomi Habib, Ami Citri

Correspondence

ami.citri@mail.huji.ac.il

In brief

Terem and Fatal et al. study the claustrum's impact on opioid consumption. They discover that transient reductions in the activity of claustral neurons projecting to the frontal cortex are necessary for fentanyl bout consumption. Inhibition of these neurons disinhibits the frontal cortex and leads to prolonged consumption bouts of fentanyl.

Highlights

- Claustrum neurons are transiently repressed during bouts of fentanyl consumption
- Fentanyl bout consumption is limited by optogenetic activity of claustrum neurons
- Suppressing claustro-frontal projections increases fentanyl seeking and consumption
- Fentanyl drives enhanced activity of frontal neurons following claustrum suppression

Terem et al., 2023, Current Biology *33*, 2761–2773

July 10, 2023 © 2023 The Author(s). Published by Elsevier Inc.

https://doi.org/10.1016/j.cub.2023.05.065

Current Biology

CellPress
OPEN ACCESS

Article

Claustral neurons projecting to frontal cortex restrict opioid consumption

Anna Terem,[1,2,4] Yonatan Fatal,[1,4] Noa Peretz-Rivlin,[1] Hagit Turm,[1,2] Shahar Shohat Koren,[1] Danny Kitsberg,[1] Reut Ashwal-Fluss,[1] Diptendu Mukherjee,[1,2] Naomi Habib,[1] and Ami Citri[1,2,3,5,6,*]

[1]Edmond and Lily Safra Center for Brain Sciences, The Hebrew University of Jerusalem, Edmond J. Safra Campus, Givat Ram, Jerusalem 9190401, Israel

[2]Institute of Life Sciences, Hebrew University of Jerusalem, Edmond J. Safra Campus, Givat Ram, Jerusalem 9190401, Israel

[3]Program in Child and Brain Development, Canadian Institute for Advanced Research, MaRS Centre, West Tower, 661 University Avenue, Suite 505, Toronto, ON M5G 1M1, Canada

[4]These authors contributed equally

[5]Twitter: @CitriAmi

[6]Lead contact

*Correspondence: ami.citri@mail.huji.ac.il

https://doi.org/10.1016/j.cub.2023.05.065

SUMMARY

The synthetic opioid fentanyl is a major contributor to the current opioid addiction crisis. We report that claustral neurons projecting to the frontal cortex limit oral fentanyl self-administration in mice. We found that fentanyl transcriptionally activates frontal-projecting claustrum neurons. These neurons also exhibit a unique suppression of Ca^{2+} activity upon initiation of bouts of fentanyl consumption. Optogenetic stimulation of frontal-projecting claustral neurons, intervening in this suppression, decreased bouts of fentanyl consumption. In contrast, constitutive inhibition of frontal-projecting claustral neurons in the context of a novel, group-housed self-administration procedure increased fentanyl bout consumption. This same manipulation also sensitized conditioned-place preference for fentanyl and enhanced the representation of fentanyl experience in the frontal cortex. Together, our results indicate that claustrum neurons exert inhibitory control over frontal cortical neurons to restrict oral fentanyl intake. Upregulation of activity in the claustro-frontal projection may be a promising strategy for reducing human opioid addiction.

INTRODUCTION

Fentanyl is a powerful synthetic opiate and a major driver of the current opioid crisis, responsible for over 100,000 deaths in 2021 in the United States alone.[1–3] The continued worldwide rise of fentanyl-related deaths motivates detailed investigation into the brain mechanisms regulating fentanyl consumption.

Opioids, including fentanyl, modulate neuronal activity in the anterior cingulate (ACC) and orbitofrontal (OFC) cortical regions of both humans and rodents.[4,5] The claustrum is the brain region most highly enriched with kappa opioid receptor (KOR) expression,[6,7] an opioid receptor involved in escalation of opioid self-administration and stress-induced reinstatement of drug seeking.[8–11] Furthermore, acute exposure to morphine induces expression of the IEG c-Fos in the claustrum and ACC.[12]

The claustrum regulates the activity of pyramidal neurons in the frontal cortex,[13–16] and this claustro-frontal circuit contributes to multiple cognitive functions, including impulse control, attention, cognitive flexibility, incentive salience, and optimal performance under cognitive load.[13,16–21] Based on the observations that claustral and frontal neurons are recruited by opioids, the inhibitory control of the frontal cortex by the claustrum, and the importance of the claustro-frontal circuit in cognitive control, we hypothesized that claustro-frontal projections exert inhibitory control over the frontal cortex to regulate opioid intake.

To test this hypothesis, we used several approaches, including single-cell transcriptional profiling, projection-specific photometry, optogenetics, and constitutive inhibition of neuronal populations in the context of group-housed, individually monitored, oral fentanyl self-administration procedures in mice, as well as large-scale electrophysiological recordings. Our results support the conclusion that frontal-projecting claustral neurons restrict opioid consumption and reward by limiting the frontal representation of drug experience.

RESULTS

Fentanyl recruits frontal-projecting claustrum neurons

We applied single-molecule fluorescence *in situ* hybridization (smFISH) to measure the fentanyl-induced expression of the IEGs *Fos* and *Egr2* in the mouse claustrum, as a proxy for their transcriptional recruitment (Figure 1). Fentanyl (0.3 mg/kg intraperitoneally [i.p.]), as in Fujii et al.,[22] induced an increase in the number of *Egr2*⁺- and *Fos*⁺-expressing claustral neurons (*Fos*, control, 6% ± 0.5%; fentanyl, 12.5% ± 1.3%; *Egr2*, control, 7.8% ± 0.5%; fentanyl, 12.9% ± 0.7%; Figures 1A–1D). To define the identity of claustral neurons recruited by fentanyl, we applied single-nuclei RNA sequencing (snRNA-seq) (Figures 1E–1G and S1).[23] Clustering these data with a community detection algorithm, we obtained 30 discrete clusters of cells, which were

This is an open access article under the CC BY license (http://creativecommons.org/licenses/by/4.0/).

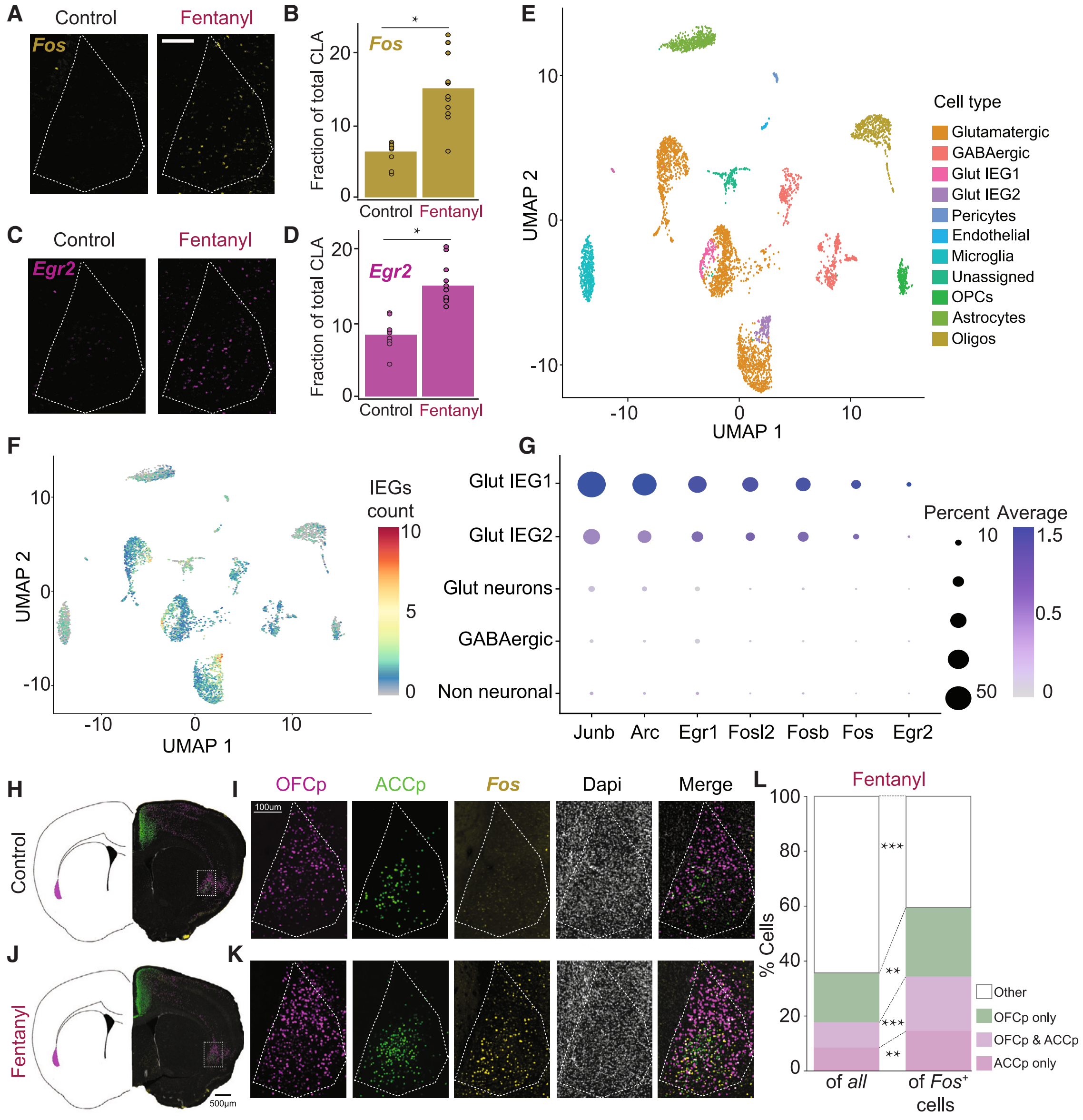

Figure 1. Fentanyl induces IEG expression in frontal-projecting claustral neurons

(A and C) Representative images of single-molecule fluorescence *in situ* hybridization (smFISH) for (A) *Fos* (yellow) or (C) *Egr2* (magenta) in slices from control mice (1 h after saline, left) or mice exposed to fentanyl (1 h after 0.3 mg/kg fentanyl i.p., right). Scale bar, 100 μm.

(B and D) Fraction of (B) Fos^{+}- or (D) $Egr2^{+}$-expressing cells of total claustrum cells. *Fos*, p = 0.0375, F(1, 2) = 25.132; Egr2, p = 0.0226, F(1, 2) = 42.807; linear mixed model (LMM) of %IEG ~ treatment|animal; n = 10–11 sections from 2 mice in each condition.

(E) Uniform manifold approximation and projection (UMAP) clustering of single-nuclei RNA-seq. The claustri of 14 mice were dissected, and 12,796 cells were harvested (n = 7 control; n = 7 collected 1 h after 0.3 mg/kg i.p. fentanyl) of which 10,457 cells passed quality controls (control = 5,096, fentanyl = 5,361). Clusters were assigned to cell types based on the following markers: glutamatergic neurons, *Slc17a7*; GABAergic neurons, *Gad1* and *Gad2*; astrocytes, *Slc1a3* and *Slc1a2*; oligodendrocytes, *Plp1*; endothelial cells, *Flt1*; fibroblasts, *Lama1*; pericytes, Pdgfrb; microglia, Ptprc; oligodendrocyte precursor cells (OPCs), *Can*; IEG-enriched populations (Glut IEG1 and Glut IEG2), *Egr1*, *Egr2*, *Fos*, *Nr4a1*, *Nr4a2*, *Fosb*, *Fosl2*, *Junb*, and *Arc*.

(F) IEG induction following fentanyl was observed primarily in two populations of claustral glutamatergic neurons (Glut IEG1 and Glut IEG2 clusters, as shown in E). UMAP representing the induction of the 7 IEGs (*Junb*, *Arc*, *Egr1*, *Fosl2*, *Fosb*, *Fos*, and *Egr2*) following exposure to fentanyl. Cells are color-coded by the sum of IEG count/cell.

(G) Dot-plot summarizing IEG expression following exposure to fentanyl across cell clusters. Dot size, fraction of IEG positive cells; color, average expression/cell.

(H and J) Representative images illustrating *Fos* induction in frontal-projecting claustrum neurons. Claustrum neurons projecting to OFC ("OFCp" neurons, retro-labeled following injection of AAV2-H2B-Ruby to the OFC) are labeled in magenta, claustrum neurons projecting to ACC ("ACCp" neurons, injection of AAV2-H2B-eGFP to the ACC) are labeled in green, immunohistochemistry against Fos in yellow, and DAPI in gray.

(H) Control (1.5 h following saline).

(J) Fentanyl (1.5 h following 0.3 mg/kg fentanyl).

(I and K) Individual channels and their merge, focusing on the claustrum. (I) Control and (K) fentanyl.

(legend continued on next page)

CellPress
OPEN ACCESS

assigned to cell types based on marker genes and visualized by uniform manifold approximation and projection (UMAP) embedding (Figure 1E). These clusters identify the major cell types comprising the claustrum, including excitatory glutamatergic projection neurons, inhibitory interneurons, astrocytes, microglia, oligodendrocytes, and oligo precursors, as well as other cell types (pericytes, endothelial cells, and unassigned cells) (Figures S1A and S1B).

Claustral glutamatergic neurons were found grouped in 3 distinct subpopulations (clusters 1, 6, and 18; clusters 3, 4, and 21; and clusters 0, 16, 20, and 26), which were distinguished by their differential expression of marker genes (Figure S1C). Following exposure to fentanyl, IEG induction was observed in subclusters within two of these three glutamatergic populations (clusters 18 and 21; Figures 1F, 1G, S1C, and S1D). IEG-expressing clusters differed from neighboring clusters in the expression of multiple genes, most of which were common to the two IEG clusters (cluster 18 versus 1 and 6 and cluster 21 versus 3 and 4; Figures S1E and S1F). Exploring the identity of differentially expressed genes by contrasting them with a dataset of genes induced by neural activity in cultured neurons (data not shown), we found that many of the differentially expressed genes corresponded to inducible genes (Figures S1E and S1F). Thus, we conclude that fentanyl induces IEG expression in subpopulations of excitatory claustrum neurons.

Because fentanyl is a ligand of opiate receptors (agonist at mu and partial agonist at kappa[24]), we addressed the possibility that fentanyl acts directly on the claustrum to induce IEG expression by measuring the expression of opiate receptors (*Oprd1*, *Oprk1*, and *Oprm1*) in claustral IEG clusters in comparison with surrounding clusters. We found that while *Oprd1* was only sparsely expressed, *Oprm1* and *Oprk1* were expressed in claustral glutamatergic neuronal clusters, encompassing a broader population than that defined by fentanyl-induced IEG expression (Figure S1G). Focusing on the IEG clusters (18 and 21) and adjacent glutamatergic clusters (1 and 6; 3 and 4), we observed that *Oprm1* exhibited some variance in expression across clusters, with no clear relation to IEG induction (Figure S1H). Interestingly, *Oprk1* was expressed selectively in clusters 1 and 6 (and not in 3 and 4) and was excluded from IEG cluster 18 (Figure S1H). Thus, *Oprk1* expression is observed in a defined population of claustral excitatory projection neurons and its expression within cells may exclude them from transcriptional recruitment by fentanyl. To further explore this observation, we applied smFISH to explore the relationship between fentanyl-induced IEG induction and *Oprk1* expression (Figure S1I) and observed a negative correlation between IEG (*Egr2* and *Fos*) induction and *Oprk1* expression (Figures S1J–S1L). Therefore, it seems that fentanyl-driven IEG induction in claustral neurons is likely mediated by an indirect mechanism. Additionally, the expression of KOR within claustrum glutamatergic neurons appears to dampen IEG expression following exposure to fentanyl.

Because frontal cortical structures are recruited by opioids and opioid-associated cues,[4,5,12,25–27] we tested whether fentanyl may be preferentially recruiting frontal-projecting claustral neurons. We labeled claustral neurons projecting to the anterior cingulate cortex (ACCp neurons) and orbitofrontal cortex (OFCp neurons) with fluorescent reporters encoded by retro-transporting viruses injected to these cortical sites (retroAAV-H2B-eGFP and retroAAV-H2B-ruby). We then measured the co-expression of these markers with fentanyl-induced c-Fos expression (assayed by immunohistochemistry). Exposure to fentanyl (0.3 mg/kg i.p.) induced c-Fos expression in claustrum cells (from 10.3% ± 0.4% to 24.6% ± 0.5%; Figures 1H–1L, S1M, and S1N), providing a measure of protein expression consistent with the observations obtained by two independent measures of smFISH and single-cell RNA-seq (scRNA-seq) (Figures 1 and S1A–S1L). c-Fos induction was enriched within frontal-projecting claustral neurons, such that while frontal-projecting claustral neurons account for 36% ± 1% of total claustral cells, they account for 60% ± 2% of Fos-expressing claustral neurons (Figure 1L).

Bout consumption of fentanyl is associated with a prolonged reduction in the activity of frontal-projecting claustral neurons

To probe the role of frontal-projecting claustral neurons in fentanyl consumption, we developed an oral fentanyl self-administration choice procedure in freely behaving mice, while performing optical recording of claustrum activity (Figure 2A). Mice were allowed to choose between fentanyl and quinine-adulterated water (0.1 mg/mL quinine, balancing the bitterness of fentanyl, similar to the midrange of quinine calculated in Monroe and Radke[28]; Figure 2B). In this procedure, mice immediately developed a preference for fentanyl (average consumption/day: fentanyl, 0.79 ± 0.03 mL; quinine-water, 0.33 ± 0.02 mL; Figure 2C). Fentanyl consumption further escalated with increased drug concentration (79 ± 11 μg/session of 0.1 mg/mL fentanyl versus 117 ± 13 μg/session of 0.15 mg/mL; Figure S2A). To ensure that the preference for fentanyl was driven by drug preference, rather than aversion to the quinine-adulterated water, we allowed mice to choose between fentanyl and their standard drinking water, in the context of an intermittent access procedure. Mice continued to consume fentanyl in this context and exhibited a concentration-dependent increase in fentanyl consumption, while reducing water intake (Figure 2D). Preference for fentanyl over quinine was maintained even following adulteration of fentanyl with quinine, to the extent that mice reverted to preferring the control port (with 0.1 mg/mL quinine) only following adulteration of the fentanyl port with a 2-fold higher concentration of quinine than that of the control port (≥0.2 mg/mL; Figure S2B). Furthermore, a large proportion of consumption, especially of fentanyl, occurred in bouts of multiple reward deliveries (bouts of 2+ rewards for fentanyl = 84% ± 1%, quinine = 54% ± 3%; n = 11 mice; Figure 2E).

(L) Distribution of ACCp and OFCp claustrum projection neurons as a fraction of Fos^+ cells versus total claustral cells. Of all cells, 7% ± 1% project only to ACC, 8% ± 1% project to both ACC and OFC, and 21% ± 1% project to OFC. Of Fos+ cells, 13% ± 1% project only to ACC, 17% ± 1% project to both ACC and OFC, and 30% ± 2% project to OFC. ACCp only, p = 0.0011, F(1, 5) = 45.212; ACCp and OFCp, p = 0.0002, F(1, 5) = 95.837; OFCp only, p = 0.0024, F(1, 5) = 32.141; non-frontal-projecting cells, p = 2.2e−05, F(1, 5) = 231.209). ANOVA on LMM of %projection ~ reference total (of DAPI or of *Fos*) +AP location|animal; fentanyl n = 6 mice, 70 sections, saline n = 5 mice, 56 sections. *p < 0.05, **p < 0.01, ***p < 0.001.
See also Figure S1 and Table S1.

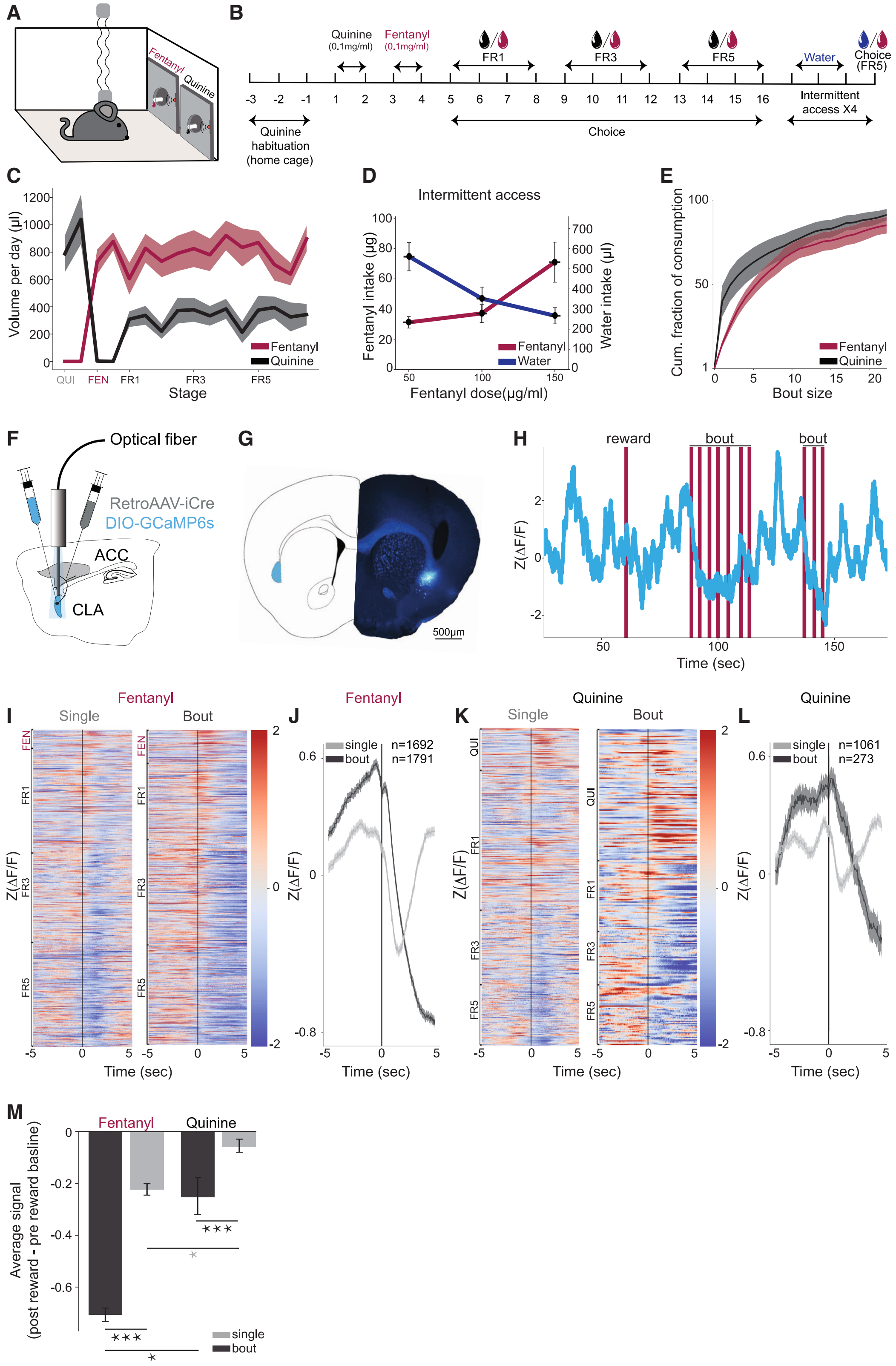

Figure 2. Bouts of fentanyl consumption are associated with unique signatures of ACCp activity

(A) Scheme of experimental setup. Mice are tethered to optic fibers while free to explore an arena in which two ports provide alternating liquids (quinine-adulterated water, fentanyl, or water).

(legend continued on next page)

Having established a reliable procedure of oral fentanyl self-administration in freely behaving mice, we recorded calcium signals associated with neural activity using fiber photometry. Recordings were restricted to ACCp neurons by injecting mice with the retrogradely transporting AAV virus expressing CRE (retroAAV-CRE) to the ACC, intersected with targeting the claustrum with AAV-DIO-GCaMP6s, encoding a CRE-dependent calcium indicator. Optic fibers were localized above the claustrum (Figures 2F–2M and S2C). ACCp activity was prominently modulated by reward consumption, evident in the representative *Z* scored signal trace (Figure 2H). The consumption of single fentanyl rewards was associated with a transient dip in the photometry signal following fentanyl delivery, which recovered to baseline within 5 s. In contrast, bouts of multiple fentanyl rewards ($\geq$2) were associated with a prolonged depression of activity following the delivery of the first fentanyl reward in a bout (Figures 2I, 2J, and S2E). Recovery of the ACCp signal to baseline levels occurred only following termination of the bout of consumption (Figure S2E).

Consumption of quinine-adulterated water within the same behavioral sessions was also associated with a depression of the ACCp signal (Figures 2K, 2L, and S2F). Although both the fentanyl and the quinine signals exhibited a larger signal deflection during bout consumption than following single rewards, ACCp signal deflection during fentanyl rewards was larger than that observed for quinine, both for single rewards as well as for bouts (Figures 2M and S2G). Performing similar recordings from OFCp neurons (Figure S2D), we found that the activity of these neurons exhibited a transient dip following consumption of single rewards of either fentanyl or quinine, which recovered following each reward in a bout (Figures S2H–S2J). Although the OFCp signal did differentiate bouts from single rewards, it did not differentiate fentanyl from quinine, contrasting with the ACCp signal (Figures S2K–S2O). In sum, mice rapidly establish a preference for oral self-administration of fentanyl, which they tend to consume in bouts of multiple rewards. Fentanyl consumption was associated with transient dips in the activity of claustral neurons projecting to both frontal targets (ACC or OFC), and a prolonged decrease in the activity of ACCp neurons, extending throughout the duration of the bout.

Acute stimulation of frontal-projecting claustral neurons decreases fentanyl bouts

To test the functional relevance of the dip in activity of frontal-projecting claustral neurons associated with bout consumption of fentanyl, we applied optogenetics. Optogenetic stimulation of ACCp and OFCp neurons after the first fentanyl reward in a trial was anticipated to counter the dip in activity observed during bouts. Performing this experiment, within the oral fentanyl self-administration choice procedure described in Figure 2, we randomly interleaved trials with and without optogenetic stimulation of ACCp and OFCp neurons, following the first fentanyl delivery in a trial.

This optogenetic stimulation significantly increased the probability that mice terminate their consumption after a single reward, rather than proceed to perform bouts of multiple rewards ($\geq$2) (Figure 3). In the experimental group (ACCp and OFCp^{ChR2}), optogenetic stimulation drove a contrast in the probability that trials would terminate following single rewards, such that in the presence of laser, the probability to terminate a bout following a single reward was 36% ± 11%, while in its absence it was 70% ± 5%. In contrast, laser stimulation did not affect consumption in the control group in which claustral neurons did not express ChR2; probability for single fentanyl rewards: 48% ± 5% (−laser) and 46% ± 10% (+laser) (Figure 3C).

The impact of optogenetic stimulation was further illustrated by plotting the cumulative fraction of consumption as a function of bout size. Laser stimulation of frontal-projecting claustral neurons drove a shift of the curve toward shorter drinking bouts. This effect was clearly seen for experimental (ChR2-expressing) mice on fentanyl consumption (Figures 3D and 3E), while it was absent in controls (Figures 3F and 3G). The impact of laser stimulation on quinine consumption followed a trend similar (albeit not

(B) Experimental timeline.

(C) Fentanyl and quinine consumption over 16 experimental days. Mice exhibited a prominent preference for fentanyl. Liquid, $p = 2.2e{-}06$, $F(1, 10) = 93.182$; stage FR3, $p = 0.0645$, $F(1, 10) = 4.315$; stage FR5, $p = 0.0904$, $F(1, 10) = 3.512$; ANOVA on LMM of volume ~ liquid + stage|animal; n = 11 mice. Volume (mL) was used as the metric for comparison between consumption of liquids (fentanyl versus quinine or water), while μg was used as the metric for absolute fentanyl consumption.

(D) Mice prefer fentanyl over water and increase their consumption as a function of increasing concentration. Fentanyl, $p = 0.0101$, $F(1, 5) = 16.196$; water, $p = 0.0097$, $F(1, 5) = 16.541$; ANOVA on LMM of fentanyl ~ dose|animal; n = 6 mice.

(E) Cumulative distribution plot of consumption as a function of bout length. Compared to quinine, mice consume fentanyl in longer bouts. Average percent of bouts of 2+ rewards for fentanyl = 84% ± 1%; quinine = 54% ± 3%. $p = 1.2e{-}06$, $F(1, 10) = 105.837$; ANOVA on LMM of % drinking in single rewards ~ liquid|animal; n = 11 mice.

(F) Scheme of virus injections. Mice were injected with retroAAV-Cre to the ACC, and Cre-dependent GCaMP6s to the claustrum. Optic fibers were positioned above the claustrum.

(G) Representative infection and fiber placement in an ACCp mouse.

(H) Representative *Z* scored ACCp photometry signal showing a robust decrease during bouts of consumption.

(I and K) Heatmaps illustrating the ACCp signal during (I) fentanyl consumption versus (K) quinine consumption, differentiating between single rewards and bouts (2+ rewards). Each row in the heatmap represents 10 s of continuous ACCp recording centered around the first reward (indicated by a black line) delivered in a trial.

(J and L) Average signal around single rewards versus bouts for (J) fentanyl versus (L) quinine. Fentanyl, n = 1,692 single rewards, n = 1,791 bouts; quinine, n = 1,061 single rewards, n = 273 bouts, from 5 mice.

(M) There is a more prominent dip in the ACCp signal (average signal from 0 to +5 s following a fentanyl reward minus average signal at pre-reward baseline, −5 to 0 s) during fentanyl bouts than during consumption of quinine or single fentanyl rewards. A similar pattern, albeit with modest signal deflections, is apparent for quinine. Liquid, $p = 0.0130$, $F(1, 4) = 18.230$; is bout, $p = 1.2e{-}04$, $F(1, 4) = 222.709$; interaction, $p = 0.0174$, $F(1, 4) = 15.291$; ANOVA on LMM of delta signal ~ liquid * is bout|animal, n = 5 mice. *$p < 0.05$, ***$p < 0.001$; error bars and shaded errors represent SEM.

See also Figure S2 and Table S1.

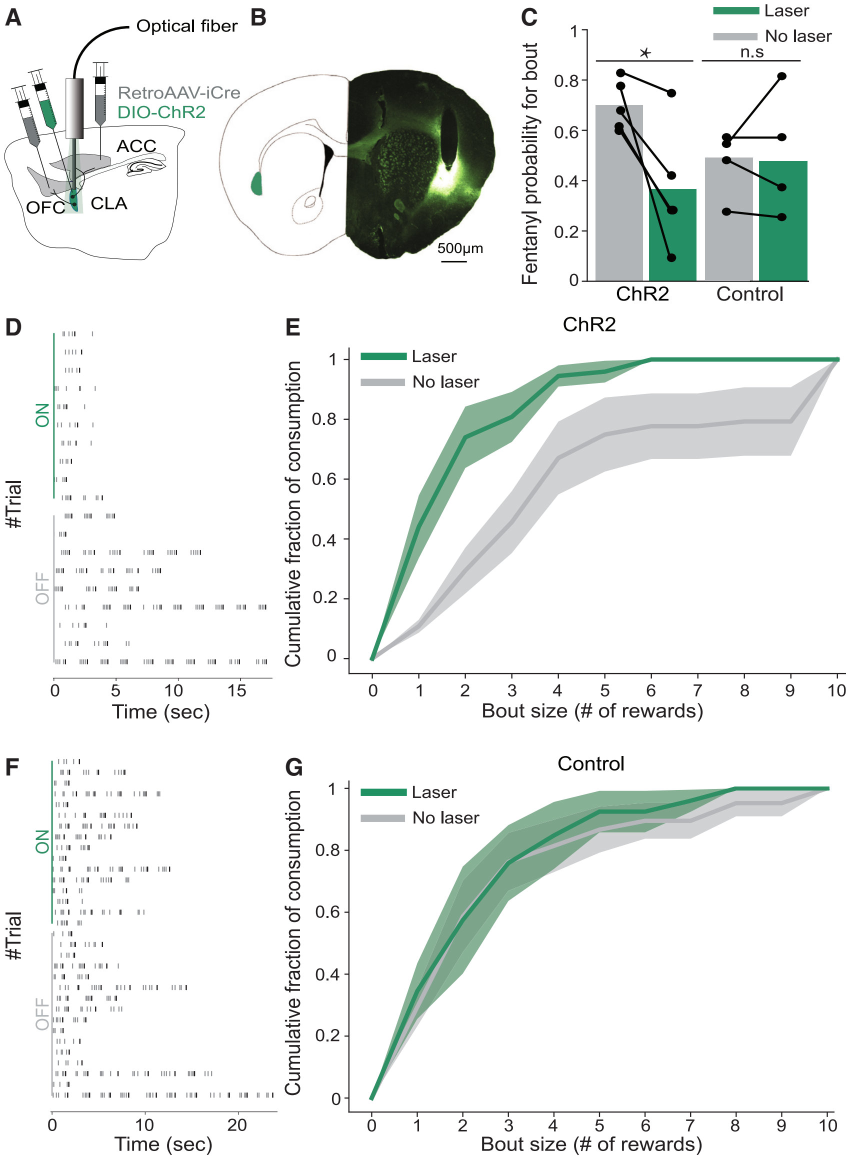

Figure 3. Acute stimulation of frontal-projecting claustral neurons limits bouts of fentanyl consumption

(A) Injection scheme. retroAAV-Cre in the ACC and OFC, and Cre-dependent ChR2 in the claustrum. Optic fibers were positioned above the claustrum.

(B) Representative infection and cannula implantation.

(C) Optogenetic stimulation decreased the probability of fentanyl consumption bouts. Laser stimulation was randomly interleaved in 50% of the trials. The laser was triggered by the 5th (rewarded) lick in a sequence of FR5, at 20 Hz for 2 s. Trials terminated following 10 rewards or >1 s with no lick detected. Probabilities for bout/trial: ChR2: 70% ± 5% (no laser); 36% ± 11% (laser); p = 0.0278, t = 3.378; paired t test; n = 5 mice; control: 48% ± 5% (no laser); 46% ± 10% (laser), p = 0.8512, t = 0.200; paired t test; n = 5 mice.

(D and F) Representative sessions, sorted by laser on versus off; each row represents a single trial. Each gray tick represents a single lick; black ticks represent reward. (D) ChR2 group versus (F) controls.

(E and G) Cumulative distribution plots of fentanyl consumption (volume) as a function of rewards in a bout. Optogenetic stimulation caused (E) ChR2 but not (G) control mice to achieve more consumption in short trials. n.s., non-significant; *$p < 0.05$; error bars and shaded errors represent SEM.

See also Figure S3 and Table S1.

statistically significant) to that observed for fentanyl, whereby the probability for bout consumption of multiple rewards of quinine-adulterated water shifted from 65% ± 9% (−laser) to 30% ± 9% (+laser) in the experimental group (Figure S3). A modest effect of optogenetic stimulation was also observed on the curve of quinine consumption in experimental mice (Figure S3C).

We further modeled the data, addressing the possibility that bout termination follows a geometric distribution for a given condition (i.e., each condition is characterized by a constant probability to terminate a drinking bout following the delivery of each reward = $p_{terminate}$). Indeed, fitting geometric functions to individual mice in the no laser versus laser conditions illustrated that laser stimulation increased the probability to terminate consumption (ChR2 fentanyl no laser [prob] = 0.32 ± 0.05 versus laser [prob] = 0.68 ± 0.09, Figure S3D). In control mice, laser stimulation had no impact on fentanyl consumption (no laser [prob] = 0.52 ± 0.1 versus laser [prob] = 0.52 ± 0.05, Figure S3E). The impact of laser stimulation on quinine consumption followed a similar trend (albeit not statistically significant; quinine no laser [prob] = 0.35 ± 0.1 versus laser [prob] = 0.72 ± 0.1, Figure S3F).

Together, the results of this experiment indicate that optogenetic activation of frontal-projecting claustral neurons following the first fentanyl reward in a bout decreased fentanyl intake by increasing the probability to terminate drinking bouts. As the experimental design was based on interleaved laser and no-laser trials, mice appeared to compensate for the reduction in bouts of multiple fentanyl rewards in the laser trials by prolonging bouts of multiple fentanyl rewards in the no-laser trials within the same session. Although more variable and not statistically significant, a similar trend was observed for the consumption of quinine-adulterated water, suggesting that in this setup, acute optogenetic stimulation of claustral neurons tended to terminate bout consumption, with some specificity toward fentanyl.

Chronic inhibition of frontal-projecting claustral neurons increases bouts of fentanyl self-administration

Our results associate the activity of frontal-projecting claustral neurons with the capacity to terminate bout consumption of fentanyl, suggesting that increased fentanyl consumption may be observed in cases in which the activity of these neurons is deficient. Humans tend to consume opioids in their home environment, escalating consumption over time.[29] Furthermore, the social environment is a major factor modulating drug

CellPress
OPEN ACCESS

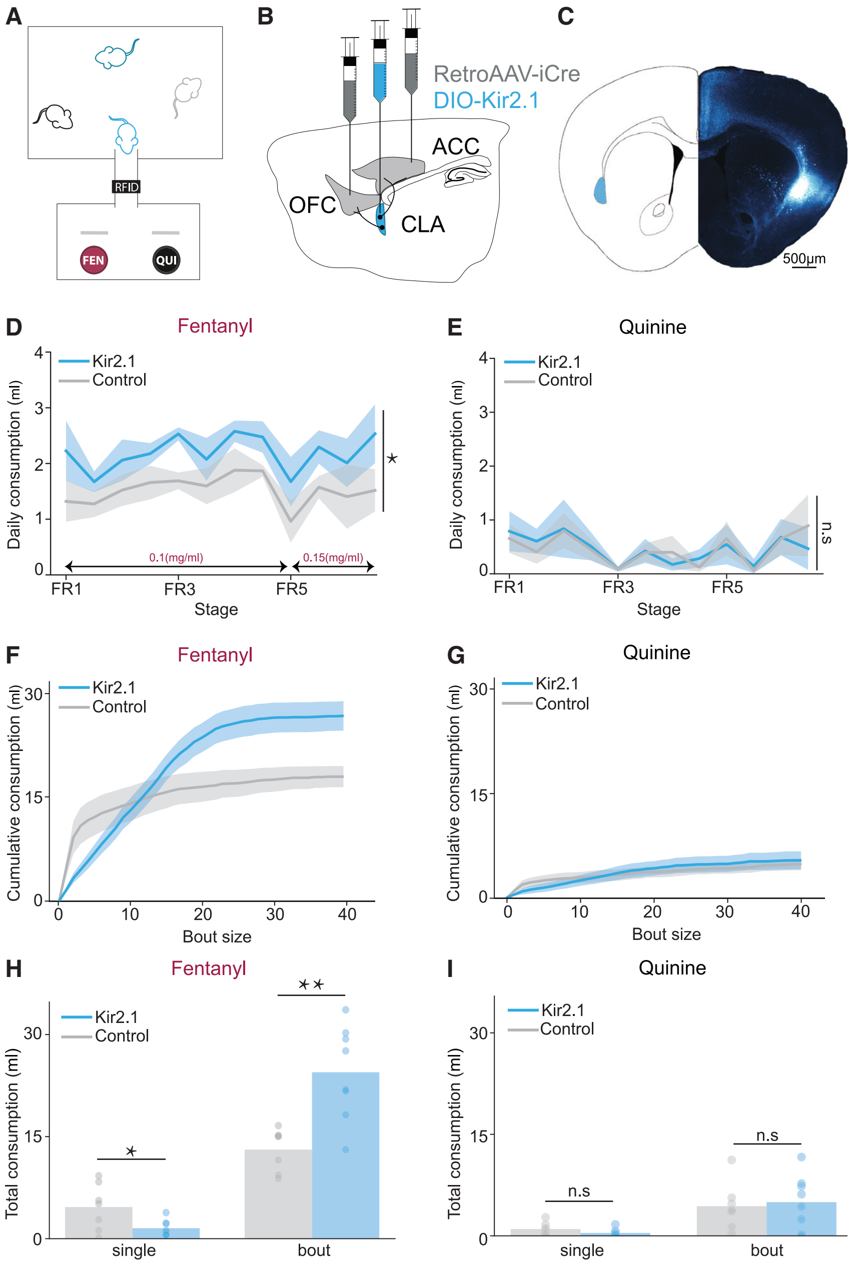

Figure 4. Chronic inhibition of frontal-projecting claustral neurons promotes bouts of fentanyl self-administration

(A) Experimental setup: mice are group-housed in their home-cage, with continuous access to fentanyl and quinine-adulterated water. An RFID reader adjacent to the ports supports monitoring of individual mice.

(B) Injection scheme. retroAAV-Cre in the ACC and OFC, and Cre-dependent Kir2.1 in the claustrum.

(C) Representative infection.

(D) ACCp and OFCp^{Kir2} mice consistently consumed more fentanyl across all experimental days. p = 0.0206, F(1, 13) = 6.944; ANOVA on LMM of volume ~ group|animal; Kir2.1 n = 8 mice, GFP n = 7 mice.

(E) ACCp and OFCp^{Kir2} mice drank similar volumes of quinine-adulterated water as the control group. p = 0.9924, F(1, 13) = 0.0001; ANOVA on LMM of volume ~ group|animal.

(F) Cumulative fentanyl consumption as function of bout size. ACCp and OFCp^{Kir2} mice drank more fentanyl than control mice and achieved the additional volume in bouts.

(G) Same as (F) for quinine. No apparent difference between the groups.

(H) ACCp and OFCp^{Kir2} mice consumed more fentanyl in bouts of 2+ rewards (ACCp and OFCp$^{Kir2.1}$ 24.5 ± 2.4 mL, control: 13.1 ± 1.2 mL, respectively; p = 0.0015, t = 3.992; t test). However, they consumed less fentanyl in single rewards (ACCp and OFCp$^{Kir2.1}$, 1.5 ± 0.4 mL; control, 4.7 ± 1.3 mL; p = 0.0312, t = 2.415; t test).

(I) No difference between the groups was evident for quinine consumption (bouts; p = 0.7660, t = 0.304; t test; Single, p = 0.1716, t = 1.447). n.s., non-significant; *p<0.05, **p<0.01; error bars and shaded errors represent SEM.

See also Figure S4 and Table S1.

consumption.[30] We therefore chose to develop a model of home-cage opioid consumption in group-housed mice in which we could assay the impact of prolonged reduction in the activity of frontal-projecting claustral neurons on fentanyl self-administration.

We developed a novel self-administration procedure in which mice are individually tagged with radio-frequency identifiers (RFIDs) and co-housed, maintaining social interactions. The home-cage of the mice is connected through a short tube to a port in which they can choose between fentanyl and quinine-adulterated water. An RFID sensor located above the tube enables continuous monitoring of the behavior of individual mice (Figure 4A). To constitutively inhibit the activity of ACCp and OFCp neurons, we stereotactically injected a retrogradely transporting AAV expressing CRE to the ACC and OFC, and AAV expressing the CRE-dependent inward rectifying potassium channel Kir2.1 to the claustrum, damping the excitability of frontal-projecting claustral neurons (Figures 4B, 4C, and S4A; as in Atlan et al.[13]). This experiment was replicated in four independent cohorts of co-housed littermate mice, balancing within each cohort between mice expressing Kir2.1 (ACCp and OFCp$^{Kir2.1}$) and control mice (ACCp and OFCp^{eGFP}).

Similar to the observations in the operant setup, mice escalated consumption in the automated cages with increasing doses of fentanyl (average daily consumption of 0.1 mg/mL fentanyl = 197 ± 9 µg, 0.15 mg/mL fentanyl = 256 ± 23 µg; Figures S4B and S4C). In this setup, ACCp and OFCp$^{Kir2.1}$ mice demonstrated increased consumption of fentanyl in comparison with controls (Figures 4D, 4F, and 4H), while no effect was observed on the consumption of quinine-adulterated water (Figures 4E, 4G, and 4I). Notably, the increased consumption of fentanyl in ACCp and OFCp$^{Kir2.1}$ mice was due to a transition toward consumption in prolonged bouts (cumulative consumption over the course of the experiment; of single fentanyl rewards,

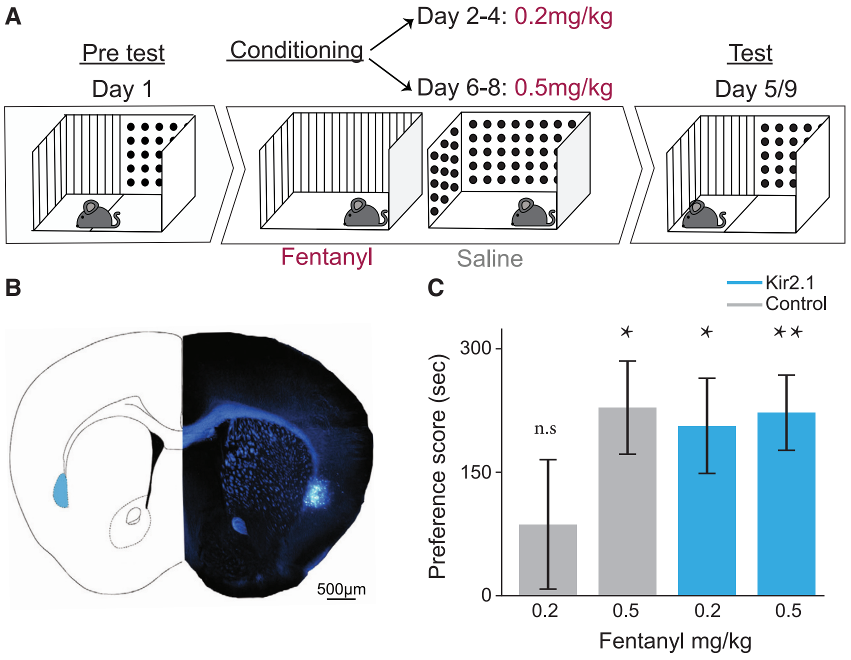

Figure 5. Chronic inhibition of frontal-projecting claustral neurons sensitizes mice to conditioned-place preference for fentanyl

(A) Experiment timeline.

(B) Representative infection. retroAAV-Cre to the ACC and OFC, and Cre-dependent Kir2.1 to the claustrum.

(C) Control (ACCp and OFCp^{GFP}) mice did not develop CPP for fentanyl at 0.2 mg/kg fentanyl, while experimental mice did (control, mean preference score 87 ± 79 s, p = 0.63, t = 1.102; paired t test; n = 7 mice; ACCp and OFCp$^{Kir2.1}$, mean preference score 189 ± 53 s, p = 0.0188, t = 3.547; paired t test, n = 8 mice). After reconditioning at 0.5 mg/kg, both groups expressed CPP for fentanyl (control, 289 ± 57 s, p = 0.0136, t = 4.04, n = 7 mice; ACCp and OFCp$^{Kir2.1}$, 247 ± 47 s, p = 0.0022, t = 5.289, n = 8 mice; paired t test). n.s., non-significant; *p < 0.05, **p < 0.01; error bars represent SEM.

See also Figure S5 and Table S1.

ACCp and OFCp$^{Kir2.1}$ 1.5 ± 0.4 mL, control 4.7 ± 1.3 mL; of bouts, ACCp and OFCp$^{Kir2.1}$ 24.5 ± 2.4 mL, control 13.1 ± 1.2 mL; Figures 4F and 4H).

Fitting geometric functions to bout length, we observed a reduction in the probability of bout termination in ACCp and OFCp$^{Kir2.1}$ mice (control [prob] = 0.49 ± 0.08; Kir2.1 [prob] = 0.26 ± 0.06; Figures S4D and S4E). No significant effect was observed on the probability to terminate bouts of quinine consumption (control [prob] = 0.51 ± 0.08; Kir2.1 [prob] = 0.37 ± 0.08; Figures S4F and S4G).

In summary, constitutive inhibition of frontal-projecting claustral neurons in group-housed mice, under conditions of unlimited daily fentanyl availability, identifies a role for claustro-frontal neurons in selectively restricting fentanyl consumption.

Chronic inhibition of frontal-projecting claustral neurons sensitizes mice to fentanyl-induced conditioned-place preference

The increased bouts of fentanyl consumption observed in mice in which claustro-frontal activity was inhibited could be potentially explained by either of two opposing hypotheses. One hypothesis is that the rewarding effect of fentanyl is reduced in claustrum-inhibited mice, and therefore they consume more drug to obtain the same effect. The opposing hypothesis would be that the rewarding effect of fentanyl is increased in claustrum-inhibited mice, causing mice to consume more due to increased drug preference. Analysis of fentanyl consumption of ACCp and OFCp$^{Kir2.1}$ versus control mice at a low dose (0.1 mg/mL, provided at FR1, FR3) versus a higher dose (0.15 mg/mL, provided at FR5) suggests that claustrum-deficient mice exhibit higher sensitivity to the drug (Figure S4H). To further differentiate between these alternative hypotheses, we performed a conditioned-place preference (CPP) experiment on a new cohort of ACCp and OFCp$^{Kir2.1}$ versus control mice. In this experiment, we first tested the preference of mice for a context paired with a borderline dose of fentanyl (0.2 mg/kg i.p.).[31] Next, we retrained the same mice on a higher fentanyl dose (0.5 mg/kg i.p.; Figures 5 and S5). We found that while control (ACCp and OFCp^{GFP}) mice did not develop CPP for fentanyl at 0.2 mg/kg (mean preference score 87 ± 79 s), ACCp and OFCp$^{Kir2.1}$ mice did (mean preference score 189 ± 53 s). After reconditioning at 0.5 mg/kg fentanyl, both groups showed fentanyl CPP (control, 289 ± 57 s; ACCp and OFCp$^{Kir2.1}$, 247 ± 47 s). These results support the hypothesis that inhibition of frontal-projecting claustral neurons increases the reinforcing effects of fentanyl.

Inhibition of frontal-projecting claustral neurons increases fentanyl-induced cortical excitation

To gain further mechanistic insight into the role of ACCp and OFCp neurons in fentanyl preference and consumption, we performed electrophysiological recordings of frontal cortical activity during exposure to fentanyl (Figures 6A and 6B). We compared activity of fronto-cortical neurons as well as subcortical neurons ("other") in control (ACCp and OFCp^{eGFP}) versus experimental (ACCp and OFCp$^{Kir2.1}$) mice (Figures 6C and S6A). Recordings were performed using Neuropixels silicon probes directed at the ACC/mPFC (Figures 6D and 6E). Mice were initially habituated to head-fixation and subcutaneous (s.c.) saline injections for 3 days. On the test day, following acute probe penetration, mice were injected with saline, followed by injection of fentanyl at the dose of 0.2 mg/kg (s.c.) that differentially induced CPP in experimental (ACCp and OFCp$^{Kir2.1}$) but not control (ACCp and OFCp^{GFP}) mice (Figure 6B).

ACCp and OFCp$^{Kir2.1}$ mice exhibited a modest shift in the distribution of baseline firing rates of fronto-cortical neurons, toward decreased firing of neurons in the ACC and infralimbic cortex (IL) (but not prelimbic cortex [PL] or OFC; Figure S6B). Fentanyl-induced bi-directional modulation of the activity of frontal units in both control and experimental mice, increasing the activity of some units while reducing the activity of others (Figures 6F, 6G, and S6C). Comparing the impact of fentanyl between experimental mice (ACCp and OFCp$^{Kir2.1}$) and controls (ACCp and OFCp^{GFP}) revealed a shift toward increased excitation in the

CellPress
OPEN ACCESS

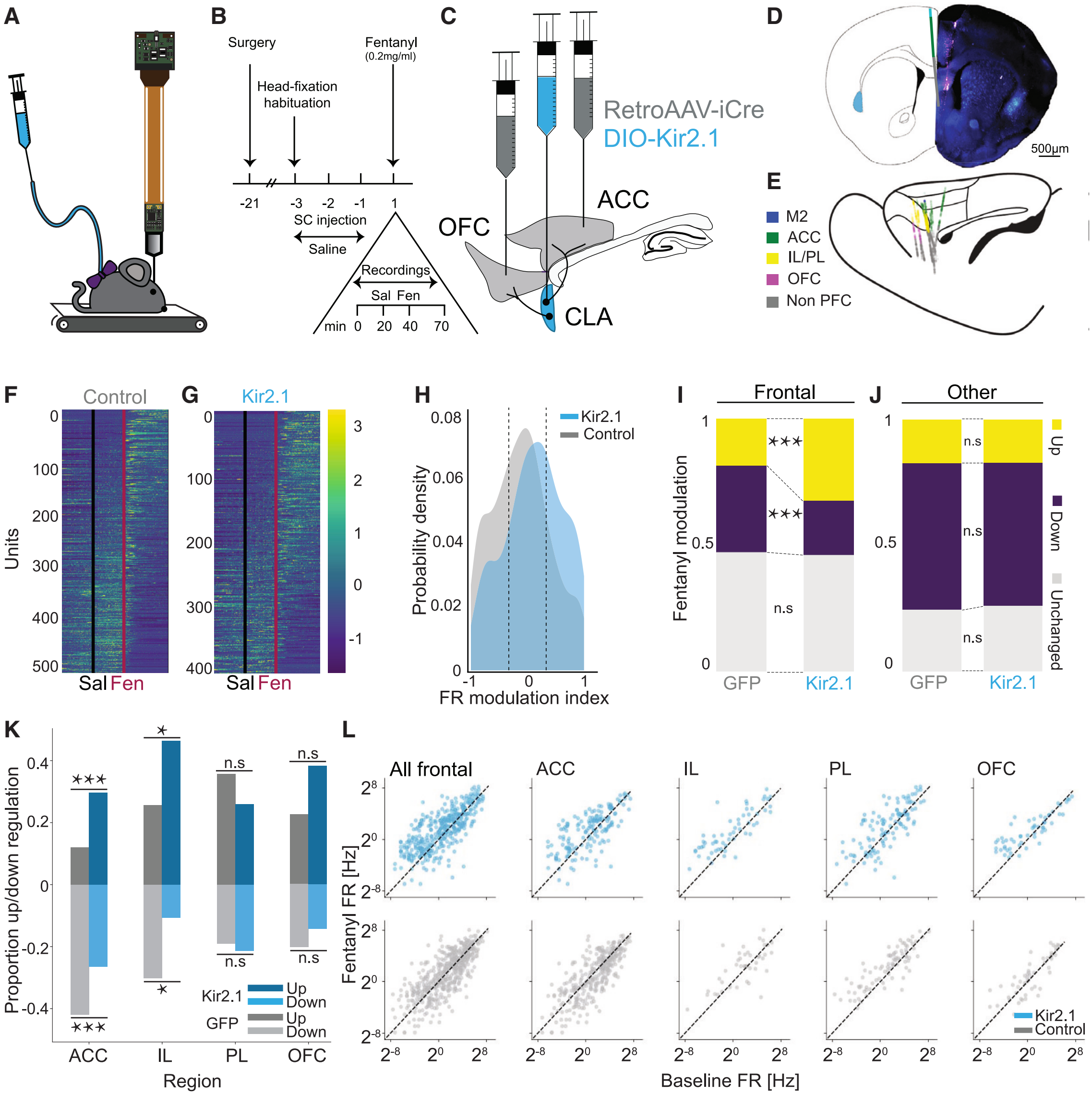

Figure 6. Fentanyl-induced cortical excitation is enhanced by inhibition of frontal-projecting claustral neurons

(A) Experimental setup. Mice are head-fixed on a linear treadmill. A neuropixels probe is inserted in the frontal cortex. Drugs are injected subcutaneously (s.c.).

(B) Experiment timeline.

(C) Viral infection scheme. retroAAV-Cre to the ACC and OFC, and Cre-dependent Kir2.1 to the claustrum.

(D) Representative infection in the claustrum and probe tracts in frontal cortex. Scale bars, 0.5 mm.

(E) Location of recorded units (n = 1,509 isolated units from 10 recordings). Sagittal view. Colors annotate brain regions.

(F and G) Heatmaps of normalized firing rates of units as a function of time. Units shown are those located in frontal cortex. Black and red lines denote the time of saline and fentanyl injection, respectively.

(F) Control mice.

(G) Experimental mice expressing Kir2.1 in frontal-projecting claustral neurons.

(H) Distribution of fentanyl modulation indices in recordings from frontal units in experimental (ACCp and OFCp^{Kir2}) versus control mice. Frontal units: control, −0.09 ± 0.02; Kir2.1, 0.08 ± 0.02; p = 2.2e−10, K-stat = 40.271; Kruskal-Wallis test.

(I and J) Proportion of units that were modulated >2-fold by fentanyl (modulation index > 0.33 or <−0.33 = marked as dashed line in H). (I) Frontal cortex versus (J) other units. Frontal units: upregulated (yellow), p = 1.6e−07, X-stat = 25.505; downregulated (purple), p = 2.2e−10, X-stat = 19.922; χ^2 test.

(K) Up/down proportions per region. ACC-down, control = 133/317, Kir2.1 = 43/162 p = 0.0009, stat = −3.31; ACC-up, control = 38/317, Kir2.1 = 48/162, p = 2e−06, stat = 4.76. IL-down, control = 13/43, Kir2.1 = 6/56, p = 0.0145, stat = −2.444; IL-up, control = 11/43, Kir2.1 = 26/56, p = 0.0336, stat = 2.125. PL-down, control = 16/84, Kir2.1 = 24/112, p = 0.6823, stat = 0.409; PL-up, control = 30/84, Kir2.1 = 29/112, p = 0.1380, stat = −1.483. OFC-down, control = 10/49, Kir2.1 = 8/55, p = 0.4302, stat = −0.789; OFC-up, control = 11/49, Kir2.1 = 21/55, p = 0.0823, stat = 1.735; χ^2 test.

(L) Firing rate (FR) correlations per region—fentanyl versus baseline. ACCp and OFCp$^{Kir2.1}$ in blue; control, ACCp, and OFCp^{GFP} in gray. n.s., non-significant; *p < 0.05, ***p < 0.001.

See also Figure S6 and Table S1.

experimental mice. The shift toward excitation was restricted to frontal units (mean fentanyl modulation indices: frontal units, control: -0.09 ± 0.02; Kir2.1, 0.08 ± 0.02; Figure 6H; other units: control, -0.19 ± 0.02; Kir2.1, -0.18 ± 0.02; Figure S6D).

The increased activation of frontal units by fentanyl during claustro-frontal inhibition was visualized by plotting the fraction of units significantly modulated by fentanyl (modulation index > 0.33 or <-0.33; Figures 6I and 6J). The impact of inhibition of claustro-frontal projections was observed most prominently in the ACC and IL (Figures 6K and 6L). In these structures, units were downregulated by fentanyl in control, yet upregulated in the experimental group. Furthermore, visualizing the dynamics of modulation, we observed a transient induction of activity in control mice in the first few minutes following injection of fentanyl, absent upon inhibition of frontal-projecting claustral neurons (Figure S6E).

Together, these results show that frontal-projecting claustral neurons restrain fentanyl-induced activation of frontal cortical neurons, and this restraint is relieved upon inhibition of ACCp and OFCp neurons. The increased stimulatory effect of fentanyl on frontal neurons observed in the presence of claustral inhibition provides a physiological correlate of the increased bout consumption of fentanyl (Figure 4) and increased sensitivity to fentanyl CPP (Figure 5) observed upon the same experimental manipulation (ACCp and OFCp$^{Kir2.1}$). These results suggest that, in the natural state, the claustrum exerts control over frontal cortical structures, restricting fentanyl sensitivity.

DISCUSSION

We investigated the role of claustrum in opioid reward, as assessed in oral drug self-administration and CPP procedures. We found that (1) neurons within the claustrum, especially frontal-projecting claustral neurons, are transcriptionally activated after non-contingent exposure to fentanyl (Figures 1 and S1), and that (2) in an oral fentanyl self-administration procedure, frontal-projecting claustral neurons (specifically ACC-projecting neurons) exhibit a unique activity pattern during bouts of fentanyl consumption (Figures 2 and S2). These activity patterns comprise a reduction in activity following delivery of the first reward in a bout. The decreased activity continues during the extent of the bout, returning to baseline only after cessation of consumption. (3) Intervening with the decrease in activity following the first fentanyl reward delivery, through optogenetic stimulation, decreased bouts of fentanyl consumption (Figures 3 and S3). (4) In contrast, constitutive inhibition of frontal-projecting claustral neurons in a group-housed self-administration procedure increased fentanyl consumption by increasing bouts of multiple fentanyl rewards (Figures 4 and S4). (5) This same manipulation also increased fentanyl CPP (Figures 5 and S5), (6) as well as the activity of frontal cortical neurons following exposure to fentanyl (Figures 6 and S6). Together, these results indicate that claustrum projections to the frontal cortex constrain the consumption of fentanyl and restrict bouts of drug intake.

In addition to the demonstration that the claustrum plays a critical role in fentanyl consumption, we also introduce a novel group-housed opioid self-administration procedure. Social interactions strongly influence drug consumption, with social isolation increasing drug consumption.[30,32] Furthermore, opioids are preferentially consumed by humans and laboratory rodents within the home environment, in contrast to psychostimulant consumption, which commonly occurs in non-home environments.[29] Most rodent models of drug intake, including recent developments,[33] have been based, due to technical considerations, on individual housing and/or consumption outside of the home-cage environment. This caveat can potentially bias observations with relation to the social interaction status of the subjects. To address this research gap, we developed a novel, ethologically relevant mouse model of opioid consumption, which is easy to implement at low cost. In this procedure, oral drug self-administration of mice is individually monitored in their home-cage, while group-housed. The availability of a simple group-housed model of opioid self-administration will support broader investigation of the role of social interactions in modulating drug consumption.

Fentanyl's impact on the claustrum

What is the mechanism whereby the claustrum is recruited by fentanyl? We addressed this question using immunohistochemistry, smFISH, and snRNA-seq. As fentanyl is primarily an MOR agonist, as well as a partial KOR agonist,[24] we expected to observe a relationship between MOR (and perhaps KOR) expression status of claustral neurons and IEG induction. However, no clear relationship was observed between MOR expression and IEG expression following fentanyl administration, while KOR expression appeared to be negatively correlated with IEG induction. We speculate that fentanyl induces IEG expression in the claustrum through an indirect mechanism, potentially through excitation of cortico-claustral projections that are enriched for synaptic inputs onto the IEG-expressing claustral neurons. An alternative indirect mechanism could be mediated by the intermediate release of a neuromodulator, such as dopamine or serotonin,[34,35] which then acts on specific populations of claustral neurons. Elucidating the circuitry mediating fentanyl recruitment of claustral neurons, as well as the roles of MOR and KOR expression in the claustrum, are topics for future investigation.

Do the shifts in Ca^{2+} activity of ACCp and OFCp neurons during fentanyl oral self-administration inform us of the mechanism of action of fentanyl on the claustrum? We interpret the Ca^{2+} signals as corresponding to the commitment to bout consumption rather than reflecting the pharmacological impact of fentanyl on claustral neurons, as the time delay from oral fentanyl consumption to CNS impact is presumed to be on the order of minutes.[36] The elevated Ca^{2+} signal prior to the first delivery of a fentanyl reward in a bout likely corresponds to the series of licks the mouse is required to implement (in an FR5 schedule) prior to delivery of the first reward. Lick-associated transients have been previously observed for ACCp and OFCp neurons,[17] as well as for other claustral neurons.[21] We interpret the dip in Ca^{2+} following the delivery of the first reward as a response to the delivery of the reward and a commitment to a prolonged bout of consumption rather than a reflection of the pharmacological impact of fentanyl. The results of optogenetic intervention with bout consumption are consistent with this interpretation.

Claustro-frontal regulation of opioid consumption

How could claustro-frontal projections elicit control over opioid consumption? Most research on the mechanisms underlying opioid reward have focused on the mesolimbic dopamine system, identifying the central contributions of midbrain dopamine neurons and nucleus accumbens opiate receptors.[37–40]

CellPress
OPEN ACCESS

Afferents of midbrain dopamine neurons are found within the claustrum, and claustral neurons (especially frontal-projecting claustral neurons) express dopamine receptors.[13,41–44] The claustrum may therefore be viewed as part of the extended reward circuitry, providing context to its function in modulating opioid reinforcement.[44,45]

Our results indicate that the function of the claustrum in fentanyl reward is mediated by its impact on the frontal cortex. Regions of the frontal cortex have been implicated in different aspects of value-risk assessment, decision making, and impulse control, and as key regulators of limbic reward regions.[46–51] Specifically, the ACC has been implicated in reward processing and incentive salience.[52] Therefore, we speculate that the impact of the claustrum on the cortex relates to cognitive control. Impairment in cognitive functions, such as impulse control, are strongly linked to drug addiction, both as a risk factor and a consequence of drug consumption.[53–56] Moreover, impairments in cognitive control may predispose individuals to first use of an illicit drug and the transition from recreational to heavy drug use.[57] Several recent publications describe the capacity of claustro-frontal projection neurons to elicit inhibitory control via feed-forward inhibition of frontal pyramidal neurons,[15,58,59] as well as inhibition of cortical sensory responses.[13] Our observations of claustral inhibition of frontal excitation by fentanyl is further consistent with inhibitory control elicited by the claustrum over the frontal cortex.

Together, these studies suggest that the claustrum restricts the activity of the frontal cortex, such that reduced activity of this claustral projection releases the brake on the frontal cortex, diminishing behavioral inhibition. Consistent with this notion is our recent observation whereby low activity of ACC-projecting claustral neurons is associated with disinhibition of behavior and increased impulsivity.[17] Also consistent are the results reported herein of a prolonged dip in ACCp activity during bouts of fentanyl consumption and termination of bouts by optogenetic intervention. Loss of frontal control could lead to increased opioid consumption and, in humans, could potentially contribute to accelerating the path to drug addiction.

Conclusions

We found that frontal-projecting claustral neurons decreased their activity during the initiation of bouts of fentanyl consumption. Prolonged inhibition of these neurons selectively increased fentanyl bout consumption. This manipulation also increased excitation of frontal-cortical structures by fentanyl, as well as fentanyl CPP. Together, these results indicate that the claustrum normally exerts inhibitory control over frontal cortical neurons that play a key role in opioid reward.

STAR★METHODS

Detailed methods are provided in the online version of this paper and include the following:

- KEY RESOURCES TABLE
- RESOURCE AVAILABILITY
  - Lead contact
  - Materials availability
  - Data and code availability
- EXPERIMENTAL MODEL AND SUBJECT DETAILS
  - Animals
- METHOD DETAILS
  - Drugs
  - Surgery
  - Single molecule fluorescence in-situ hybridization (smFISH)
  - Single nuclei RNA sequencing (snRNAseq)
  - Immunohistochemistry
  - Fiber Photometry during Self-Administration of Fentanyl vs Quinine
  - Optogenetic stimulation during oral self-administration
  - Automated Cages Oral Self-Administration of Fentanyl
  - Apparatus
  - Behavioral Procedure
  - Intermittent Access
  - Quinine Adulteration
  - Conditioned Place Preference
  - In-vivo Electrophysiology Recording
  - Geometric fit to bout length
- QUANTIFICATION AND STATISTICAL ANALYSIS
  - Figures

SUPPLEMENTAL INFORMATION

Supplemental information can be found online at https://doi.org/10.1016/j.cub.2023.05.065.

ACKNOWLEDGMENTS

The authors appreciate the helpful comments of Drs. Yavin Shaham, Robert Malenka, and Inbal Goshen on experimental design, data, writing, and presentation. We are indebted to Dr. Nadav Yayon, former head of the ELSC Core Microscopy Unit (ECMU), for technical support in establishing the imaging system required for efficient acquisition of multiplexed smFISH signals. We thank Dr. Masha Adam for technical assistance with the single-nucleus RNA sequencing. We are appreciative of the efforts of Dr. Maya Sherman, head of the viral vector core facility, in striving to optimize viral preparations. We thank Ms. Idit Yvgi for helping us with aesthetic graphics for the manuscript. This work was supported by grants from the European Research Council (grant ERC 770951), the Israel Science Foundation (grants 1062/18, 393/12, 1796/12, and 2341/15), the Israel Anti-Drug Administration, an Adelis Award for Advances in Neuroscience, contributions from anonymous philanthropists in Los Angeles and Mexico City, as well as research support from the Safra Center for Brain Sciences (ELSC) and the Canadian Institute for Advanced Research (CIFAR).

This work is dedicated to the memory of Mrs. Lily Safra, a great supporter of brain research.

AUTHOR CONTRIBUTIONS

Study concept and design, A.T., Y.F., and A.C.; smFISH, A.T.; single-cell sequencing, H.T., D.K., Y.F., and N.H.; IHC, A.T.; fiber photometry, Y.F. and A.T.; optogenetics, Y.F. and A.T.; automated cages, Y.F. and A.T.; CPP, A.T. and Y.F.; NPXL, N.P.-R., Y.F., and A.T.; data analysis, Y.F., S.S.K., R.A.-F., N.P.-R., and A.T.; initial investigations and calibrations, D.M. and H.T.; drafting of the manuscript, A.T., Y.F., and A.C.; obtained funding, A.C.; study supervision, A.C.

DECLARATION OF INTERESTS

The authors declare no competing interests.

INCLUSION AND DIVERSITY

We support inclusive, diverse, and equitable conduct of research.

Received: October 20, 2022
Revised: April 13, 2023
Accepted: May 26, 2023
Published: June 26, 2023

REFERENCES

1. Florence, C., Luo, F., and Rice, K. (2021). The economic burden of opioid use disorder and fatal opioid overdose in the United States, 2017. Drug Alcohol Depend. *218*, 108350. https://doi.org/10.1016/j.drugalcdep.2020.108350.
2. Kosten, T.R., and Petrakis, I.L. (2021). The hidden epidemic of opioid overdoses during the coronavirus disease 2019 pandemic. JAMA Psychiatry *78*, 585–586. https://doi.org/10.1001/jamapsychiatry.2020.4148.
3. Keefe, P.R. (2021). Empire of Pain: The Secret History of the Sackler Dynasty (Doubleday Publishing).
4. Firestone, L.L., Gyulai, F., Mintun, M., Adler, L.J., Urso, K., and Winter, P.M. (1996). Human brain activity response to fentanyl imaged by positron emission tomography. Anesth. Analg. *82*, 1247–1251. https://doi.org/10.1097/00000539-199606000-00025.
5. Leppä, M., Korvenoja, A., Carlson, S., Timonen, P., Martinkauppi, S., Ahonen, J., Rosenberg, P.H., Aronen, H.J., and Kalso, E. (2006). Acute opioid effects on human brain as revealed by functional magnetic resonance imaging. Neuroimage *31*, 661–669. https://doi.org/10.1016/j.neuroimage.2005.12.019.
6. Chen, C., Willhouse, A.H., Huang, P., Ko, N., Wang, Y., Xu, B., Huang, L.H.M., Kieffer, B., Barbe, M.F., and Liu-Chen, L.Y. (2020). Characterization of a knock-in mouse line expressing a fusion protein of κ opioid receptor conjugated with tdTomato: 3-dimensional brain imaging via CLARITY. eNeuro *7*, https://doi.org/10.1523/ENEURO.0028-20.2020.
7. Wang, Y.J., Rasakham, K., Huang, P., Chudnovskaya, D., Cowan, A., and Liu-Chen, L.Y. (2011). Sex difference in κ-opioid receptor (KOPR)-mediated behaviors, brain region KOPR level and KOPR-mediated guanosine 5′-O-(3-[35S]thiotriphosphate) binding in the guinea pig. J. Pharmacol. Exp. Ther. *339*, 438–450. https://doi.org/10.1124/jpet.111.183905.
8. Lutz, P.E., and Kieffer, B.L. (2013). The multiple facets of opioid receptor function: implications for addiction. Curr. Opin. Neurobiol. *23*, 473–479. https://doi.org/10.1016/j.conb.2013.02.005.
9. Bruchas, M.R., Land, B.B., and Chavkin, C. (2010). The dynorphin/kappa opioid system as a modulator of stress-induced and pro-addictive behaviors. Brain Res. *1314*, 44–55. https://doi.org/10.1016/j.brainres.2009.08.062.
10. Wee, S., and Koob, G.F. (2010). The role of the dynorphin-κ opioid system in the reinforcing effects of drugs of abuse. Psychopharmacol. (Berl.) *210*, 121–135. https://doi.org/10.1007/s00213-010-1825-8.
11. Mantsch, J.R., Baker, D.A., Funk, D., Lê, A.D., and Shaham, Y. (2016). Stress-induced reinstatement of drug seeking: 20 years of progress. Neuropsychopharmacology *41*, 335–356. https://doi.org/10.1038/npp.2015.142.
12. Brynildsen, J.K., Mace, K.D., Cornblath, E.J., Weidler, C., Pasqualetti, F., Bassett, D.S., and Blendy, J.A. (2020). Gene coexpression patterns predict opiate-induced brain-state transitions. Proc. Natl. Acad. Sci. USA *117*, 19556–19565. https://doi.org/10.1073/pnas.2003601117.
13. Atlan, G., Terem, A., Peretz-Rivlin, N., Mizrahi, A., Nelken, I., Citri, A., Goll, Y., Refaeli, R., Zviran, O., Lim, B.K., et al. (2018). The claustrum supports resilience to distraction article the claustrum supports resilience to distraction current biology. Curr. Biol. *28*, 2752–2762.e7. https://doi.org/10.1016/j.cub.2018.06.068.
14. Jackson, J., Karnani, M.M., Zemelman, B.V., Burdakov, D., and Lee, A.K. (2018). Inhibitory control of prefrontal cortex by the claustrum. Neuron *99*, 1029–1039.e4. https://doi.org/10.1016/j.neuron.2018.07.031.
15. Narikiyo, K., Mizuguchi, R., Ajima, A., Shiozaki, M., Hamanaka, H., Johansen, J.P., Mori, K., and Yoshihara, Y. (2020). The claustrum coordinates cortical slow-wave activity. Nat. Neurosci. *23*, 741–753. https://doi.org/10.1038/s41593-020-0625-7.
16. Chia, Z., Augustine, G.J., and Silberberg, G. (2020). Synaptic connectivity between the cortex and claustrum is organized into functional modules. Curr. Biol. *30*, 2777–2790.e4. https://doi.org/10.1016/j.cub.2020.05.031.
17. Atlan, G., Matosevich, N., Peretz-Rivlin, N., Yvgi, I., Chen, E., Kleinman, T., Bleistein, N., Sheinbach, E., Groysman, M., Nir, Y., et al. (2021). Claustral projections to anterior cingulate cortex modulate engagement with the external world. Preprint at bioRxiv. https://doi.org/10.1101/2021.06.17.448649.
18. Liu, J., Wu, R., Johnson, B., Vu, J., Bass, C., and Li, J.X. (2019). The claustrum-prefrontal cortex pathway regulates impulsive-like behavior. J. Neurosci. *39*, 10071–10080. https://doi.org/10.1523/JNEUROSCI.1005-19.2019.
19. White, M.G., Panicker, M., Mu, C., Carter, A.M., Roberts, B.M., Dharmasri, P.A., and Mathur, B.N. (2018). Anterior cingulate cortex input to the claustrum is required for top-down action control. Cell Rep. *22*, 84–95. https://doi.org/10.1016/j.celrep.2017.12.023.
20. Fodoulian, L., Gschwend, O., Huber, C., Mutel, S., Leone, R., Renfer, J.-R., Ekundayo, K., Rodriguez, I., and Carleton, A. (2020). The claustrum-medial prefrontal cortex network controls attentional set-shifting. Preprint at bioRxiv. https://doi.org/10.1101/2020.10.14.339259.
21. Chevée, M., Finkel, E.A., Kim, S.J., O'Connor, D.H., and Brown, S.P. (2022). Neural activity in the mouse claustrum in a cross-modal sensory selection task. Neuron *110*, 486–501.e7. https://doi.org/10.1016/j.neuron.2021.11.013.
22. Fujii, K., Koshidaka, Y., Adachi, M., and Takao, K. (2019). Effects of chronic fentanyl administration on behavioral characteristics of mice. Neuropsychopharmacol. Rep. *39*, 17–35. https://doi.org/10.1002/npr2.12040.
23. Habib, N., Avraham-Davidi, I., Basu, A., Burks, T., Shekhar, K., Hofree, M., Choudhury, S.R., Aguet, F., Gelfand, E., Ardlie, K., et al. (2017). Massively parallel single-nucleus RNA-seq with DroNc-seq. Nat. Methods *14*, 955–958. https://doi.org/10.1038/nmeth.4407.
24. Comer, S.D., and Cahill, C.M. (2019). Fentanyl: receptor pharmacology, abuse potential, and implications for treatment. Neurosci. Biobehav. Rev. *106*, 49–57. https://doi.org/10.1016/j.neubiorev.2018.12.005.
25. El Rawas, R., Thiriet, N., Lardeux, V., Jaber, M., and Solinas, M. (2009). Environmental enrichment decreases the rewarding but not the activating effects of heroin. Psychopharmacol. (Berl.) *203*, 561–570. https://doi.org/10.1007/s00213-008-1402-6.
26. ZióŁkowska, B., Gieryk, A., Solecki, W., and PrzewŁocki, R. (2015). Temporal and anatomic patterns of immediate-early gene expression in the forebrain of C57BL/6 and DBA/2 mice after morphine administration. Neuroscience *284*, 107–124. https://doi.org/10.1016/j.neuroscience.2014.09.069.
27. Reiner, D.J., Lofaro, O.M., Applebey, S.V., Korah, H., Venniro, M., Cifani, C., Bossert, J.M., and Shaham, Y. (2020). Role of projections between piriform cortex and orbitofrontal cortex in relapse to fentanyl seeking after palatable food choice-induced voluntary abstinence. J. Neurosci. *40*, 2485–2497. https://doi.org/10.1523/JNEUROSCI.2693-19.2020.
28. Monroe, S.C., and Radke, A.K. (2021). Aversion-resistant fentanyl self-administration in mice. Psychopharmacology (Berl) *238*, 699–710.
29. Badiani, A., Belin, D., Epstein, D., Calu, D., and Shaham, Y. (2011). Opiate versus psychostimulant addiction: the differences do matter. Nat. Rev. Neurosci. *12*, 685–700. https://doi.org/10.1038/nrn3104.
30. Heilig, M., Epstein, D.H., Nader, M.A., and Shaham, Y. (2016). Time to connect: bringing social context into addiction neuroscience. Nat. Rev. Neurosci. *17*, 592–599. https://doi.org/10.1038/nrn.2016.67.
31. Anand, J.P., Boyer, B.T., Mosberg, H.I., and Jutkiewicz, E.M. (2016). The behavioral effects of a mixed efficacy antinociceptive peptide, VRP26, following chronic administration in mice. Psychopharmacol. (Berl.) *233*, 2479–2487. https://doi.org/10.1007/s00213-016-4296-8.
32. Venniro, M., Russell, T.I., Ramsey, L.A., Richie, C.T., Lesscher, H.M.B., Giovanetti, S.M., Messing, R.O., and Shaham, Y. (2020). Abstinence-dependent dissociable central amygdala microcircuits control drug craving. Proc. Natl. Acad. Sci. USA *117*, 8126–8134. https://doi.org/10.1073/pnas.2001615117.

CellPress
OPEN ACCESS

33. Moussawi, K., Ortiz, M.M., Gantz, S.C., Tunstall, B.J., Marchette, R.C.N., Bonci, A., Koob, G.F., and Vendruscolo, L.F. (2020). Fentanyl vapor self-administration model in mice to study opioid addiction. Sci. Adv. *6*. eabc0413. https://doi.org/10.1126/sciadv.abc0413.

34. Sustkova-Fiserova, M., Puskina, N., Havlickova, T., Lapka, M., Syslova, K., Pohorala, V., and Charalambous, C. (2020). Ghrelin receptor antagonism of fentanyl-induced conditioned place preference, intravenous self-administration, and dopamine release in the nucleus accumbens in rats. Addict. Biol. *25*. e12845. https://doi.org/10.1111/adb.12845.

35. Baldo, B.A., and Rose, M.A. (2020). The anaesthetist, opioid analgesic drugs, and serotonin toxicity: a mechanistic and clinical review. Br. J. Anaesth. *124*, 44–62. https://doi.org/10.1016/j.bja.2019.08.010.

36. Scott, J.C., Ponganis, K.V., and Stanski, D.R. (1985). EEG quantitation of narcotic effect: the comparative pharmacodynamics of fentanyl and alfentanil. Anesthesiology *62*, 234–241. https://doi.org/10.1097/00000542-198503000-00005.

37. Corre, J., van Zessen, R., Loureiro, M., Patriarchi, T., Tian, L., Pascoli, V., and Lüscher, C. (2018). Dopamine neurons projecting to medial shell of the nucleus accumbens drive heroin reinforcement. eLife *7*. e39945. https://doi.org/10.7554/eLife.39945.

38. Galaj, E., Han, X., Shen, H., Jordan, C.J., He, Y., Humburg, B., Bi, G.H., and Xi, Z.X. (2020). Dissecting the role of GABA neurons in the VTA versus SNr in opioid reward. J. Neurosci. *40*, 8853–8869. https://doi.org/10.1523/JNEUROSCI.0988-20.2020.

39. Stinus, L., Cador, M., and Le Moal, M. (1992). Interaction between endogenous opioids and dopamine within the nucleus accumbens. Ann. N. Y. Acad. Sci. *654*, 254–273. https://doi.org/10.1111/j.1749-6632.1992.tb25972.x.

40. Vaccarino, F.J., Bloom, F.E., and Koob, G.F. (1985). Blockade of nucleus accumbens opiate receptors attenuates intravenous heroin reward in the rat. Psychopharmacol. (Berl.) *86*, 37–42. https://doi.org/10.1007/BF00431681.

41. Baizer, J.S., Sherwood, C.C., Noonan, M., and Hof, P.R. (2014). Comparative organization of the claustrum: what does structure tell us about function? Front. Syst. Neurosci. *8*, 117. https://doi.org/10.3389/fnsys.2014.00117.

42. Terem, A., Gonzales, B.J., Peretz-Rivlin, N., Ashwal-Fluss, R., Bleistein, N., del Mar Reus-Garcia, M., Mukherjee, D., Groysman, M., and Citri, A. (2020). Claustral neurons projecting to frontal cortex mediate contextual association of reward. Curr. Biol. *30*, 3522–3532.e6. https://doi.org/10.1016/j.cub.2020.06.064.

43. Zingg, B., Dong, H.W., Tao, H.W., and Zhang, L.I. (2018). Input–output organization of the mouse claustrum. J. Comp. Neurol. *526*, 2428–2443. https://doi.org/10.1002/cne.24502.

44. Wong, K.L.L., Nair, A., and Augustine, G.J. (2021). Changing the cortical conductor's tempo: neuromodulation of the claustrum. Front. Neural Circuits *15*, 658228. https://doi.org/10.3389/fncir.2021.658228.

45. Graf, M., Wong, K.L.L., and Augustine, G.J. (2020). Neuroscience: a role for the claustrum in drug reward. Curr. Biol. *30*. R1038–R1040. https://doi.org/10.1016/j.cub.2020.07.031.

46. Goldstein, R.Z., and Volkow, N.D. (2011). Dysfunction of the prefrontal cortex in addiction: neuroimaging findings and clinical implications. Nat. Rev. Neurosci. *12*, 652–669. https://doi.org/10.1038/nrn3119.

47. Niendam, T.A., Laird, A.R., Ray, K.L., Dean, Y.M., Glahn, D.C., and Carter, C.S. (2012). Meta-analytic evidence for a superordinate cognitive control network subserving diverse executive functions. Cogn. Affect. Behav. Neurosci. *12*, 241–268. https://doi.org/10.3758/s13415-011-0083-5.

48. Ott, T., and Nieder, A. (2019). Dopamine and cognitive control in prefrontal cortex. Trends Cogn. Sci. *23*, 213–234. https://doi.org/10.1016/j.tics.2018.12.006.

49. Widge, A.S., Zorowitz, S., Basu, I., Paulk, A.C., Cash, S.S., Eskandar, E.N., Deckersbach, T., Miller, E.K., and Dougherty, D.D. (2019). Deep brain stimulation of the internal capsule enhances human cognitive control and prefrontal cortex function. Nat. Commun. *10*, 1536. https://doi.org/10.1038/s41467-019-09557-4.

50. van Steenbergen, H., Eikemo, M., and Leknes, S. (2019). The role of the opioid system in decision making and cognitive control: a review. Cogn. Affect. Behav. Neurosci. *19*, 435–458. https://doi.org/10.3758/s13415-019-00710-6.

51. Zhao, Y., Sallie, S.N., Cui, H., Zeng, N., Du, J., Yuan, T., Li, D., De Ridder, D., and Zhang, C. (2020). Anterior cingulate cortex in addiction: new insights for neuromodulation. Neuromodulation *24*, 187–196. https://doi.org/10.1111/ner.13291.

52. Volkow, N.D., and Baler, R.D. (2013). Brain imaging biomarkers to predict relapse in alcohol addiction. JAMA Psychiatry *70*, 661–663. https://doi.org/10.1001/jamapsychiatry.2013.1141.

53. Argyriou, E., Um, M., Carron, C., and Cyders, M.A. (2018). Age and impulsive behavior in drug addiction: a review of past research and future directions. Pharmacol. Biochem. Behav. *164*, 106–117. https://doi.org/10.1016/J.PBB.2017.07.013.

54. Dalley, J.W., and Ersche, K.D. (2019). Neural circuitry and mechanisms of waiting impulsivity: relevance to addiction. Philos. Trans. R. Soc. Lond. B Biol. Sci. *374*, 20180145. https://doi.org/10.1098/RSTB.2018.0145.

55. Jentsch, J.D., Ashenhurst, J.R., Cervantes, M.C., James, A.S., Groman, S.M., and Pennington, Z.T. (2014). Dissecting impulsivity and its relationships to drug addictions. Ann. N. Y. Acad. Sci. *1*, 1327. https://doi.org/10.1111/NYAS.12388.

56. Sanchez-Roige, S., Baro, V., Trick, L., Peña-Oliver, Y., Stephens, D.N., and Duka, T. (2014). Exaggerated waiting impulsivity associated with human binge drinking, and high alcohol consumption in mice. Neuropsychopharmacol. *3913*, 2919–2927. https://doi.org/10.1038/npp.2014.151.

57. Garavan, H., and Hester, R. (2007). The role of cognitive control in cocaine dependence. Neuropsychol. Rev. *17*, 337–345. https://doi.org/10.1007/s11065-007-9034-x.

58. Jackson, J. (2018). Attention: noisy networks are tuned out by the claustrum. Curr. Biol. *28*. R937–R939. https://doi.org/10.1016/j.cub.2018.07.031.

59. Wang, Q., Wang, Y., Xie, P., Kuo, H.-C., Hirokawa, K.E., Naeemi, M., Yao, S., Ouellette, B., Mallory, M., Lesnar, P., et al. (2022). Regional and cell type-specific afferent and efferent projections of the mouse claustrum. Cell Rep. *42*, 112118.

60. Schindelin, J., Arganda-Carreras, I., Frise, E., Kaynig, V., Longair, M., Pietzsch, T., Preibisch, S., Rueden, C., Saalfeld, S., Schmid, B., et al. (2012). Fiji: an open-source platform for biological-image analysis. Nat. Methods *9*, 676–682. https://doi.org/10.1038/nmeth.2019.

61. Gonzales, B.J., Mukherjee, D., Ashwal-Fluss, R., Loewenstein, Y., and Citri, A. (2019). Subregion-specific rules govern the distribution of neuronal immediate-early gene induction. Proc. Natl. Acad. Sci. USA *117*, 23304–23310. https://doi.org/10.1073/pnas.1913658116.

62. Paxinos, G., and Franklin, K.B.J. (2013). The Mouse Brain in Stereotaxic Coordinates (Academic Press).

63. Kamentsky, L., Jones, T.R., Fraser, A., Bray, M.A., Logan, D.J., Madden, K.L., Ljosa, V., Rueden, C., Eliceiri, K.W., and Carpenter, A.E. (2011). Improved structure, function and compatibility for CellProfiler: modular high-throughput image analysis software. Bioinformatics *27*, 1179–1180. https://doi.org/10.1093/bioinformatics/btr095.

64. Lopes, G., Bonacchi, N., Frazão, J., Neto, J.P., Atallah, B.V., Soares, S., Moreira, L., Matias, S., Itskov, P.M., Correia, P.A., et al. (2015). Bonsai: an event-based framework for processing and controlling data streams. Front. Neuroinform. *9*, 7. https://doi.org/10.3389/fninf.2015.00007.

65. Feigin, L., Tasaka, G., Maor, I., and Mizrahi, A. (2021). Sparse coding in temporal association cortex improves complex sound discriminability. J. Neurosci. *41*, 7048–7064. https://doi.org/10.1523/JNEUROSCI.3167-20.2021.

66. Pachitariu, M., Steinmetz, N., Kadir, S., Carandini, M., and Harris, K.D. (2016). Kilosort: realtime spike-sorting for extracellular electrophysiology with hundreds of channels. Preprint at bioRxiv. https://doi.org/10.1101/061481.

STAR★METHODS

KEY RESOURCES TABLE

REAGENT or RESOURCE	SOURCE	IDENTIFIER
Antibodies		
Rabbit anti-Fos	Synaptic Systems	RRID: AB_2231974
donkey anti-rabbit IgG H&L Alexa Fluor 488	Abcam	RRID: AB_2860569
Bacterial and virus strains		
AAVdj-DIO-eGFP	Vector core facility of the Edmond and Lily Safra Center for Brain Sciences	N/A
AAVdj-EF1a-DIO-Kir2.1-t2A-zsGreen	Stanford viral core facility	N/A
AAV9-DIO-GCaMP6s	Vector core facility of the Edmond and Lily Safra Center for Brain Sciences	N/A
retroAAV-CKII-iCre	Vector core facility of the Edmond and Lily Safra Center for Brain Sciences	N/A
retroAAV-H2B-eGFP	Vector core facility of the Edmond and Lily Safra Center for Brain Sciences	N/A
retroAAV-H2B-Ruby	Vector core facility of the Edmond and Lily Safra Center for Brain Sciences	N/A
AAV9-CAGGS-FLEX-ChR2-TdTomato	UPENN virus core facility	AV-9-18917P; RRID: Addgene_18917
Chemicals, peptides, and recombinant proteins		
Fentanyl, Fenta injections	Shaare Zedek Hospital, Jerusalem	N/A
Deposited data		
Mendeley dataset	N/A	https://doi.org/10.17632/9nrrwz2m9p.1
Experimental models: Organisms/strains		
C57BL/6	Harlan Laboratories	C57BL/6JOLAHSD MICE; RRID: IMSR_ENV:HSD-057
Oligonucleotides		
RNAscope probe: Mm-Egr2	Advanced Cell Diagnostics	Cat No. 407871
RNAscope probe: Mm-Fos-C3	Advanced Cell Diagnostics	Cat No. 316921-C3
Software and algorithms		
R	R version 4.2.0	https://www.r-project.org; RRID: SCR_001905
Photoshop, Illustrator, and InDesign	Adobe, San Jose, CA	https://www.adobe.com/products/illustrator.html; RRID: SCR_014199, RRID: SCR_010279, RRID: SCR_021799
CellProfiler	Broad Institute	https://cellprofiler.org/; RRID: SCR_007358
cellRanger	Cell Ranger 7.0.0	https://support.10xgenomics.com/single-cell-vdj/software/pipelines/latest/what-is-cell-ranger; RRID: SCR_017344
MATLAB-based state machine	Bpod, Sanworks	https://sanworks.io/index.php; RRID: SCR_015943

(*Continued on next page*)

Continued

REAGENT or RESOURCE	SOURCE	IDENTIFIER
Neurophotometrics FP3002	Neurophotometrics LTD, San Diego, CA	https://neurophotometrics.com/
FIJI	IMAGE-J[60]	RRID: SCR_002285

RESOURCE AVAILABILITY

Lead contact

Further information and requests for resources and reagents should be directed to and will be fulfilled by the lead contact, Dr. Ami Citri (ami.citri@mail.huji.ac.il).

Materials availability

This study did not generate new unique reagents.

Data and code availability

The datasets generated and/or analyzed in the current study are available from the lead contact upon reasonable request. This paper does not report original code. Any additional information required to reanalyze the data reported in this paper is available from the lead contact upon request.

EXPERIMENTAL MODEL AND SUBJECT DETAILS

Animals

Mice described in this study were C57BL/6 inbred male mice purchased from Harlan Laboratories, Jerusalem. All mice were maintained on a 12-hour light-dark cycle in a specific pathogen-free (SPF) animal facility with free access to food. Ad-libitum water was provided when possible, with the exception of oral self-administration experiments. All experimental procedures, handling, surgeries and care of laboratory animals used in this study were approved by the Hebrew University Institutional Animal Care and Use Committee (IACUC). A total of 89 mice were used in this study, according to the specifications in the table below.

Experiment	# of animals	Viral Constructs	Location and Volumes
Oral Self Administration of Fentanyl, Home Cage	Control n=7 Kir2.1 n=8	retroAAV-CKII-iCre<br>AAVdj-CAG-DIO-GPE2 (Kir2.1)-szGreen OR AAVdj-DIO-eGFP	ACC: ±0.25, 1.1, -1.75, 200nl<br>OFC: ±1, 2.55, -2.4, 200nl<br>CLA: ±2.8, 1, -3.7;<br>±3.25, 0, -4.15, 200nl
Fentanyl CPP	Control n=7 Kir2.1 n=8	retroAAV-CKII-iCre<br>AAVdj-CAG-DIO-GPE2 (Kir2.1)-szGreen OR AAVdj-DIO-eGFP	ACC: ±0.25, 1.1, -1.75, 200nl<br>OFC: ±1, 2.55, -2.4, 200nl<br>CLA: ±2.8, 1, -3.7;<br>±3.25, 0, -4.15, 200nl
Photometry recording during fentanyl self-administration	ACCp n=5	retroAAV-CKII-iCre<br>AAV9-DIO-GCaMP6s	ACC: ±0.25, 1.1, -1.75, 250nl<br>CLA: ±2.8, 1, -3.7;<br>±3.25, 0, -4.15, 250nl<br>Cannulas positioned at ±2.8, 1, -3.55
Photometry – OFCp	OFCp n=6<br>1 mouse excluded from signal analysis (no signal)	retroAAV-CKII-iCre<br>AAV9-DIO-GCaMP6s	OFC: ±1, 2.55, -2.4, 250nl<br>CLA: ±2.8, 1, -3.7;<br>±3.25, 0, -4.15, 250nl<br>Cannulas positioned at ±2.8, 1, -3.55
Optogenetics	Control n=5<br>ChR2 n=5	retroAAV-CKII-iCre<br>AAV9-CAGGS-FLEX-ChR2-TdTomato<br>AAVdj-DIO-eGFP	ACC: ±0.25, 1.1, -1.75, 200nl<br>OFC: ±1, 2.55, -2.4, 200nl<br>CLA: ±2.8, 1, -3.7;<br>±3.25, 0, -4.15, 200nl<br>Cannulas positioned at ±2.8, 1, -3.55
In-vivo electrophysiology recordings (NPXL)	GFP n=4<br>Kir2.1 n=5	retroAAV-CKII-iCre<br>AAVdj-CAG-DIO-GPE2 (Kir2.1)-szGreen OR AAVdj-DIO-eGFP	ACC: ±0.25, 1.1, -1.75, 200nl<br>OFC: ±1, 2.55, -2.4, 200nl<br>CLA: ±2.8, 1, -3.7;<br>±3.25, 0, -4.15, 200nl

(*Continued on next page*)

Continued

Experiment	# of animals	Viral Constructs	Location and Volumes
IEG expression in the CLA after acute fentanyl - IHC	saline n=5 fentanyl n=6	retroAAV-H2B-eGFP<br>retroAAV-H2B-Ruby	ACC: ±0.25, 1.1, -1.75, 200nl<br>OFC: ±1, 2.55, -2.4, 200nl
smFISH	Saline n=2 fentanyl n=2	No virus	
snRNAseq ± fentanyl	saline n=7 fentanyl n=7	retroAAV-eGFP<br>retroAAV-Ruby	ACC: ±0.25, 1.1, -1.75, 200nl
Total	N=89	Comments: (1) Coordinates are denoted as Lateral-Medial, Anterior-Posterior, Dorsal-Ventral in mm from Bregma.<br>(2) injections were performed bilaterally, symmetrically; where noted, two injections were performed in each hemisphere.	

METHOD DETAILS

Drugs

Fentanyl (Fenta injections, 0.05 mg/ml), was diluted in saline and injected *ip* or *sc*. fentanyl for oral consumption was obtained from Shaare Zedek Hospital, Jerusalem, in a stock concentration of 4 mg/ml, and diluted to 0.1 mg/ml or 0.15 mg/ml in mice acidic water.

Surgery

Induction and maintenance of anesthesia during surgery was achieved using SomnoSuite Low-Flow Anesthesia System (Kent Scientific Corporation). Following induction of anesthesia, animals were quickly secured to the stereotaxic apparatus (David KOPF instruments). Anesthesia depth was validated by toe-pinching and isoflurane level were adjusted (1-5%) to maintain relaxed breathing. The skin was cleaned with Betadine (Dr. Fischer Medical), Lidocaine (Rafa Laboratories) was applied to minimize pain, and Viscotear gel was applied to protect the eyes. An incision was made to expose the skull, which was immediately cleaned with Hydrogen peroxide (GADOT), and a small hole was drilled using a fine drill burr (model 78001RWD Life Science). Using a microsyringe (33GA; Hamilton syringe) connected to an UltraMicroPump (World Precision Instruments) virus was subsequently injected at a flow rate of 100nl/min, following which the microsyringe was left in the tissue for 5-10 minutes after the termination of the injection before being retracted. For photometry experiments, an optic fiber ferrule (400um, 0.37-0.48 NA, Doric Lenses) was slowly lowered into the brain. The incision was closed using Vetbond bioadhesive (3M) and the skull was covered in dental cement and let dry. An RFID chip (ID-20LA, ID Innovations) was implanted subcutaneously. Mice were then disconnected from the anesthesia, and were administered with subcutaneous saline injection for hydration and an IP injection of the analgesic Rimadyl (Norbrook, 5 mg/kg) as they recovered under gentle heating.

In mice used for in-vivo electrophysiology recordings, we implanted a custom-made metal head bar, connected to the skull with dental cement.

Single molecule fluorescence in-situ hybridization (smFISH)

Numbers

Fentanyl n=11 sections from 2 mice (threshold of # of puncta for defining as *Fos*$^+$=7: n=2502 cells; threshold of # of puncta for defining as *Egr2*$^+$ puncta threshold=4: n=2564 cells). Saline n=10 sections from 2 mice (*Fos*$^+$ puncta threshold=7: n=870 cells; *Egr2*$^+$ puncta threshold=4: n=1140 cells).

Staining

The smFISH protocol was implemented according to manufacturer guidelines (ACD RNAscope fresh frozen tissue pretreatment and fluorescent multiplex assay manuals; catalog #320513, #320293; as in Gonzales et al.[61] 1hr after saline or fentanyl i.p. injection, mice were deeply anesthetized with isoflurane and decapitated. Their brains were then rapidly removed and briefly washed in ice-cold PBS. Brains were then placed in molds containing OCT embedding medium (Scigen Scientific Gardena, CA 90248 U.S.A) and snap frozen on dry ice. Embedded brains were sectioned on a Leica CM1950 cryostat into 14 μm sections, mounted onto SuperfrostTM Plus slides (#J1800AMNZ) and stored at -80°C. Adjacent sections (at ~1.09 ±0.1 mm from Bregma) were processed for probe hybridization. All probes and kits were purchased from Advanced Cell Diagnostics (Mm-Egr2 #407871, Mm-Fos-C3#316921-C3, Fluorescent Multiplex Reagent Kit #320850). Slides were counterstained with DAPI for 30s and coverslipped with mounting medium (LabVisionTM PermaFluorTM Aqueous Mounting Medium, #TA-030-FM). Sections were imaged with the Hermes high-definition cell-imaging system using a 10x 0.4NA and 40X 0.75NA objectives, to capture 5 Z-stack images in each of 4 different channels-475/28 nm (FITC), 549/15 nm (TRITC), 648/20 nm (Cy5) & 390/18 nm (DAPI). Single images for each channel were obtained using Maximum Intensity Z-projection in ImageJ. Maximum intensity images were merged, and the claustrum region was manually cropped using ImageJ according to the Franklin and Paxinos Mouse brain atlas, 4th edition.[62] Images were analyzed using the CellProfiler speckle counting pipeline.[63]

CellPress
OPEN ACCESS

Single nuclei RNA sequencing (snRNAseq)

Virus injection and claustrum dissection

RetroAAV-Ruby was injected to the ACC of 14 mice (Lateral-Medial= ±0.25, Anterior-Posterior= +1.1, Dorso-Ventral= -1.75 mm from Bregma). Two weeks following the stereotactic injection, all mice were handled for two days. Mice were then habituated to saline injections and the behavioral arena for 3 days; each mouse was injected with 200 ul i.p saline and placed in an open field arena for 20 minutes. On the day of the experiment, 7 mice were i.p injected with 0.3 mg/kg fentanyl (i.p.) and placed in the open field arena for 20 minutes. 1hr after the injection, mice were euthanized, and their brain was harvested. Claustrum (Ruby-expressing) tissue was dissected under a fluorescent stereoscope and flash frozen on dry ice. Control mice (n=7) were taken directly from the home cage and their claustrum similarly dissected.

Nuclei extraction

The claustra from each experimental group were defrosted on ice and homogenized with 1.5ml of lysis buffer (10mM Tris HCl pH 7.4, 10mM NaCl, 3mM MgCl2, 0.1% NP40, 40U/ml RNAse inhibitor). The homogenate incubated on ice for 5 min, followed by 5 min centrifugation at 500xg. Supernatant was then removed and washed with 5ml buffer and recentrifuged. The pellet was re-suspended in 100ul wash buffer (1% BSA in PBS X1, 40U/μl RNAse inhibitor) and passed through a 40 μm filter tip (BAH136800040, Merck). Nuclei were counted using Acridine Orange/PI stain (F23001, Biosystems). 10,000 nuclei from each treatment (control, fentanyl) were used for the next step.

10x Genomics chromium single-cell 3′ library construction

10,000 nuclei from each treatment were loaded onto a single channel of a 10x Chromium chip, which were run on the 10X Single Cell RNA-Seq Platform using the Chromium Single Cell 3′ Reagent Kit v3.1. Libraries were prepared following the manufacturer's protocol (10X Genomics, San Francisco, CA). Briefly, single nuclei were partitioned into nanoliter scale Gel Bead-In-EMulsion (GEMs) in the Chromium controller instrument, where cDNA of each sample share a common 10X barcode from the bead. Amplified cDNA was measured by Qubit HS DNA assay (Q32851, Thermo Fisher Scientific). This Whole Transcriptome Amplified (WTA) material was processed through 3′ V3.1 library construction, and subsequent libraries were quantified again by Qubit. Libraries from 2 channels were pooled and sequenced on the Illumina NextSeq Platform, for a target coverage of 100,000,000 reads per sample.

Analysis

Reads were aligned to the mouse transcriptome (10mm) using cellRanger [Cell Ranger 7.0.0] with the default settings. Ambient RNA was removed using the R [R version 4.2.0] package SoupX [SoupX_1.6.1.tar.gz] and doublets were identified using scrublet. All following analysis was performed with Seurat [Seurat v4.0]. Cells with <200 features, doublet score >0.2, mitochondrial RNA >5% or ribosomal RNA >2.5% were removed from further analysis. In total 10457 cells passed filtration (~80% of all cells). Counts were normalized using the LogNormalize method and scaled using ScaleData. Variable genes (n = 2000) used for PCA were obtained with Seurat via FindVariableGenes with the default settings. Clusters were obtained with Seurat via FindClusters (reduction.type = 'pca', dims. use = 1:19), using resolution = 1.4. This resulted in the identification of 30 clusters. Cluster marker genes were detected using FindAllMarkers with the default settings. Clusters were assigned to cell types based on the following marker genes: *Slc17a7* for glutamatergic neurons, *Gad1*&*Gad2* for GABAergic neurons, *Slc1a3*&*Slc1a2* for Astrocytes, *Plp1* for Oligodendrocytes, *Flt1* for Endothelial cells, *Lama1* for Fibroblasts, *Pdgfrb* for Pericytes and *Ptprc* for Microglia. Data was embedded in 2D by the UMAP algorithm for visualizations.

Immunohistochemistry

Acute fentanyl injections

Mice were habituated to i.p saline injections for 3 days. Each day, they were injected with saline (10 ml/kg) and immediately returned to their home-cage. On the fourth day, mice were injected with Fentanyl (0.3 mg/kg) or saline, immediately returned to their home-cage, and 90 minutes following injection anesthetized with a mix of Ketamine-Xylazine (150-200 microliters of Ketamine-Xylazine mixture (*i.p.*): Ketamine 42.5 mg/ml, Xylazine 1.5 mg/ml, in sterile saline). Then, trans-cardial perfusion was performed, with 20ml ice-cold PBS, followed by 20ml ice-cold PFA (4%). Brains were removed and fixed in 4% PFA overnight at 4°C.

Staining

On the following day, brains were thoroughly rinsed in a 0.9% NaCl Phosphate buffered saline (PBS) solution and sectioned on a Vibratome (7000 smz-2) at 40 μm thickness in the coronal plane. Six series of sections were collected from each brain, resulting in six copies of brain slices 240 μm apart.

In order to stain for c-Fos, floating section immunohistochemistry was performed. Sectioned brain slices were washed twice in PBS, followed by blocking in 3% normal horse serum and 0.3% Triton X-100 in PBS for 1 hour. Sections were then incubated overnight at 4°C in a rabbit anti-Fos primary antibody (Cat#226003 final dilution to 1:500 in 3% normal horse serum). 16 hr later the sections were washed three times for 10 minutes in PBS. Washes were followed by 2 hours of incubation at room temperature with donkey anti-rabbit IgG H&L Alexa Fluor 488 or 647 (Abcam; catalog No. ab150065; final dilution to 1:500) in 3% normal horse serum. Finally, the sections were washed once in PBS and then counterstained with DAPI (Roche; catalog No. 10-236-276; final dilution 1:1000 in PBS) to detect cell nuclei and then quickly washed once, mounted onto slides and covered.

Image acquisition

Slides were scanned on a high-speed fully-motorized multi-channel light microscope (Olympus IX-81) in the microscopy unit of the Edmond and Lily Safra Center for Brain Sciences. 10X magnification (NA = 0.3), 20X magnification (NA = 0.5) or 40X magnification (NA = 0.6). Green, red, blue and far-red channels exposure times were selected for optimal clarity and were kept constant within each

brain series. DAPI was acquired using excitation filters of 350 ± 50nm, emission 455 ± 50nm; GFP excitation 490 ± 20nm, emission 525 ± 36; mCherry/tdTomato excitation 555 ± 25nm, emission 605 ± 52nm.

Fiber Photometry during Self-Administration of Fentanyl vs Quinine

A commercially available fiber photometry system, Neurophotometrics FP3002, was used (Neurophotometrics LTD, San Diego, CA). In brief, recording was accomplished by providing a 415 nm and 470 nm excitation light through the patch-cord for calcium-independent and calcium-dependent fluorescence emission from GCaMP6s. Excitation power was set to 30% of the system maximal power in the 470 nm channel, and 15% of maximal power in the 415 nm channel. Recordings were performed with Bonsai open-source software[64] at 20 Hz per channel from both hemispheres.

For oral self-administration, we used two behavioral lick ports, one providing quinine, and the other providing fentanyl. Port location was swapped between consecutive sessions. The ports were connected via USB cables to a Bpod state machine (ports and state machines were purchased from Sanworks, Stony Brook, NY, USA). A custom protocol was built in Matlab to control liquid output. Each reward supplied by the system resulted in a TTL pulse that was sent from Bpod box (Bpod, Sanworks) to the digital input nodes of the FP3002 system, to enable accurate synchronization between the behavior and the recording. The inter-reward interval was defined as 3 seconds, to enable separation of the representation of consecutive rewards in the photometry signal. Reward size was 10 microliters.

The behavioral procedure for this experiment is as described in the table titled Behavioral Procedure, with some modifications. First, during the 3 days of quinine habituation (-3 to -1), mice were also habituated to the recording chamber and to attachment of the fiber optic to their implanted cannulas, for 10 minutes a day. Second, mice were water restricted, receiving liquids only in the recording chamber, which they accessed twice daily, for sessions lasting 15 minutes. Mice were monitored to ensure consumption of a minimum liquid intake of 1-1.5 ml day, supplemented on an individual basis if necessary.

Analysis of fiber-photometry data was performed using a custom Python code. To account for photo bleaching, 5th degree polynomials were fit to either GCaMP and isosbestic signals, substracted from the raw signal. Next, we calculated Pearson's correlation coefficient for the two adjusted traces, serving as a metric for the noise level. When Pearson's r was greater than 0.97, the session was removed from analysis due to unreliable signal. When the metric was smaller than 0.97 but larger than 0.25, we scaled the isosbestic trace to match the scale of the GCaMP signal, and subtracted it from the signal. Lastly, when the metric was smaller than 0.25, we did not subtract the isosbestic from the GCaMP signal, given that this subtraction could add noise to the signal.

We then subtracted the minimum value of GCaMP signal from the entire trace, in order to avoid negative values. Afterwards, we defined the change in fluorescence for each time-point (ΔF/F) using the following formula:

$$\frac{\Delta F}{F} = \frac{F - F_1}{F_1}$$

Where F_1 is the lowest percentile of signal in this session.

Lastly, to enable direct comparison between sessions, we calculated z-score values of ΔF/F for each session [z(ΔF/F)], as in Atlan et al.[17]

Optogenetic stimulation during oral self-administration

The behavioral protocol during optogenetic manipulation was similar to the one used for fiber photometry recordings, with a few modifications. Thus, two sessions of FR5 were performed each day, each lasting 15 minutes, spaced at least 4 hours apart. Optogenetic stimulation was performed in 2 of 8 sessions over 4 days, in order to avoid habituation to the laser. Liquid intake was maintained at a minimum of 1-1.5 ml / day, with mice provided with supplemental water, as needed. To increase the number of trials per session, reward size was reduced to 4 microliters, and trials were limited to 10 consecutive rewards, with a 15 seconds timeout instated following the 10th reward. To ensure that trials reflected a continuous effort to obtain reward, the maximal inter-poke interval permitted within a trial was of 1 second (i.e., a delay of >1 sec between pokes resulted in termination of the trial). Following delivery of reward, an interval of 1 sec was instated until pokes were counted for the next reward. Under these conditions, mice tended to persist at a port for a bout comprising multiple rewards, following which they tended to dissociate from the port, prior to re-engaging with the same or alternate port. Sessions were terminated after 15 minutes, since in early experiments, mice saturated their consumption within 13 minutes of session onset. We chose to perform manipulation experiments on ACCp and OFCp neurons together, as the ACCp neurons comprise only 9% of claustrum cells, OFCp neurons comprise 18% and the joint ACCp / OFCp population comprise another 9% (accumulating to 36% of claustral neurons). Furthermore, both populations were transcriptionally recruited by fentanyl (Figure 1), and both populations exhibited a dip in activity upon the consumption of the first reward in a trial, prolonged during bout consumption (Figure 2).

Stimulation

We used a commercially available LED (Cat #LEDFLS_450, Doric LTD, Franquet, Quebec, Canada), emitting light at a wavelength of 450nm. We used a light intensity of 7mW per hemisphere. Stimulation was triggered upon the first reward in a trial, randomly in 50% of trials. Stimulation lasted 2 seconds, at 20Hz (25 milliseconds ON – 25 milliseconds OFF).

Automated Cages Oral Self-Administration of Fentanyl

In order to assess animals' preference towards fentanyl, we developed a novel group-housed, automated self-administration setup. The setup allows for continuous access to the ports.

Apparatus

Cage

A circular hole (diameter - 40 mm) was drilled in one of the sides of a standard cage (34X19X19 cm), 30 mm above the cage's floor. A transparent tube (100 mm long, external diameter - 40mm, internal diameter - 34mm) connected the cage to two lick ports (Sanworks).

Hardware

An RFID reader was attached to the tube from the top, 4 cm from the holder (ID-20LA, ID Innovations). Each cage was connected to two behavioral lick ports (Sanworks), one that provided quinine, and the other providing fentanyl (the identity of each port was swapped daily. Ports were connected via USB cables (cat6) to a Bpod state machine (ports and state machines were purchased from Sanworks, Stony Brook, NY, USA).

The ports consist of 3 main parts: (1) Emitter and sensor of an infra-red beam. When the beam is interrupted by the mouse's tongue, the state machine counts it as a "lick". (2) LED (3) Pump that provides specific volumes of liquid. Ports were calibrated to output 10 microliters drops of either quinine-adulterated water or fentanyl.

Software

A custom Matlab script was written to support individualized tracking and control the difficulty level (Fixed Ratio, FR) of the task.

Behavioral Procedure

Days	Stage	Details and Specifications
-28	Stereotaxic injection and RFID implantation	as detailed
-3 to -1	Habituation to quinine adulteration (in homecage)	Regular acidic water is replaced with quinine (0.1 mg/ml) in acidic water. Mice are weighed daily.
1-2	Habituation to the Bpod system – Quinine	Mice are transferred to the homecage connected to the behavior port, while one of the ports is blocked. The other port provides quinine (0.1 mg/ml in acidic water), under a fixed ratio of 1 (FR1=1 lick results in 1 reward of liquid). The location of the open port is alternated daily.
3-4	Habituation to Fentanyl drinking	Quinine is replaced with fentanyl (0.1 mg/ml, in deionized water)
5-8	Fixed Ratio (FR) 1	Fentanyl is provided in one port, while quinine is provided on the other one, both under FR1.
9-12	FR3	The level of difficulty is increased to FR3 in both ports (3 licks=1 reward)
13-16	FR5	The level of difficulty is increased to FR5 in both ports (5 licks=1 reward); fentanyl dose is raised to 0.15mg/ml

Intermittent Access

Following the Behavioral Procedure in the operant arena, mice were exposed to 4 rounds of intermittent access. Each round comprised of 3 days of free access to water (in the home cage), followed by a choice session between fentanyl and water (after 12 hrs of water restriction).

Quinine Adulteration

Following the Behavioral Procedure in the home-cage. After 4 additional days of FR5 (0.1 mg/ml fentanyl vs 0.1 mg/ml quinine), we gradually added quinine to the Fentanyl bottle. Quinine doses in the fentanyl bottle were: 0.05, 0.1, 0.15, 0.2 and 0.25 mg/ml. Each dose was given for two days. Quinine dose in the quinine port remained constant (0.1 mg/ml).

Conditioned Place Preference

Apparatus

We followed the procedure of Terem et al.[42]

Each place conditioning apparatus consisted of an open field enclosed in separate light- and sound-attenuating chambers. General activity and location in the open field were monitored by video recording. The floor of the open field consisted of interchangeable halves made of one of two textures. The combination of floor textures was selected on the basis of calibration studies observing that mice spend an average of about 50% time on each floor type during preference tests. Thus, the apparatus is "unbiased". Specifically, the floors are either of a rough "crushed ice" texture, coupled with walls on which appear black dots, versus a smooth floor texture coupled with walls on which appear vertical black lines. Prior to and following each session the open field and floors were cleaned using a solution of 5% virusolve.

Procedures

An unbiased conditioning procedure consists of the following phases:

Handling: 5-min session for each mouse. *Pre-conditioning bias test*: a single 20 min test session is conducted during which mice are allowed to freely explore the open field with half of the arena containing the 'crushed ice' floor and dots walls and half of the arena containing the 'smooth' floor and straight lines walls. *Conditioning 1*: 3 days of 2 sessions/day. Mice are randomly assigned to one of two groups, pairing fentanyl (0.2 mg/kg IP) to either the 'crushed ice' or 'smooth' contexts, while saline conditioning (10 ml/kg, IP) occurred in the opposite context. In all sessions, mice have access to the entire apparatus with the same floor and walls type on both sides. *Post-conditioning bias test 1*: identical to *Pre-conditioning*. *Conditioning 2*: After the first post-conditioning bias test, we continued to 3 additional conditioning days, where fentanyl at a higher dose (0.5 mg/kg IP) was paired to same side as in the first conditioning days. *Post-conditioning bias test 2*: A second post-conditioning bias test was performed after this second round of conditioning.

In-vivo Electrophysiology Recording

Habituation

At least 3 weeks following viral injections, mice were habituated to head-fixation and to s.c. injections for 3 consecutive days. Each day, mice were briefly anesthetized using sevoflurane (Induction: 8%, Maintenance: 2-3%, SummnoSuite), and head-fixed on the recording setup. A Venflon (24G, DELTAVEN) needle was inserted subcutaneously on the backside of the mouse. Following 30 minutes of recovery, mice were head-fixed again, a tube was connected to the open end of the venflon, and mice received several saline injections (10 ml/kg) through the venflon. Injections were 10-20 minutes apart from one another. Mice were allowed to run on a treadmill while head-fixed.

Recording

On the day of recording, mice were anesthetized and implanted with a venflon. A small craniotomy was then drilled (model 78001RWD Life Science) in a region of the skull approximately 0.5 to 1 mm anterior, and -0.3 to 0.3 mm lateral to Bregma. The craniotomy was covered with a silicone elastomer (WPI; Kwik-Cast cat#KWIK-CAST), and mice were allowed at least 30 minutes to recover from anesthesia. Gentle heating was provided during the first few minutes of recovery.

Following recovery, mice were head-fixed again. The silicone cap was removed, and an electrical ground (Ag/AgCl) was located on the surface of the skull and covered with saline. A Neuropixels probe (imec, phase 3A) was lowered to the level of the skull in an angle of 20° relative to the anterior axis, and inserted through the craniotomy to a depth of 4.6 mm. Probes were covered with fluorescent dyes (DiI [Invitrogen cat#V22885] or DiO [Invitrogen cat#V22886]) before penetration, to enable reconstruction of penetration sites, following the procedure in Feigin et al.[65] Following insertion, we waited 20 minutes to allow the tissue to stabilize around the probe. Then, we started data acquisition. 20 minutes after the beginning of acquisition, we injected saline (10 ml/kg) through the venflon, to control for the effect of s.c. injections. 20 minutes later we injected Fentanyl (0.2 mg/kg, s.c). The recording lasted 30 minutes after fentanyl injection.

Analysis

All recordings were acquired using Neuropixels phase 3A probes (imec), a base-station connector (imec), and a commercially available NI board. Data was sampled at 30 kHz, with action potential band-filtered to contain 0.3-10 kHz. Data was acquired and saved using SpikeGLX software. Spike sorting was performed using Kilosort2[66]; https://github.com/MouseLand/Kilosort2). Manual sorting of the units found during spike sorting was done using 'Phy' open-source GUI (UCL; https://github.com/cortex-lab/phy).

Geometric fit to bout length

We fit a geometric distribution to bout consumption based on a single parameter, $p_{terminate}$, which represents the probability to terminate a bout following each reward. This parameter is fit to the results obtained from individual mice at each experimental condition.

Thus, the probability to terminate bout consumption after a single reward is equivalent to $p_{terminate}$. The probability to perform a bout of 2 rewards is $(1 - p_{terminate}) * p_{terminate}$, as the animal must persist after the 1st reward but terminate drinking following the 2nd. In a general form, the probability to perform a bout of size n is:

$$(1 - p_{terminate})^{n-1} * p_{terminate}$$

The resulting expression follows a geometric distribution. Hence, for each individual mice, we plotted the distribution of bout sizes and performed a geometric fit. Optimization of the parameter $p_{terminate}$ was performed using the *scipy* function *curve_fit*.

For optogenetic experiments, we performed 2 fits per liquid per mouse – one for laser-on trials and one for laser-off. For self-administration experiments in the homecage setup, drinking data from the 12 experimental days was pooled together. Fits were made per liquid, per mouse.

QUANTIFICATION AND STATISTICAL ANALYSIS

Full statistical information is provided in Table S1. Investigators were blinded to the manipulations that experimental subjects had received during behavioral testing, recordings, and data analysis. All molecular data were analyzed and graphed with R. All behavioral and photometry data were analyzed with Python. Data was primarily analyzed with ANOVA on linear mixed-effect models (LMMs). Paired comparisons were performed when appropriate. Z-test for proportions and the non-parametric Kruskal-Wallis test were used when appropriate. Differences were considered significant when $p < 0.05$. All pooled data are expressed as mean ± SEM.

Figures

Figures were prepared using Photoshop, Illustrator, and InDesign (Adobe, San Jose, CA). Figures showing stained brain tissue were adjusted using a uniform brightness/contrast mask created in ImageJ. Images were then scaled or cropped to improve data presentation and increase signal visibility. Digitization was performed from raw, non-adjusted images.

Current Biology, Volume *33*

Supplemental Information

Claustral neurons projecting

to frontal cortex restrict opioid consumption

Anna Terem, Yonatan Fatal, Noa Peretz-Rivlin, Hagit Turm, Shahar Shohat Koren, Danny Kitsberg, Reut Ashwal-Fluss, Diptendu Mukherjee, Naomi Habib, and Ami Citri

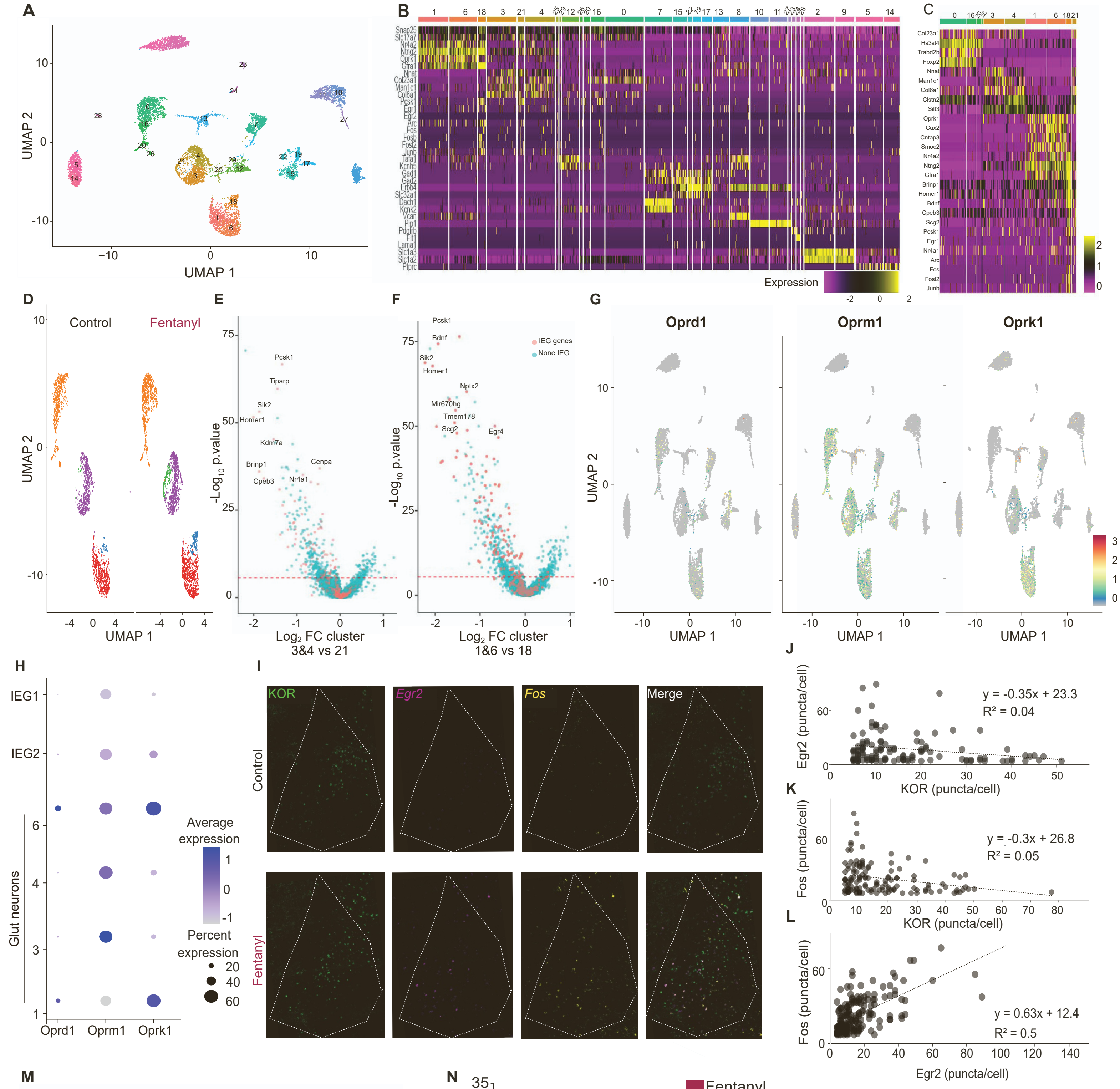

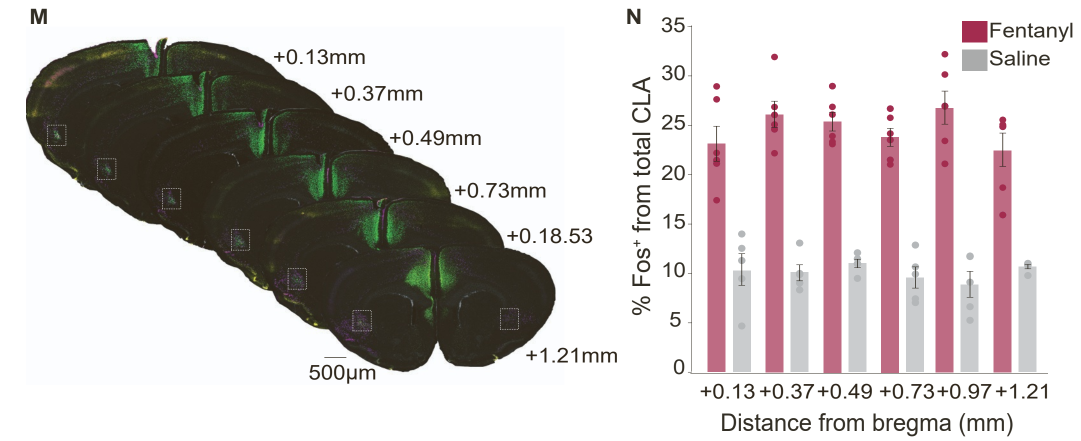

Figure S1. Supplementary analysis of claustral gene expression, Related to Figure 1. (**A-F**) (**A**) Performing UMAP dimensionality reduction on single-nuclei sequencing of claustrum neurons, we obtained 30 discrete clusters of cells. (**B**) Heatmap showing the expression profile of marker genes (rows) differentiating between clusters (columns). (**C**) Heatmap depicting marker genes defining the 3 classes of claustrum glutamatergic neurons, as well as genes induced within Glut IEG clusters (18,21). (**D**) IEG induction following fentanyl was observed in two sub-clusters of claustral glutamatergic neurons (green and blue dots relate to neurons associated with Glut IEG1 and Glut IEG2 clusters). The 3 parent clusters of glutamatergic neurons (labeled in orange, purple and red) are similar between control and fentanyl treatment. (**E**) Volcano plot, depicting genes differentially expressed between cluster 21 and the adjacent clusters 3,4. The Y axis depicts the -log p value (the dotted line corresponds to p=0.05), while the x axis depicts the log2 fold-change (FC) between clusters 3,4 and cluster 21. Differentially expressed genes are mostly upregulated in the IEG cluster. Red dots correspond to genes that are upregulated by activity in cultured neurons (data not shown) as an indication for the relation of the transcriptional differential between clusters 21 and 3,4 and gene inducibility. (**F**) as in (**E**) but for the comparison of cluster 18 to clusters 1,6. 89 of the top 100 differentially expressed genes in (**E**) are also found to be differentially expressed in (**F**), while 72 of the top 100 differentially expressed genes in (**F**) are also found to be differentially expressed in (**E**).

(**D**) Summary of virus expression and optic fiber locations in mice injected with retroAAV-CRE to the OFC, and AAV-DIO-GCaMP6s to the claustrum. The spread of infection was marked on histological sections, and digitally overlaid on an atlas image (n=5 mice). Right side – example image. (**E**) Overlayed average traces of ACCp signal corresponding to bouts of fentanyl consumption of different length (length=1, 1853 events; length=2, 619 events; length=3, 340 events; length=4, 198 events; length=5+, 714 events). As the delay between rewards in a bout could differ between trials, we averaged the signal around each reward in a ±1.5 seconds. Dotted lines extrapolate between rewards. (**F**) Overlayed average traces of ACCp signal corresponding to bouts of quinine consumption of different length (length=1, 1154 events; length=2, 134 events; length=3, 50 events; length=4, 36 events; length=5+, 69 events). (**G**) Average trace of the ACCp signal corresponding to a bout length=3, overlayed on the average trace of quinine bout length=3, illustrating the prolonged reduced activity in the ACCp activity between consecutive rewards in a bout. (**H**) Overlayed average traces of OFCp signal corresponding to bouts of fentanyl consumption of different length (length=1, 1082 events; length=2, 370 events; length=3, 261 events; length=4, 148 events; length=5+, 377 events). (**I**) Overlayed average traces of OFCp signal corresponding to bouts of quinine consumption of different length (length=1, 1206 events; length=2, 301 events; length=3, 140 events; length=4, 105 events; length=5+, 403 events). (**J**) Average trace of the OFCp signal corresponding to a bout length=3, overlayed on the average trace of quinine bout length=3, illustrating the recovery of the signal during both fentanyl and quinine consumption prior to each reward delivery. (**K-O**) The OFCp signal does not distinguish fentanyl from quinine consumption. (**K, M**) Heatmaps of OFCp signal during bouts of (**K**) fentanyl vs (**M**) quinine consumption of single rewards vs bouts (2+ rewards), in a window of ±5 second around the first reward (n=5 mice). (**L, N**) Average signal around single rewards compared to bouts of 2+ rewards. (**L**) Fentanyl; (**N**) Quinine. (**O**) Decrease in OFCp signal (average of 0 to +5 sec minus average of -5 to 0 sec) for single rewards vs bouts of consumption of fentanyl vs quinine. Liquid: $p=0.2630$, $F(1, 4)=1.694$; Is Bout: $p=0.0253$, $F(1, 4)=12.137$; Interaction: $p=0.8118$, $F(1, 4)=0.065$; ANOVA on LMM of delta signal ~ liquid * is bout | animal; n=5 mice.

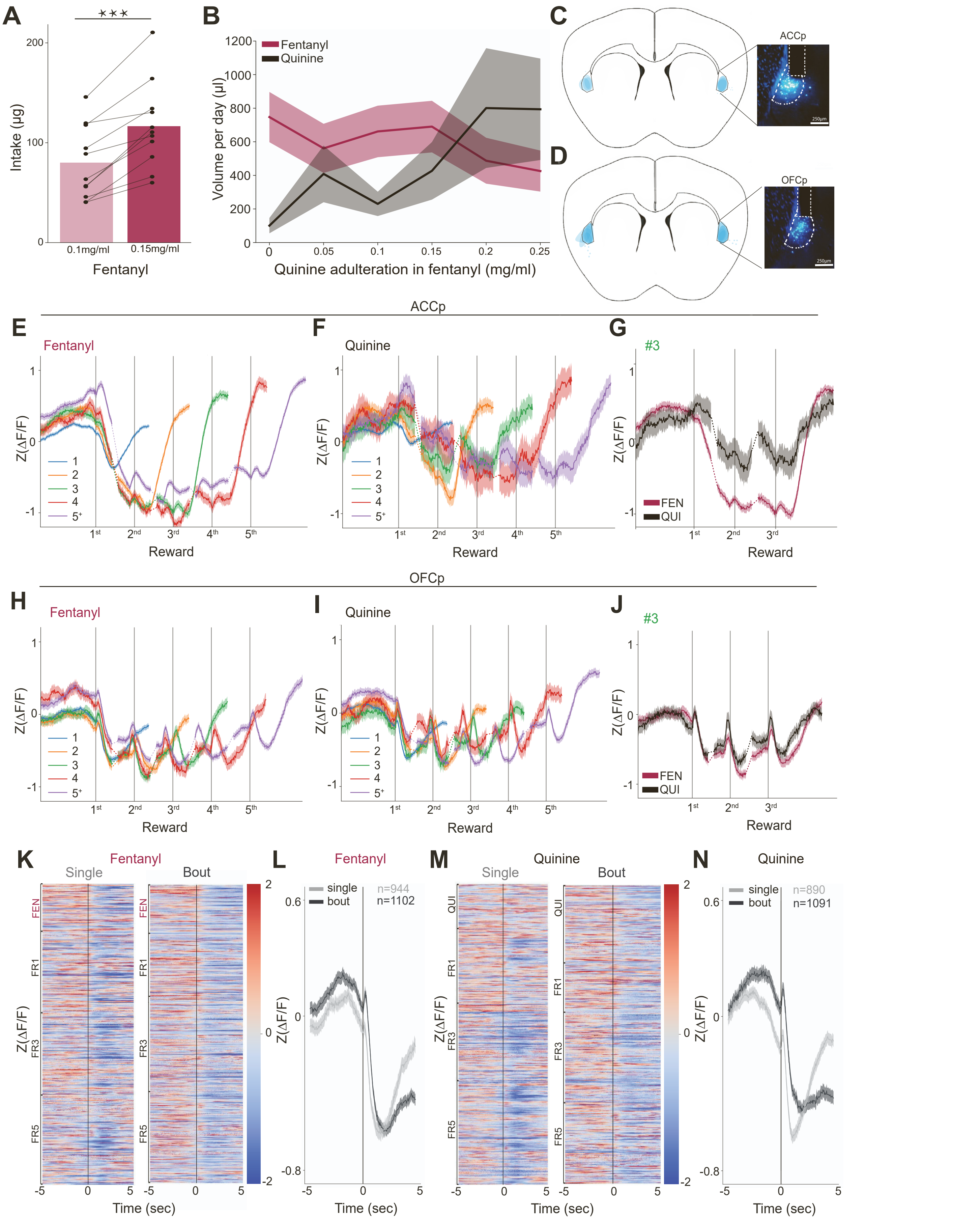

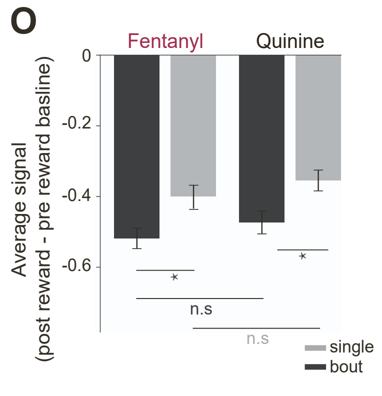

Figure S2. Supplementary analyses relating to fiber photometry recordings, Related to Figure 2. (**A-B**) Mice escalate consumption with increasing doses of fentanyl, and exhibit resilience to quinine-adulteration of fentanyl in the fiber photometry setup. (**A**) Average consumption of fentanyl at 0.1mg/ml = 79±11ug, fentanyl 0.15 mg/ml = 117±13ug: p=4.3e-05, t=6.877; paired t-test; n=11 mice. (**B**) Consumption of fentanyl (0.1 mg/ml) vs quinine (0.1 mg/ml) while escalating quinine adulteration of fentanyl (0, 0.1, 0.15, 0.2, 0.25 mg/ml). Only following addition of >0.2 mg/ml of quinine to fentanyl did mice reverse their fentanyl preference: p=0.0419, F(1, 4)=8.719; ANOVA on LMM of fentanyl volume ~ quinine dose | animal), while vehicle intake increased: p=0.0192, F(1, 4)=14.384; ANOVA on LMM of vehicle volume ~ quinine dose | animal); n=5 mice. ***p<0.001. (**C-J**) Relationship of bout consumption to signal deflection in ACCp & OFCp recordings. (**C**) Summary of virus expression and optic fiber locations in mice injected with retroAAV-CRE to the ACC, and AAV-DIO-GCaMP6s to the claustrum for the experiment shown in figure 2. The spread of infection was marked on histological sections, and digitally overlaid on an atlas image (n=5 mice). Right side – example image.

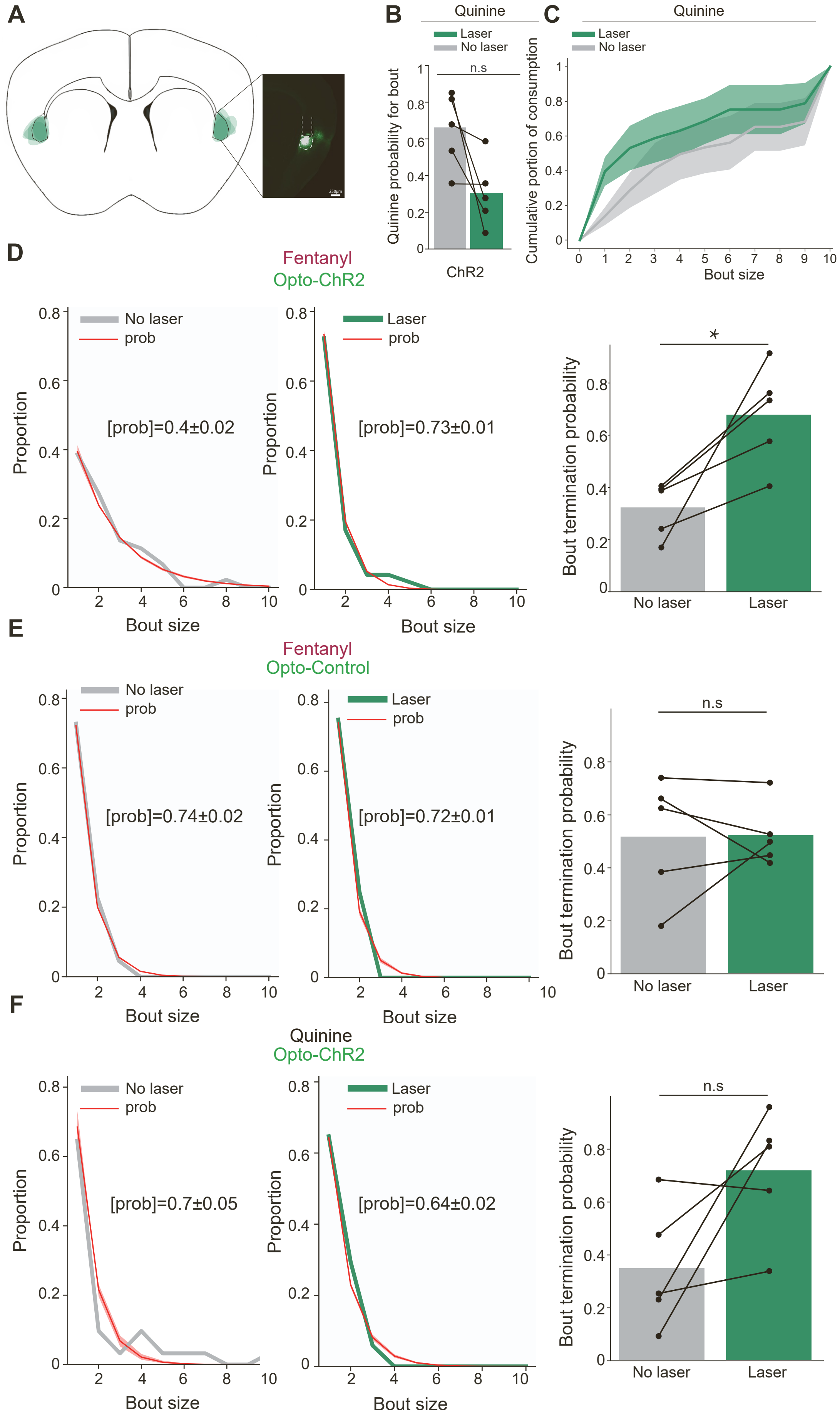

Figure S3. Optogenetic activation of frontal-projecting claustral neurons reduces bouts of quinine consumption, Related to Figure 3. (**A**) Summary of virus expression and optic fiber locations in mice injected with retroAAV-CRE to the ACC&OFC, and AAV-DIO-ChR2 to the claustrum for the experiment shown in **figure 3**. The spread of infection was marked on histological sections, and digitally overlaid on an atlas image (n=5 mice). Right side – example image. (**B**) Optogenetic activation reduces the probability that an individual trial of quinine consumption will result in a bout of consumption (2^+ rewards). p=0.0687, t=2.473; paired t-test; n=5 mice. (**C**) Cumulative distribution plot of quinine consumption (volume) as a function of bout length. Optogenetic stimulation caused mice to achieve more of their consumption in short bouts. (**D-F**) Modeling the distribution of bout lengths, by fitting a geometric function to each mouse in each condition. Each panel includes examples of individual mice, illustrating the proportion of bouts of different lengths (in grey or green) overlaid with the fit of a geometric function with a constant probability to terminate a bout (red). The probabilities are illustrated on the graphs as '[prob]'. On the right is the summary data, illustrating the bout termination probability for each individual mouse and the averages for the cohort. (**D**) Geometric functions fit to the consumption of fentanyl in mice expressing ChR2. p=0.0261, t=3.447; paired t-test; n=5 mice. (**E**) Geometric functions fit to the consumption of fentanyl in control mice. p=0.9659, t=0.045; paired t-test; n=5 mice. (**F**) Geometric functions fit to the consumption of quinine in mice expressing ChR2. p=0.0835, t=2.294; paired t-test; n=5 mice. n.s. non-significant, *p<0.05.

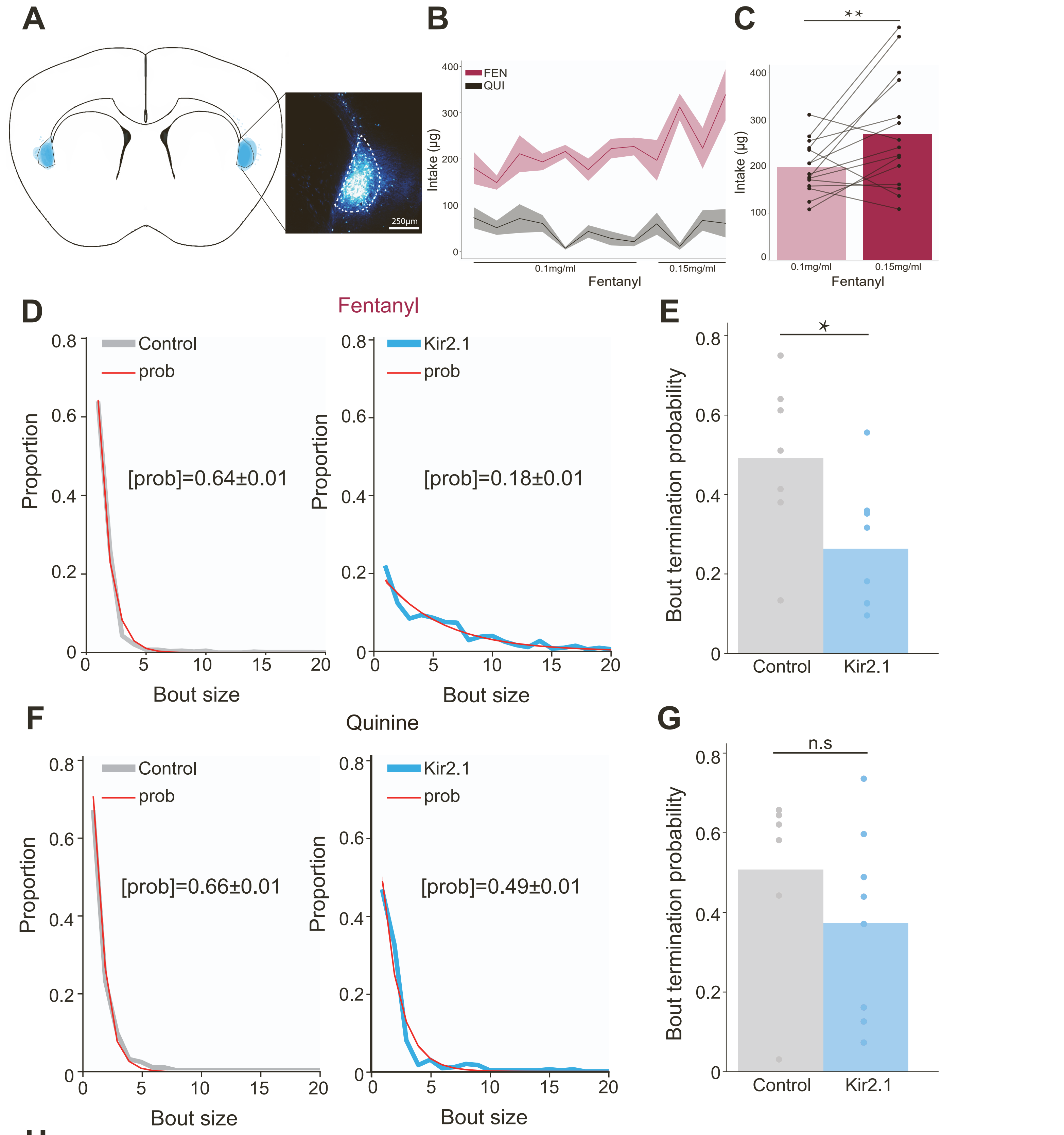

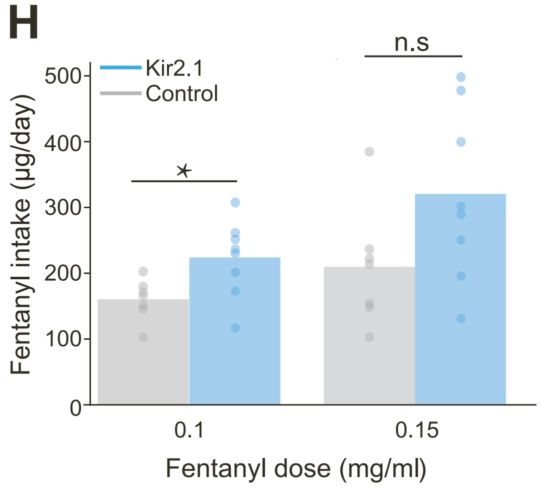

Figure S4. Mice escalate consumption with increasing doses of fentanyl in the automated cages, Related to Figure 4. (**A**) Summary of virus expression in mice injected with retroAAV-CRE to the ACC & OFC, and AAV-DIO-Kir2.1 to the claustrum for the experiment shown in **Figure 4**. The spread of infection was marked on histological sections, and digitally overlaid on an atlas image (n=8 ACC&OFC$^{Kir2.1}$ mice). Right side – example image. (**B-C**) Data illustrates the results for escalating doses of fentanyl (0.1 vs 0.15 mg/ml) consumption vs Quinine (0.1 mg/ml quinine) in the automated cages (n=15 mice). (**B**) Continuous graph of daily consumption. (**C**) Average daily consumption of fentanyl 0.1mg/ml = 197±9ug, fentanyl 0.15 mg/ml = 256± 23ug. p=0.0054, F(1, 14)=10.811; ANOVA on LMM – volume ~ dose | animal; n=15 mice. (**D-G**) Modeling the distribution of bout lengths, by fitting a geometric function to each mouse. (**D,F**) Each panel includes examples of individual mice (Kir2.1 vs control), illustrating the proportion of bouts of different lengths (in grey or blue) overlaid with the fit of a geometric function with a constant probability to terminate a bout (red). The probabilities are illustrated on the graphs as '[prob]'. (**D**) Geometric functions fit to the consumption of fentanyl in mice expressing Kir2.1 vs control. (**E,F**) Summary data, illustrating the geometric fit for each individual mouse and the averages for the cohort. (**E**) Fentanyl p=0.0310, t=2.418, t-test. (**F**) Quinine, p=0.2808, t=1.125; t-test. (**H**) Average daily consumption of fentanyl 0.1mg/ml, fentanyl 0.15 mg/ml (as in panel C), separated by stages for Kir2.1 vs control. 0.1mg/ml: p=0.0250, t=2.533; 0.15mg/ml: p=0.0878, t=1.771; t-test; n=8 Kir2.1 mice, n=7 control mice. n.s. non-significant, *p<0.05, **p<0.01.

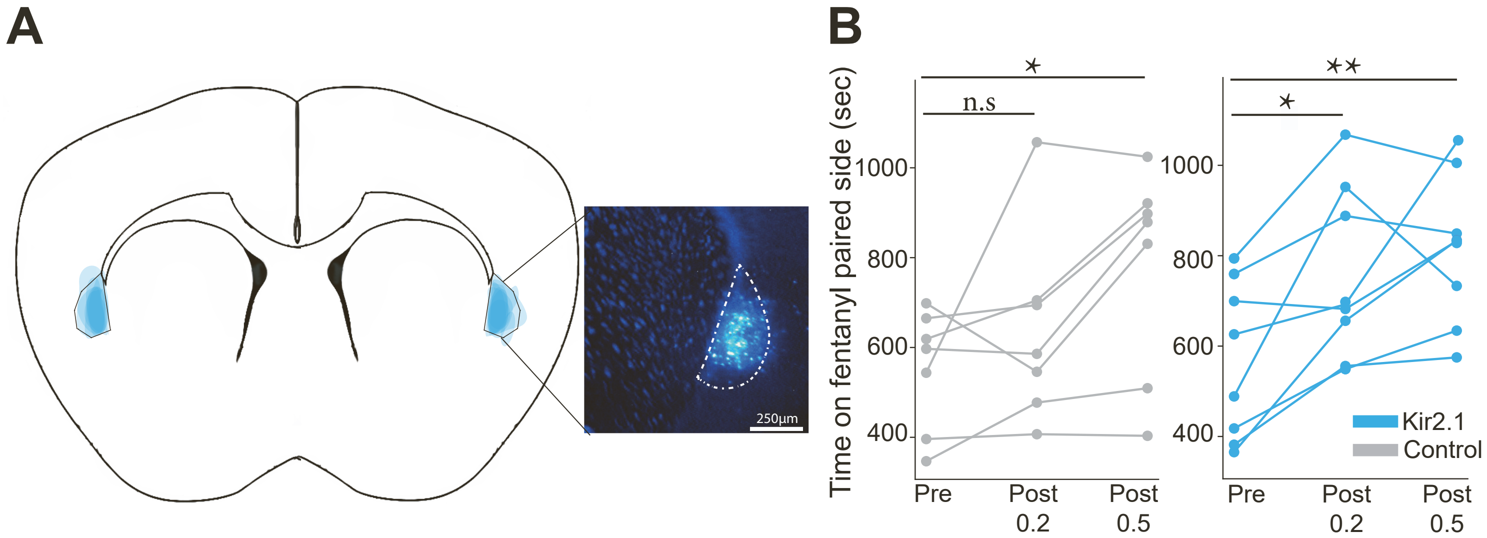

Figure S5. Fentanyl conditioned-place preference - time spent in fentanyl-paired side, Related to Figure 5. (**A**) Summary of virus expression in mice injected with retroAAV-CRE to the ACC & OFC, and AAV-DIO-Kir2.1 to the claustrum for the experiment shown in **Figure 5**. The spread of infection was marked on histological sections, and digitally overlaid on an atlas image (n=8 mice). Right side – example image. (**B**) Individual mice are depicted as connected dots, demonstrating time spent on the fentanyl paired side before exposure to fentanyl (Pre) and following exposure to low (0.2 mg/kg) and high (0.5 mg/kg) fentanyl concentrations. Kir2.1: low-dose, $p=0.0188$, $t=3.547$; high-dose, $p=0.0022$, $t=5.289$; paired t-test. Control: low dose, $p=0.6250$, $t=1.102$; high-dose, $p=0.0136$, $t=4.04$; paired t-test. Left: control, n=7 mice, Right: ACCp&OFCp$^{Kir2.1}$, n=8, paired t-test. n.s.=non-significant, *$p<0.05$, **$p<0.01$.

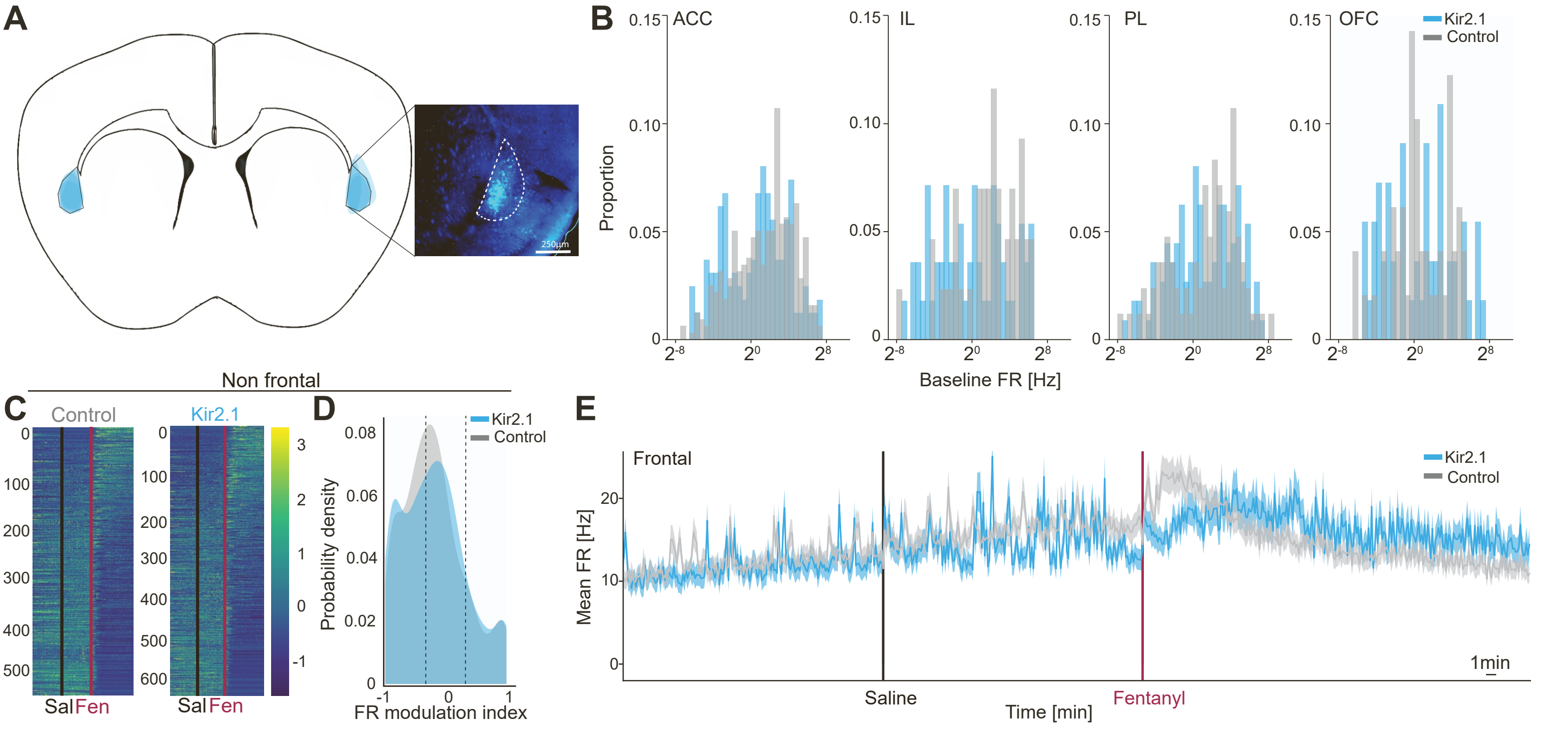

Figure S6. Inhibition of frontal-projecting claustral neurons specifically affects fentanyl modulation of the ACC & IL, Related to Figure 6. (**A**) Summary of virus expression in mice injected with retroAAV-CRE to the ACC&OFC, and AAV-DIO-Kir2.1 to the claustrum for the experiment shown in **Figure 6**. The spread of infection was marked on histological sections, and digitally overlaid on an atlas image (n=5 mice). Right side – example image. (**B**) Histograms of the distribution of firing rate (FR) at baseline, across different frontal structures (ACC = anterior cingulate cortex; IL = infralimbic cortex; PL = prelimbic cortex; OFC = orbitofrontal cortex), comparing ACCp&OFCp$^{Kir2.1}$ mice to control mice. Baseline FR was reduced in the ACC (p=0.0050, k-stat=7.883) and IL (p=0.0084, k-stat=6.952) of Kir2.1 animals, and unaffected in the PL (p=0.7737, k-stat=0.083) and OFC (p=0.6602, k-stat=0.193); Kruskal-Wallis test. (**C**) Heatmaps depicting normalized firing rates of individual units as a function of time. Units shown are located in non-frontal regions. (left) Control mice. (right) Experimental mice, expressing Kir2.1 in frontal-projecting claustral neurons. The black and red vertical lines denote the time of saline and fentanyl s.c. injection. (**D**) Distribution of fentanyl modulation indices, in recordings of non-frontal units from experimental (ACCp&OFCp$^{Kir2.1}$-expressing) mice vs control mice. Control: -0.19±0.02, Kir2.1: -0.18±0.02; p=0.73, K-stat=0.122; Kruskal-Wallis. (**E**) Mean firing rate (FR) of frontal units throughout the recording (20 min baseline, 20 min post-saline, 30 minutes post-fentanyl), illustrating the brief response of frontal units in the ~5 minutes post-fentanyl exposure in control mice, absent in experimental (ACCp&OFCp$^{Kir2.1}$) mice. In addition, the prolonged excitation of frontal units in experimental mice is observed, contrasting with the reduced activity of frontal units in

Figure	Mice, Groups, Group sizes	Statistical analysis
1B, D	Fentanyl; n=11 sections from 2 mice;<br>Saline; n=10 sections from 2 mice	Fos: control: 6±0.5%, fentanyl: 12.5±1.3%, $F(1, 2)=25.132$, *p=0.0375, ANOVA on linear mixed-effect model (LMM) of %IEG ~ treatment | animal.<br>Egr2: control: 7.8±0.5%, fentanyl: 12.9±0.7%, $F(1, 2)=42.807$, *p=0.0226, ANOVA on LMM of %IEG ~ treatment | animal.
1L	Fentanyl n=6 mice, n=70 sections;<br>Saline n=5 mice, n=56 sections	ANOVA on LMM of percent_positive ~ ap_loc + reference_total | animal<br>(reference_total – percent from DAPI or from FOS)<br>ACC, not OFC: $F(1, 5)=45.212$, **p=0.0011<br>ACC & OFC: $F(1, 5)=95.837$, ***p=0.0002<br>OFC, not ACC: $F(1, 5)=32.141$, **p=0.0024<br>Complement: $F(1, 5)=231.209$, ***p=2.2e-05
2C	n=11 mice; ACCp n=5, OFCp n=6	Liquid: $F(1,10)=93.182$, ***p=2.2e-06, Stage: FR3 – $F(1, 10)=4.315$, p=0.0645 ;FR5 – $F(1, 10)=3.512$, p=0.0904.<br>ANOVA on LMM of Volume ~ Liquid + Stage | Animal
2D	OFCp n=6 mice	Fentanyl intake:<br>$F(1, 5)=16.196$, *p=0.0101, ANOVA on LMM of amount ~ dose | animal<br>Water intake:<br>$F(1, 5)=16.541$, **p=0.0097, ANOVA on LMM of volume ~ dose | animal
2E	n=11 mice; ACCp n=5, OFCp n=6	ANOVA on LMM of %drinking in single rewards ~ liquid | animal. $F(1, 10)=105.837$, ***p=1.2e-06
2M	ACCp n=5 mice	ANOVA on LMM of delta_signal ~ liquid * is_bout | animal.<br>Liquid: $F(1, 4)=18.230$, *p=0.0130;<br>Is_Bout: $F(1, 4)=222.709$, ***p=1.2e-04; Interaction (liquid * is_bout): $F(1, 4)=15.291$, *p=0.0174
3C	ChR2 n=5 mice<br>Control n=5 mice	ChR2: t=3.378, p=0.0278, paired t-test<br>Control: t=0.200, p=0.8512, paired t-test
4D	Kir2.1 n=8 mice, Control n=7 mice	$F(1, 13)=6.944$, *p=0.0206, ANOVA on LMM of Volume ~ Group | Animal
4E	Kir2.1 n=8 mice, Control n=7 mice	$F(1, 13)=0.0001$, n.s, p=0.9924, ANOVA on LMM of Volume ~ Group | Animal
4H	Kir2.1 n=8 mice, Control n=7 mice	Single: t=2.415, *p=0.0312, Bout: t=3.992, **p=0.0015, t-test

4I	Kir2.1 n=8 mice, Control n=7 mice	Single: t=1.447, n.s, p=0.1716, Bout: t=0.304 n.s, p=0.7660, t-test
5C	Kir2.1 n=8 mice	Kir2.1: low-dose, t=3.547, *p=0.0188; high-dose, t=5.289, **p=0.0022; paired t-test
5C	Control n=7 mice	Control: low dose, t=1.102, n.s., p=0.6250; high-dose, t=4.04, *p=0.0136; paired t-test
6H	Kir2.1 n=429 units, Control n=532 units	Kruskal-statistic=40.271, ***p=2.2e-10. Kruskal-Wallis test.
6I	Kir2.1 n=5 mice, Control n=4 mice	Frontal units: Upregulated X-stat=27.505, ***p=1.6e-07; Chi-squared test<br>Downregulated X-stat=19.922, ***p=2.2e-10; X^2-test for proportions.
6J	Kir2.1 n=5 mice, Control n=4 mice	Other units: Upregulated X-stat=6.4e-06, p=0.9980; Chi-square test<br>Downregulated X-stat=0, p=1<br>X^2-test for proportions.
6K	Kir2.1 n=5 mice, Control n=4 mice	X^2-test for proportions.<br>**ACC**<br>Down; Control=133/317, Kir2.1=43/162, stat=-3.31, ***p=0.0009.<br>Up, Control=38/317, Kir2.1=48/162, stat=4.76, ***p=2e-06.<br>**IL**<br>Down, Control=13/43, Kir2.1=6/56, stat=-2.444, *p=0.0145.<br>Up, Control=11/43, Kir2.1=26/56, stat=2.125, *p=0.0336.<br>**PL**<br>Down, Control =16/84, Kir2.1=24/112, stat=0.409, p=0.6823.<br>Up, Control =30/84, Kir2.1=29/112, stat=-1.483, p=0.1380.<br>**OFC**<br>Down, Control =10/49, Kir2.1=8/55, stat=-0.789, p=0.4302.<br>Up, Control =11/49, Kir2.1=21/55, stat=1.735, p=0.0823.
Supplemental Figures		
S1N	Fentanyl n=6 mice, 70 sections, Saline n=5 mice, 56 sections	ANOVA on LMM of percent _*Fos*$^+$ ~ ap_loc + treatment | animal Treatment: F(1, 9)=309.635, ***p=2.8e-08; AP position: F(1, 9)=0.166, n.s., p=0.6932;
S2A	n=11 mice; ACCp n=5, OFCp n=6	t=6.877, p=4.3e-05; paired t-test; n=11 mice
S2B	n=5 mice	Quinine adulteration experiment.<br>ANOVA on LMM of fentanyl volume ~ quinine dose | animal. F(1, 4)=8.719, *p=0.0419.<br>ANOVA on LMM of vehicle solution volume ~ quinine dose | animal. F(1, 4)=14.384, *p=0.0192

S2O	OFCp, n=5 mice	LMM of delta_signal ~ is_bout * liquid | animal<br>Liquid $F(1, 4)=1.694$, $p=0.2630$<br>Is_Bout $F(1, 4)=12.137$,*$p=0.0253$<br>Interaction $F(1, 4)=0.065$, $p=0.8118$
S3D	ChR2, n=5 mice	No laser, [prob]=0.320±0.048,<br>Laser [prob]=0.678±0.087,<br>t=3.447, *p=0.0261, paired t-test
S3E	Tdt, n=5 mice	No laser, [prob]=0.518±0.103,<br>Laser [prob]=0.523±0.053,<br>t=0.045, p=0.9659, paired t-test
S3F	ChR2, n=5 mice	No laser, [prob]=0.348±0.104,<br>Laser [prob]=0.717±0.107,<br>t=2.294, p=0.0835, paired t-test
S4C	n=15 mice:<br>(n=8 ACC&OFC$^{Kir2.1}$<br>n=7 ACC&OFC^{eGFP})	ANOVA on LMM of fentanyl amount ~ dose | animal.<br>**p=0.0054, $F(1, 14)=10.812$, **p=0. $F(1, 14)=10.812$
S4E	Kir2.1 n=8 mice,<br>Control n=7 mice	Fentanyl, Kir2.1 [prob]=0.264±0.056, GFP [prob]=0.491±0.077, t=2.418, *p=0.0310, t-test
S4G	Kir2.1 n=8 mice,<br>Control n=7 mice	Quinine, Kir2.1 [prob]=0.374±0.084, GFP [prob]=0.508±0.084, t=1.125, p=0.2808, t-test
S4H	Kir2.1 n=8 mice,<br>Control n=7 mice	0.1 mg/ml: t=2.533, *p=0.0250<br>0.15 mg/ml: t=1.771, p=0.0878<br>t-test
S5B	Kir2.1 n=8 mice<br>Control n=7 mice	Kir2.1: low-dose, t=3.547, *p=0.0188; high-dose, t=5.289, **p=0.0022; paired t-test<br>Control: low dose, t=1.102, n.s., p=0.6250; high-dose, t=4.04, *p=0.0136; paired t-test
S6B	Kir2.1 n=5 mice,<br>Control n=4 mice	**ACC**: k-stat=7.883, **p=0.0050<br>**IL**: k-stat=6.952, **p=0.0084<br>**PL**: k-stat=0.083, p=0.7737<br>**OFC**: k-stat=0.193, p=0.6602<br>Kruskal-Wallis test
S6D	Kir2.1 n=5 mice,<br>Control n=4 mice	Average fentanyl modulation index in non-frontal regions.<br>control: -0.19±0.02, Kir2.1: -0.18±0.02;<br>K-stat=0.122, p=0.73.<br>Kruskal-Wallis test.

TABLE S1.

Statistical analyses, Related to Figures 1, 2, 3, 4, 5, 6 and S1-S6.
